# Supplementary material for: Polyaromatic molecular peanuts
Source: Nat Commun. 2017 Jun 28;8:15914. doi: 10.1038/ncomms15914 (PMC5493752; doi:10.1038/ncomms15914)
Supplement: Supplementary Information [file ncomms15914-s1.pdf]

Type of file: PDF
Title of file for HTML: Supplementary Information
Description: Supplementary Figures, Supplementary Table, Supplementary Methods, and Supplementary References.

Type of file: PDF
Title of file for HTML: Peer Review File
Description:

Supplementary Figures

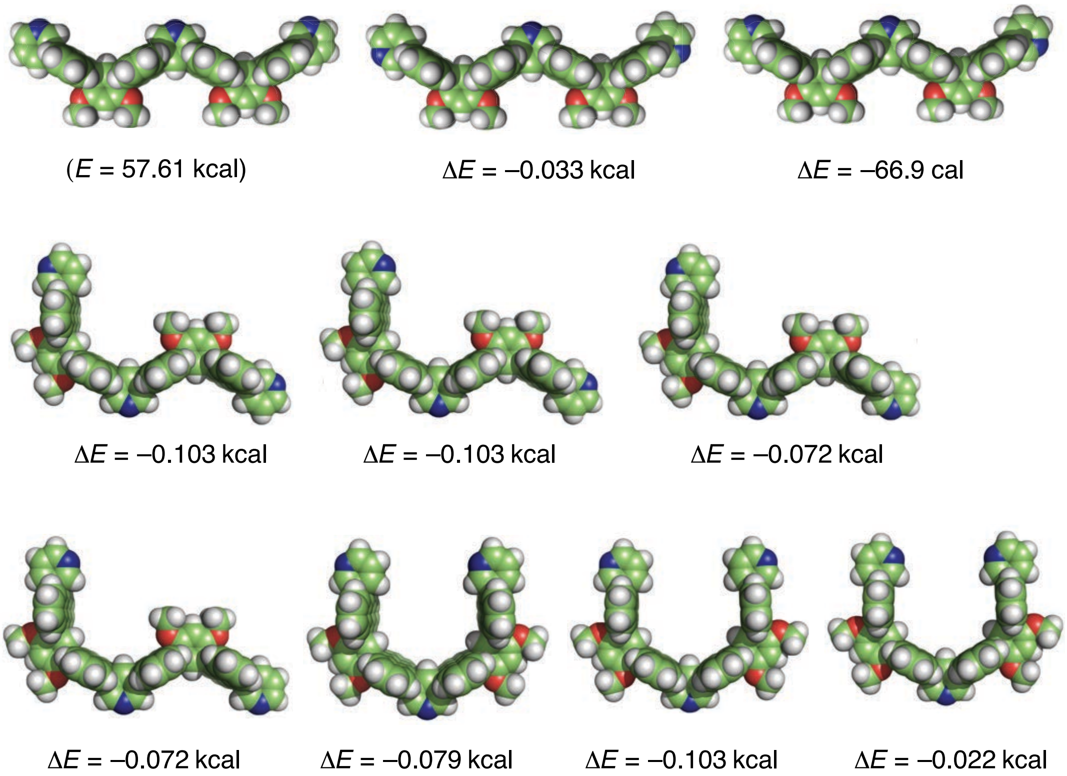

Supplementary Figure 1 | Optimized structures and energies (based on the upper-left structure) of ten stereoisomers of ligand **1** (R = -OCH_3) (PM6 calculations).

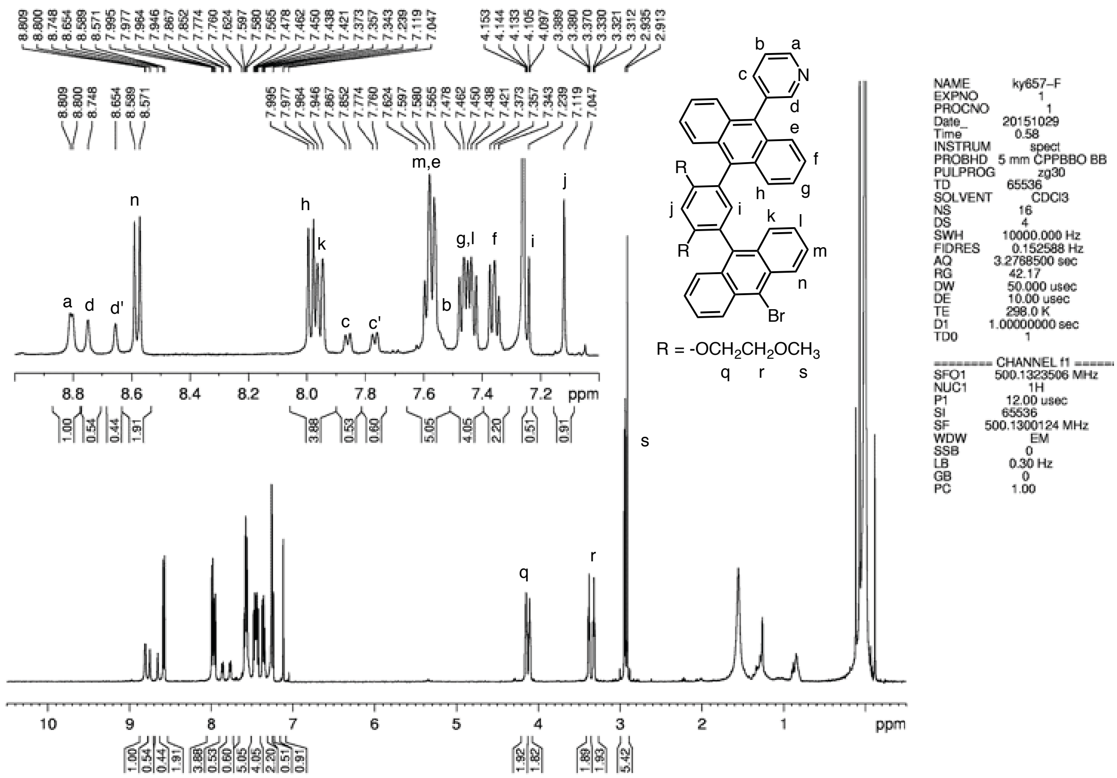

Supplementary Figure 2 | ^{1}H NMR spectrum (500 MHz, $CDCl_3$, room temperature) of 6a.

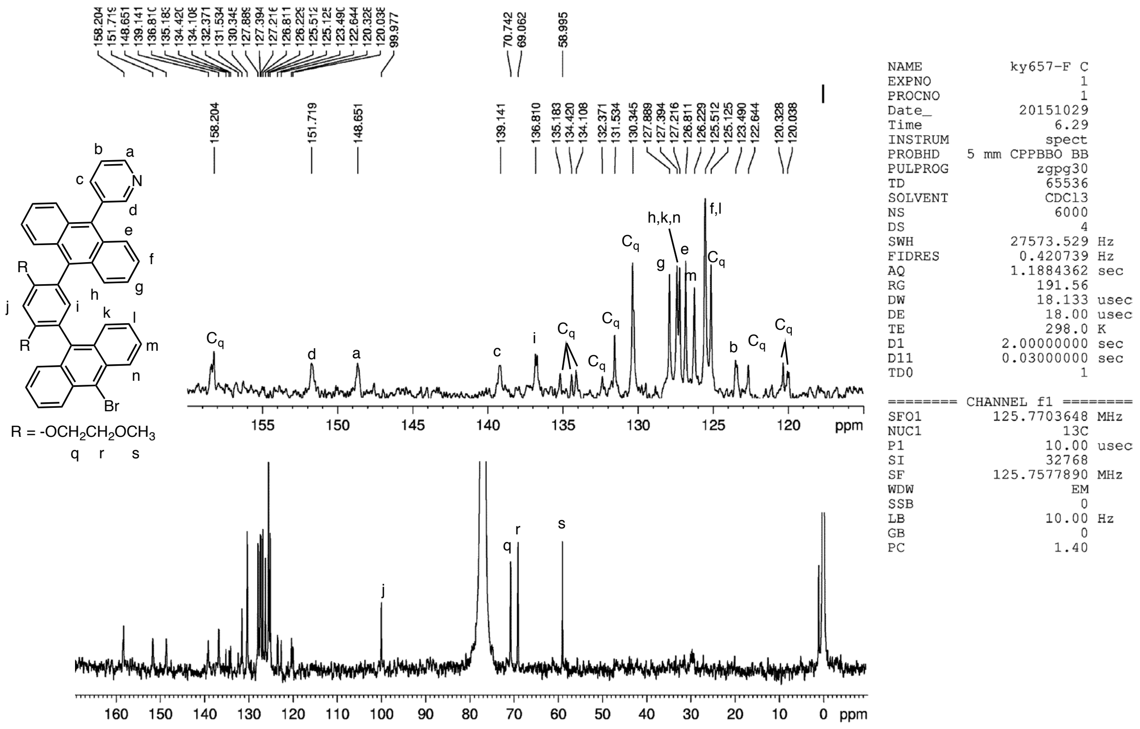

Supplementary Figure 3 | ^{13}C NMR spectrum (125 MHz, $CDCl_3$, room temperature) of 6a.

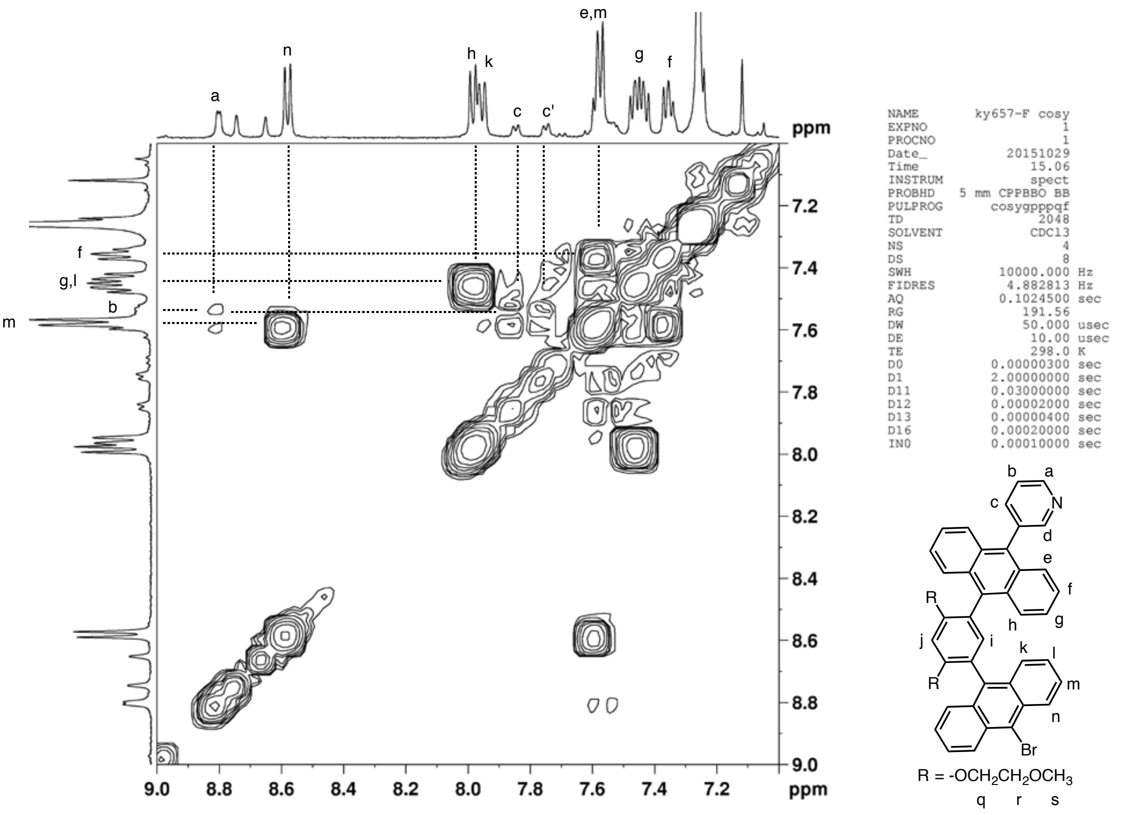

Supplementary Figure 4 | HH COSY spectrum (500 MHz, $CDCl_3$, room temperature) of **6a**.

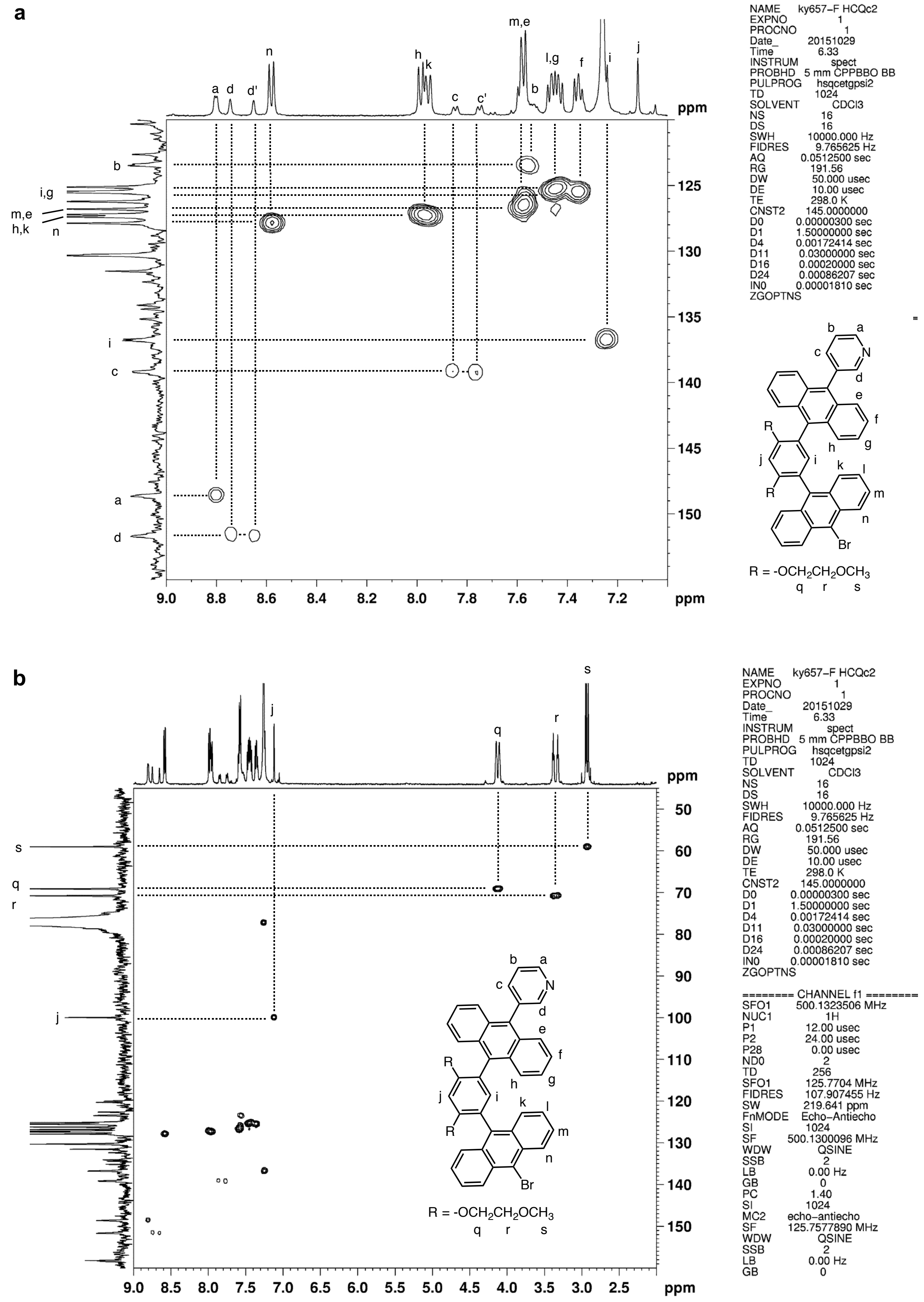

Supplementary Figure 5 | ** HSQC spectra (500 MHz, $CDCl_3$, room temperature) of **6a. (a) The aromatic and (b) aliphatic regions.

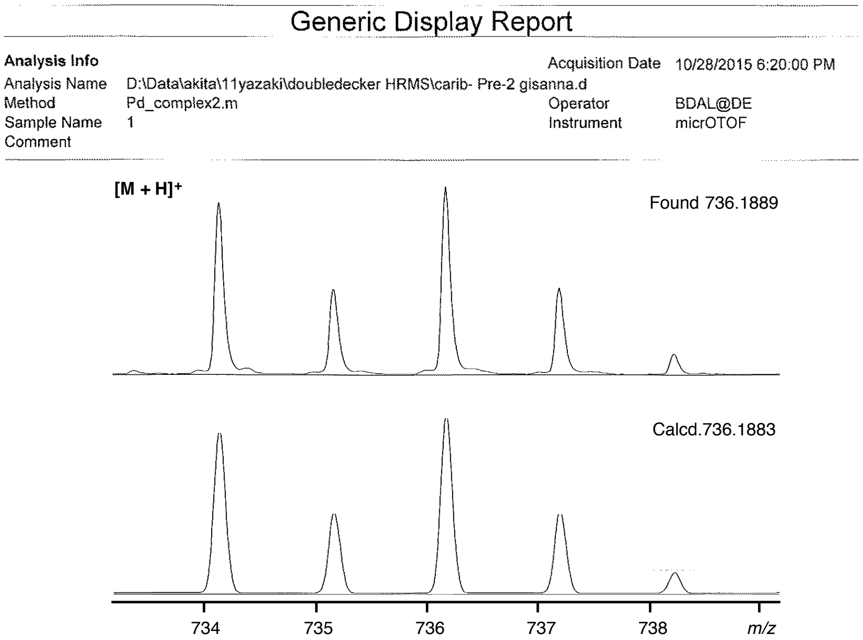

Supplementary Figure 6 | HR MS spectrum (ESI) of 6a.

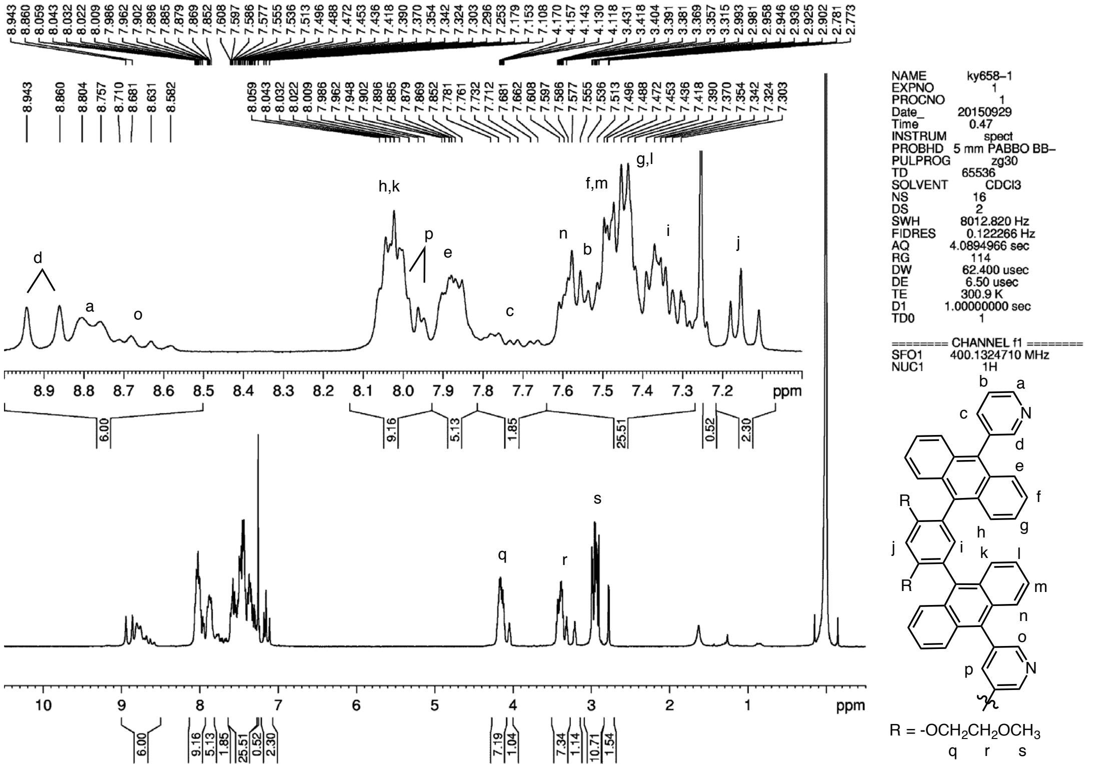

Supplementary Figure 7 | ^{1}H NMR spectrum (500 MHz, $CDCl_3$, room temperature) of 1a.

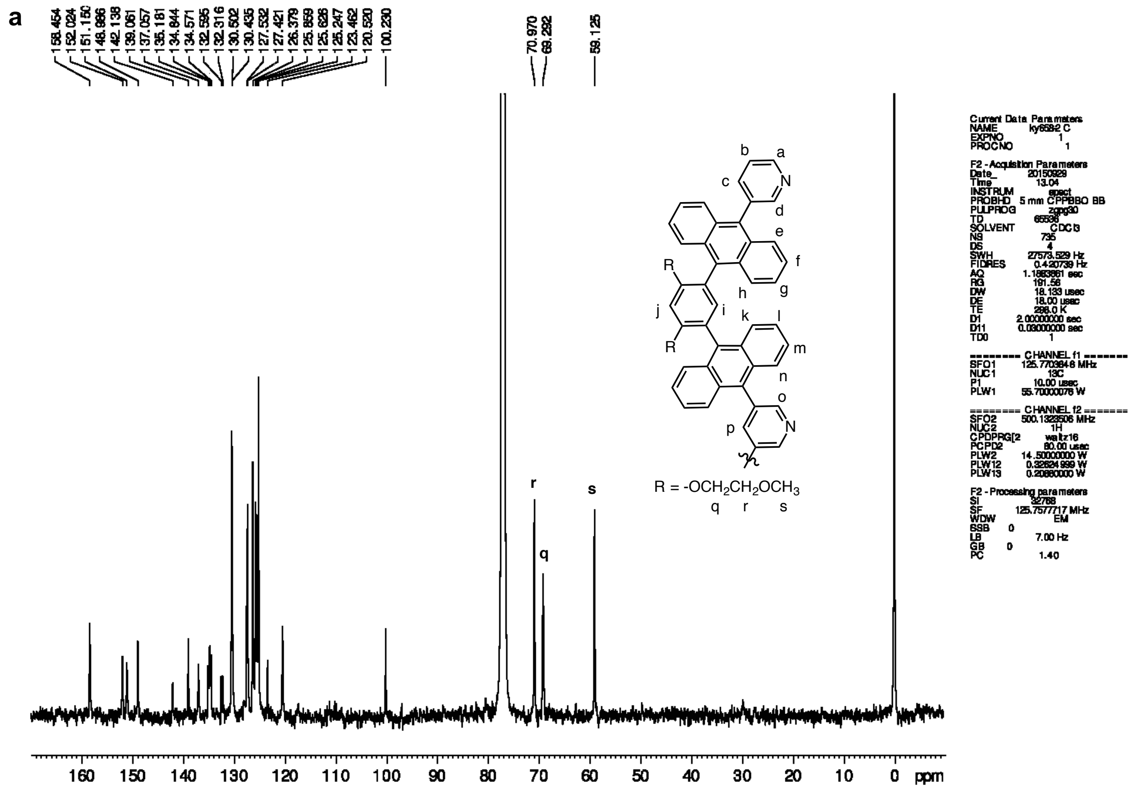

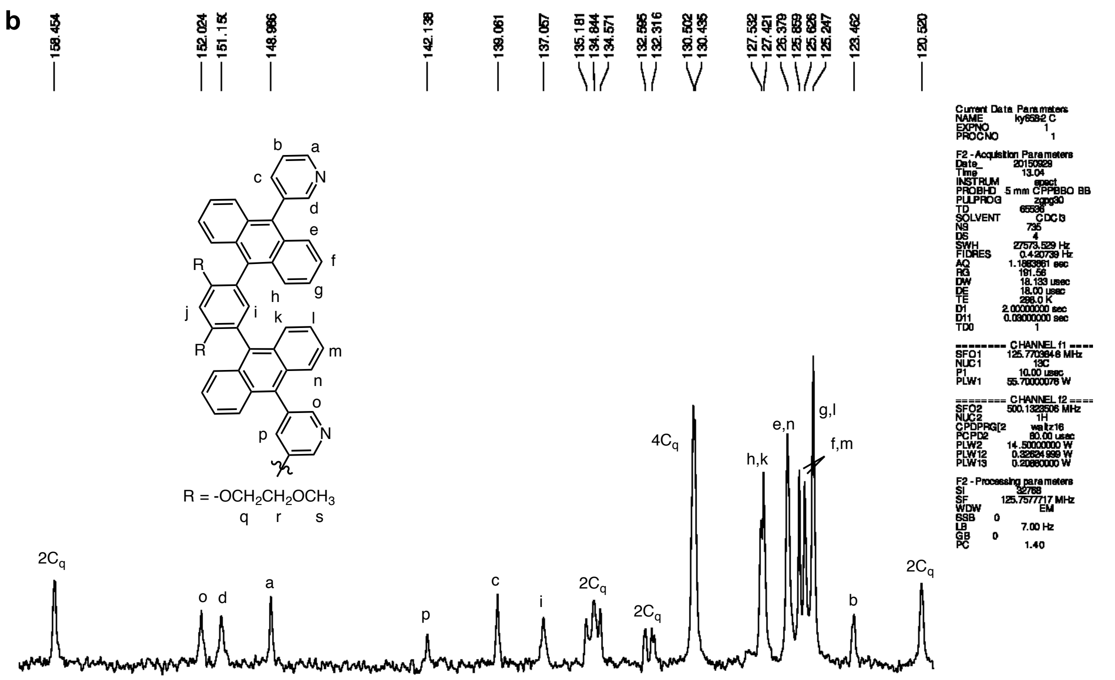

Supplementary Figure 8 | ^{13}C NMR spectra (125 MHz, $CDCl_3$, room temperature) of **1a**. (a) The aromatic and aliphatic and (b) aromatic regions.

a

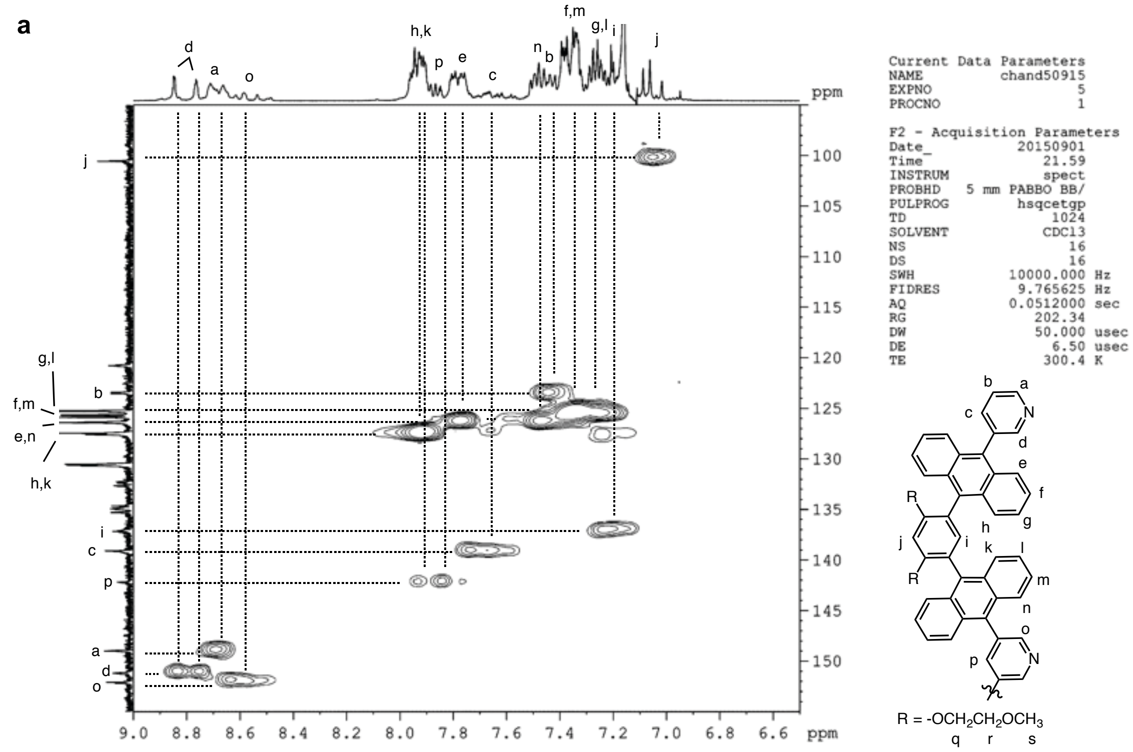

b

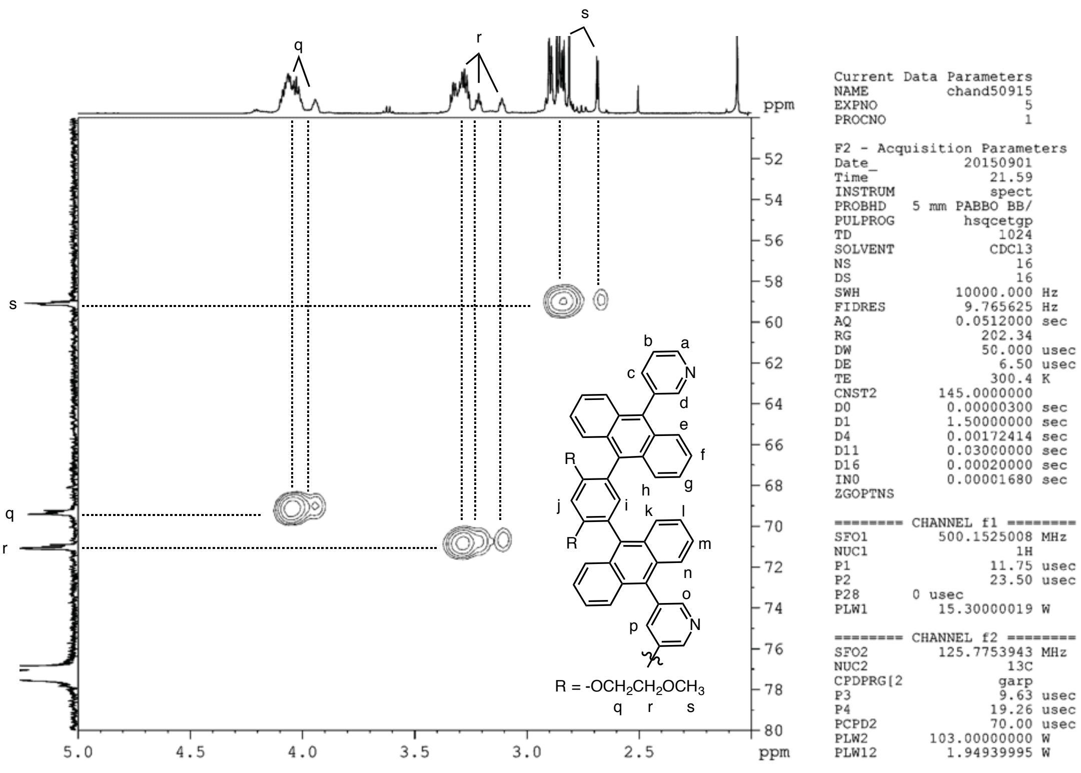

Supplementary Figure 9 | HSQC spectra (500 MHz, $CDCl_3$, room temperature) of **1a**. (a) The aromatic and (b) aliphatic regions.

a

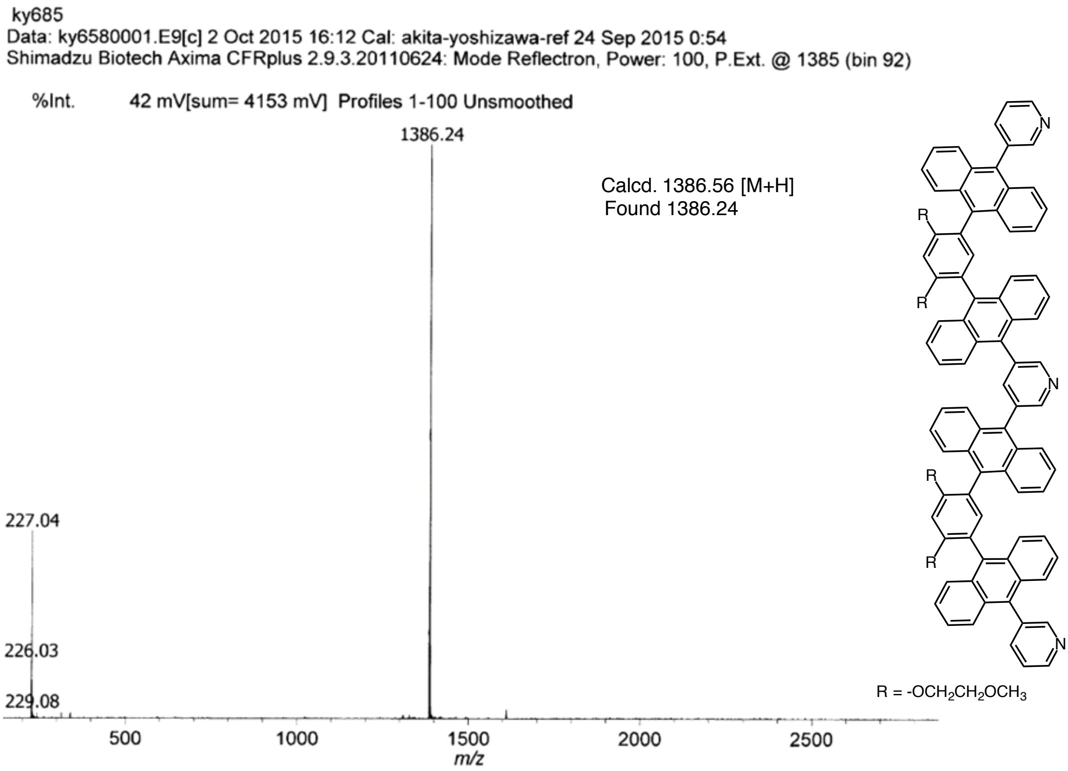

b

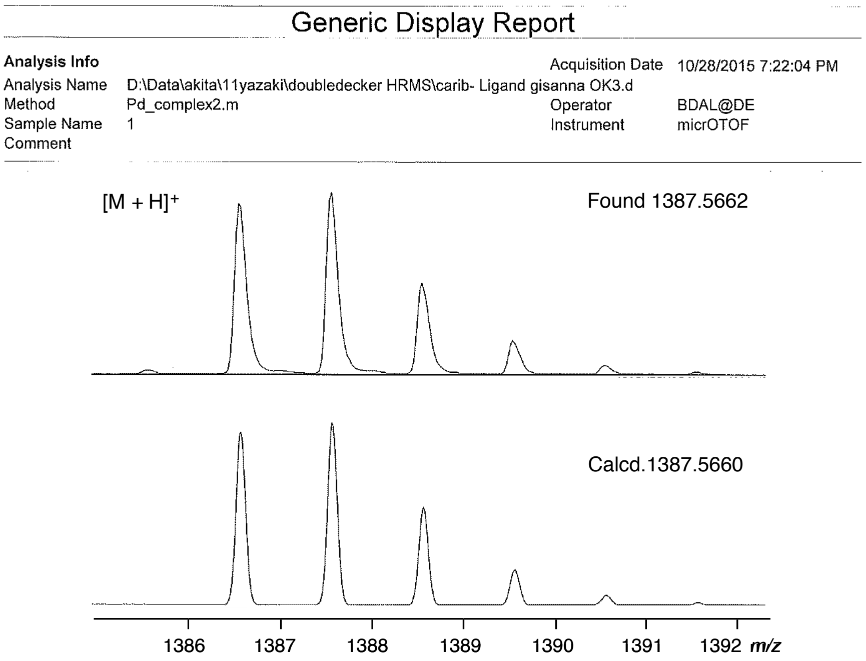

Supplementary Figure 10 | (a) MALDI-TOF MS (dithranol) and (b) HR MS (ESI) spectra of **1a**.

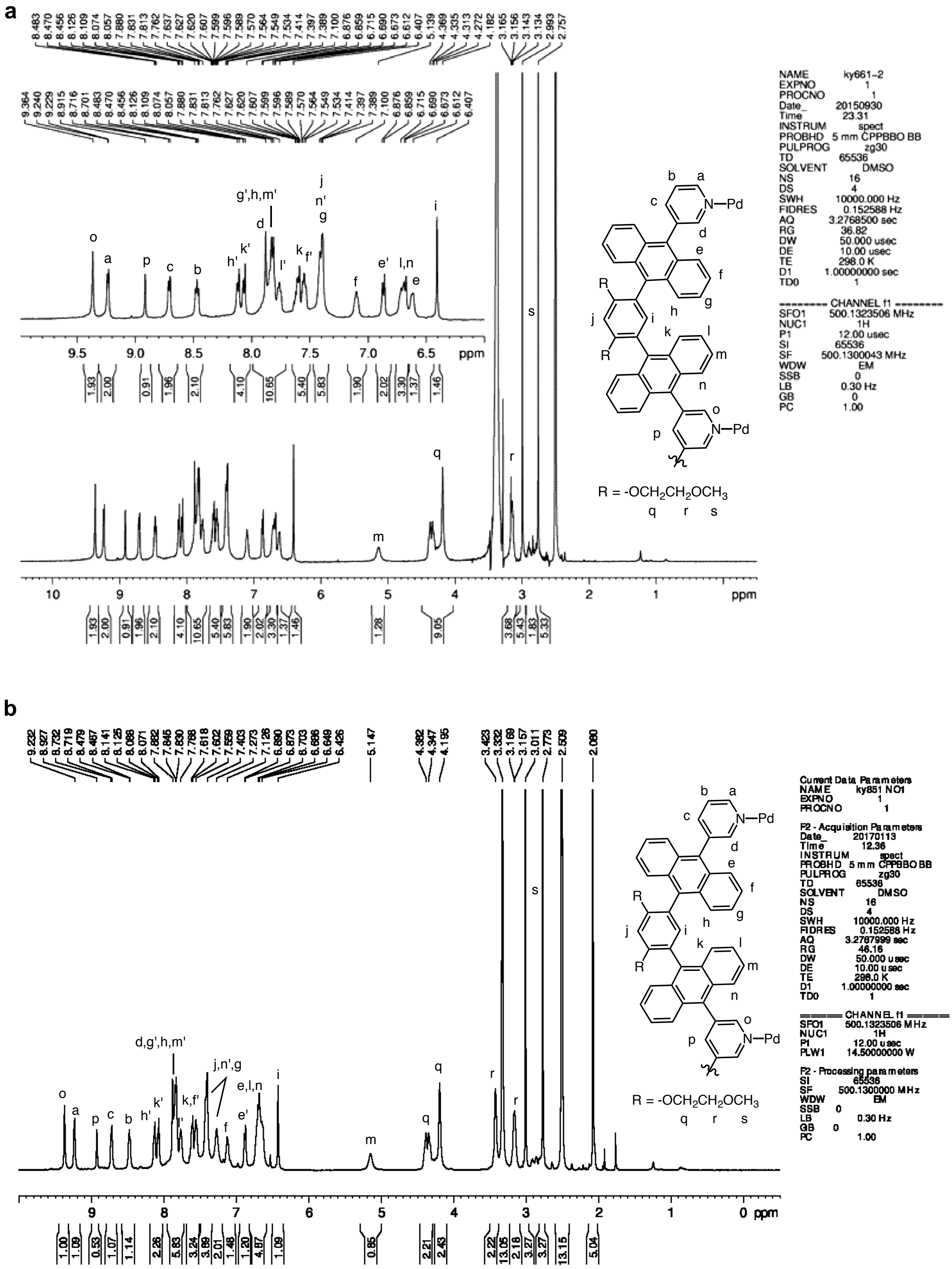

Supplementary Figure 11 | ^{1}H NMR spectra (500 MHz, DMSO-d_6, room temperature) of (a) **2a** and (b) **2a’**.

a

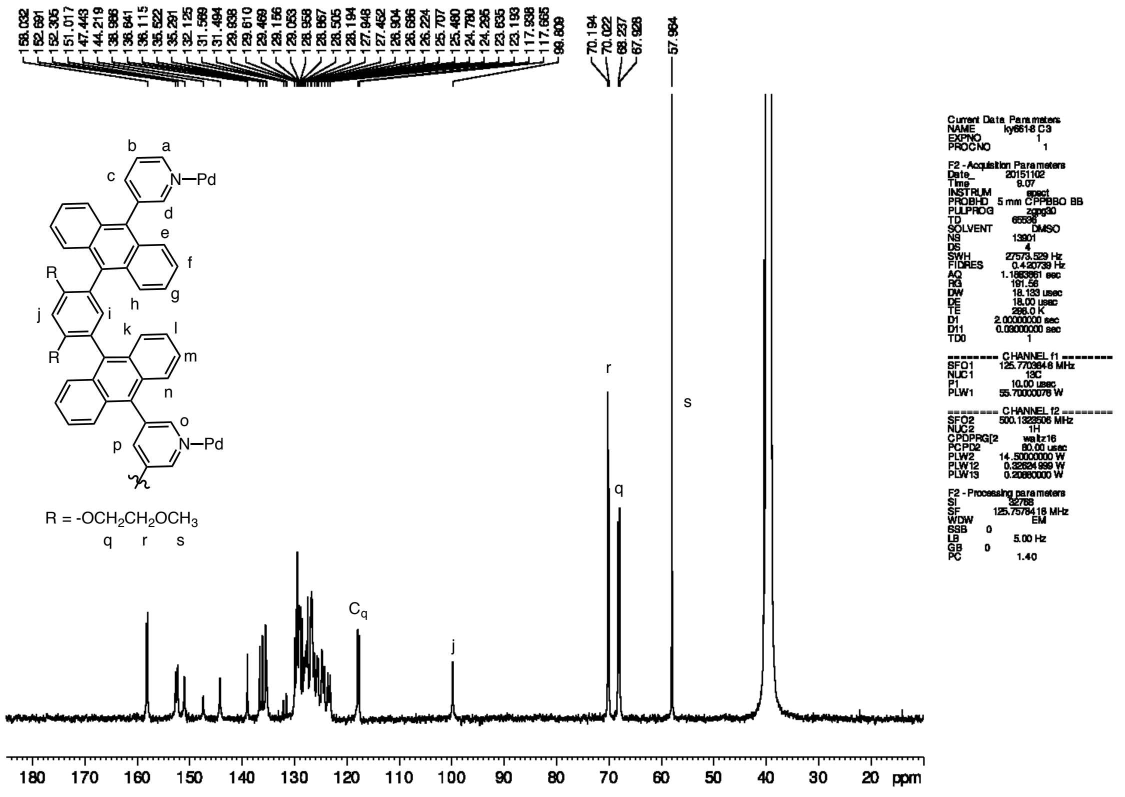

b

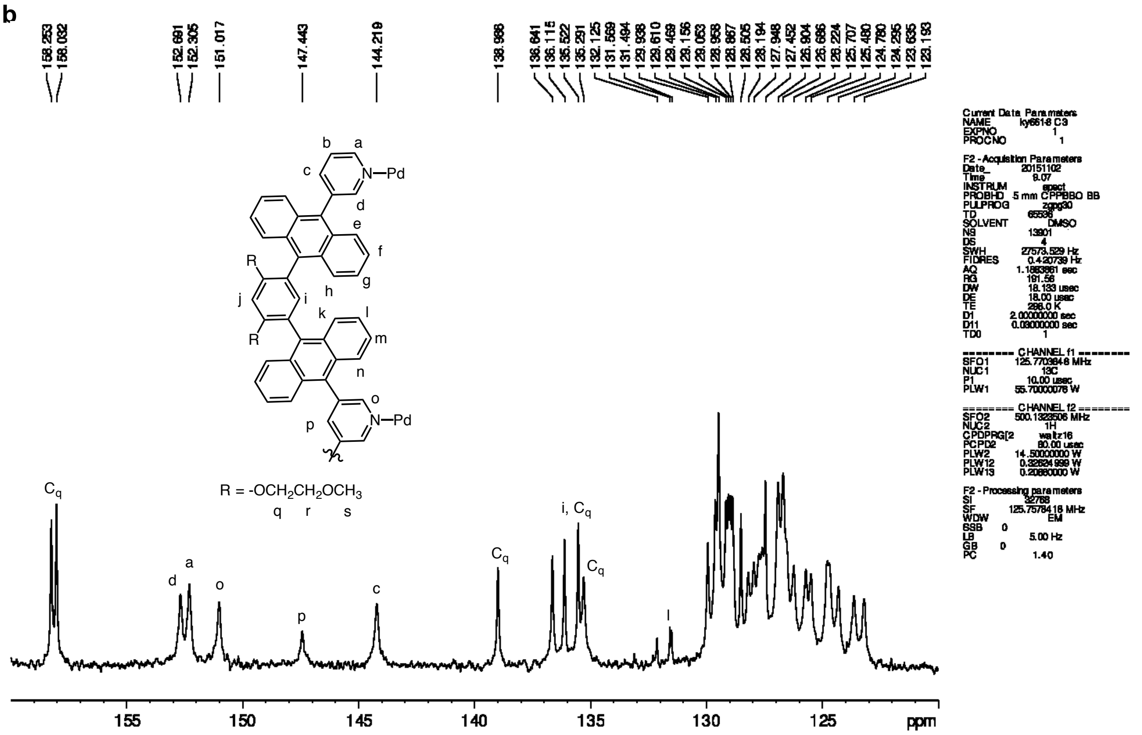

Supplementary Figure 12 | **^{13}C NMR spectra (125 MHz, DMSO-d_6, room temperature) of **2a. (a) The aromatic and aliphatic and (b) aromatic regions.

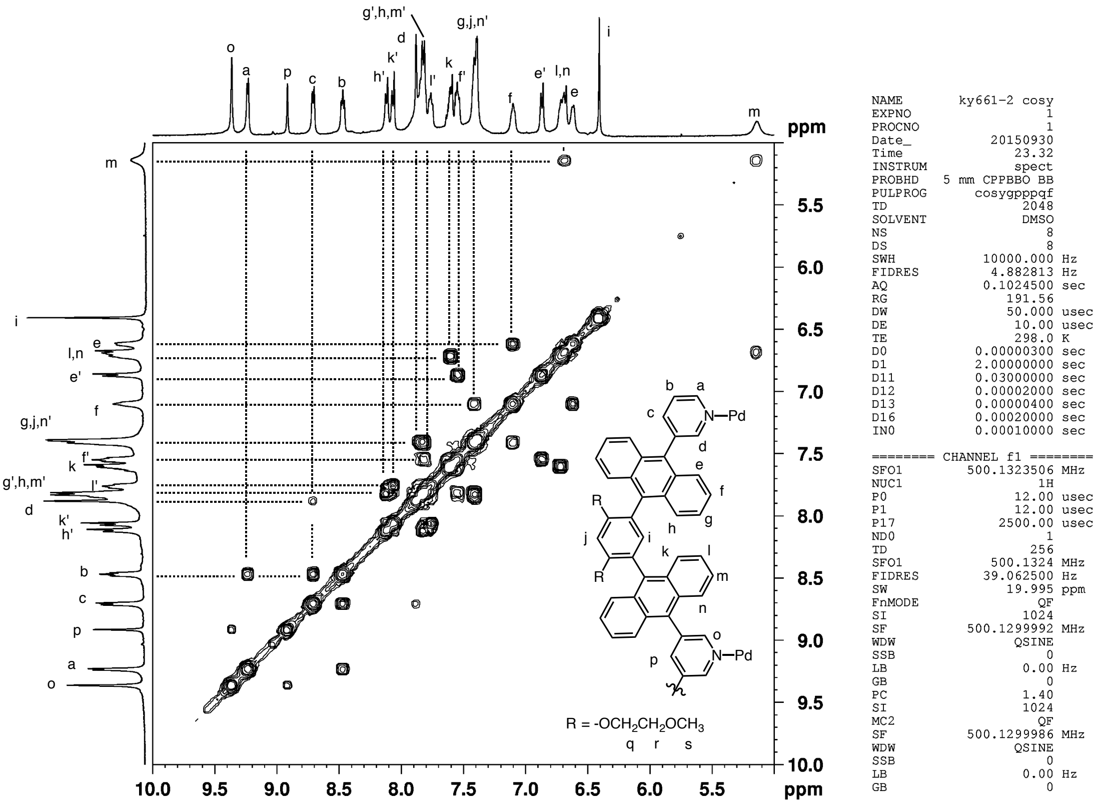

Supplementary Figure 13 | HH COSY spectrum (500 MHz, DMSO-d_6, room temperature) of 2a.

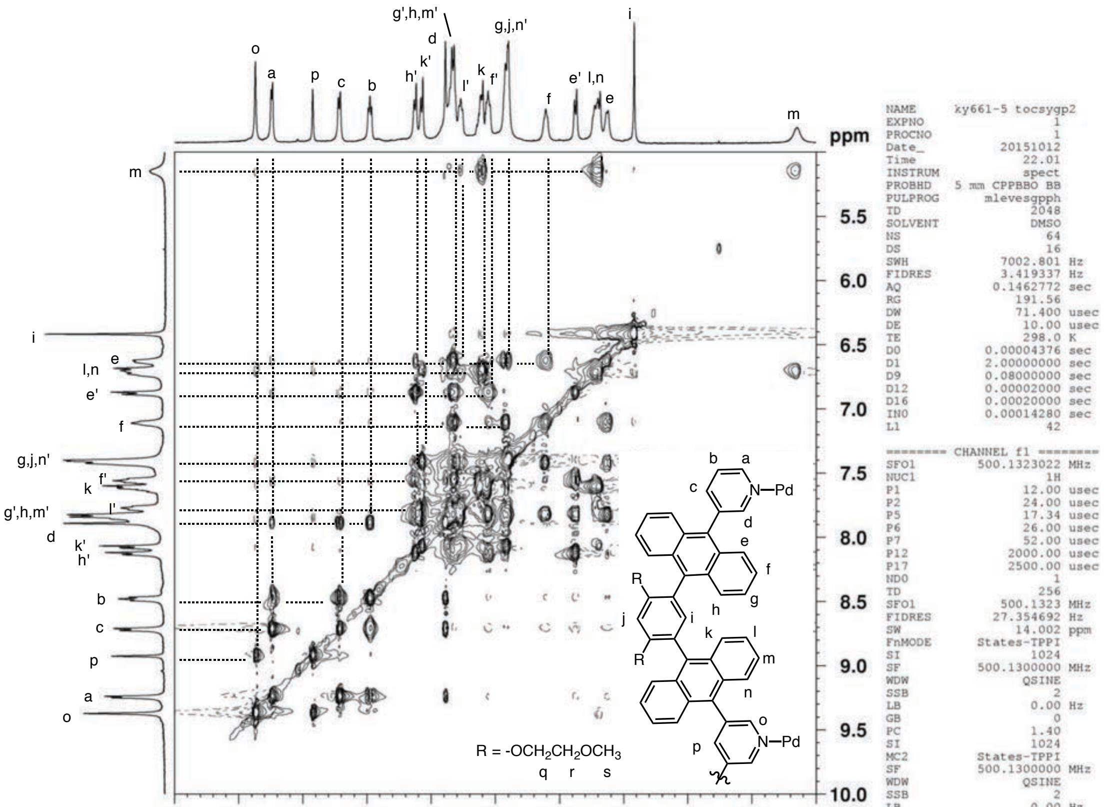

Supplementary Figure 14 | HH TOCSY spectrum (500 MHz, DMSO-d_6, room temperature) of 2a.

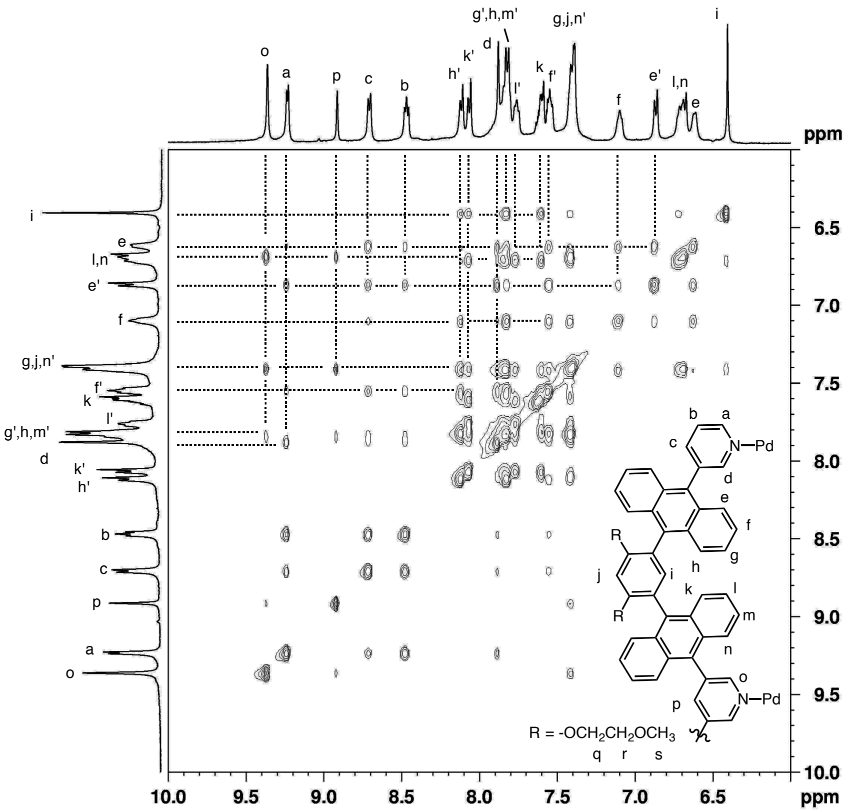


```
NAME            ky661-2 noesy2
EXPNO                        1
PROCNO                       1
Date_                 20151001
Time                     10.18
INSTRUM                  spect
PROBHD       5 mm CPPBBO BB
PULPROG           noesygpphpp
TD                        2048
SOLVENT                   DMSO
NS                           8
DS                          16
SWH                   7002.801 Hz
FIDRES                3.419337 Hz
AQ                   0.1462772 sec
RG                       61.52
DW                      71.400 usec
DE                       10.00 usec
TE                       298.0 K
D0                  0.00005612 sec
D1                  2.00000000 sec
D8                  0.40000001 sec
D11                 0.03000000 sec
D12                 0.00002000 sec
D16                 0.00020000 sec
IN0                 0.00014280 sec

======== CHANNEL f1 ========
SFO1               500.1323506 MHz
NUC1                        1H
P1                       12.00 usec
P2                       24.00 usec
P17                    2500.00 usec
ND0                          1
TD                         256
SFO1                  500.1324 MHz
FIDRES               27.354692 Hz
SW                      14.002 ppm
FnMODE             States-TPPI
SI                        1024
SF                 500.1300000 MHz
WDW                      QSINE
SSB                          2
LB                        0.00 Hz
GB                           0
PC                        1.00
SI                        1024
MC2                States-TPPI
SF                 500.1300000 MHz
WDW                      QSINE
SSB                          2
LB                        0.00 Hz
GB                           0
```

Supplementary Figure 15 | NOESY spectrum (500 MHz, DMSO-d_6, room temperature) of **2a**.

a

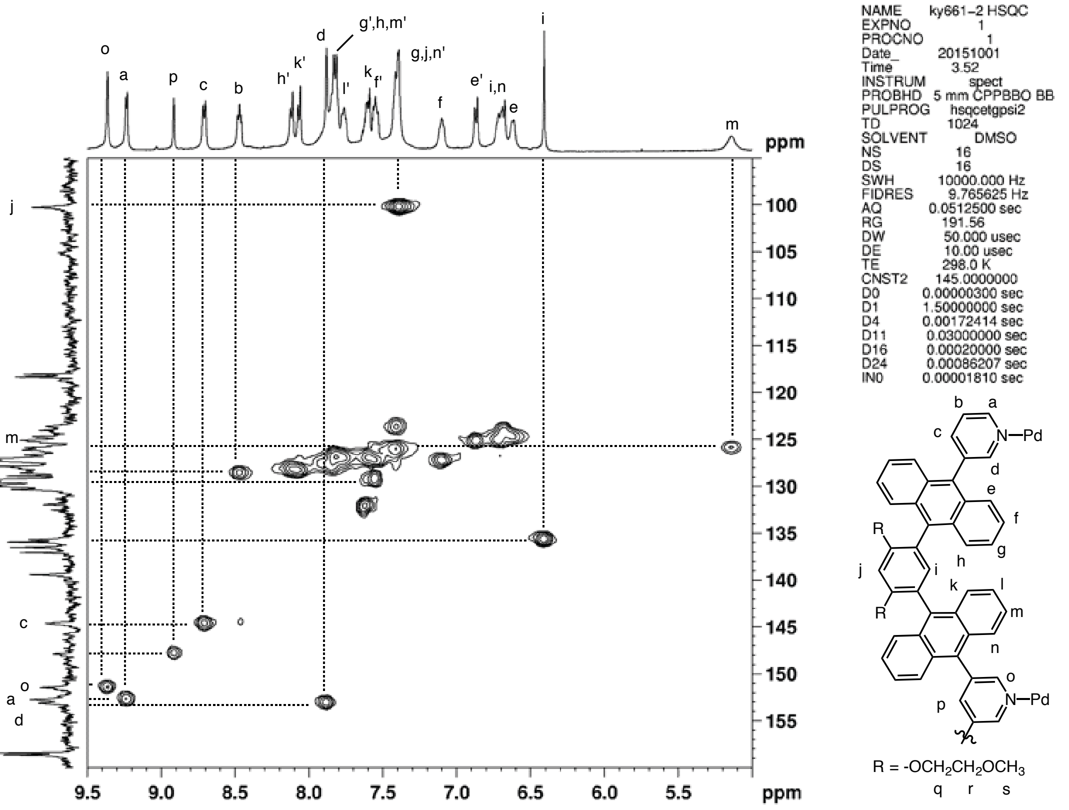

NAME ky661-2 HSQC
EXPNO 1
PROCNO 1
Date_ 20151001
Time 3.52
INSTRUM spect
PROBHD 5 mm CPPBBO BB
PULPROG hsqcetgpsi2
TD 1024
SOLVENT DMSO
NS 16
DS 16
SWH 10000.000 Hz
FIDRES 9.765625 Hz
AQ 0.0512500 sec
RG 191.56
DW 50.000 usec
DE 10.00 usec
TE 298.0 K
CNST2 145.0000000
D0 0.00000300 sec
D1 1.50000000 sec
D4 0.00172414 sec
D11 0.03000000 sec
D16 0.00020000 sec
D24 0.00086207 sec
IN0 0.00001810 sec
R = -OCH2CH2OCH3
q r s
ppm

b

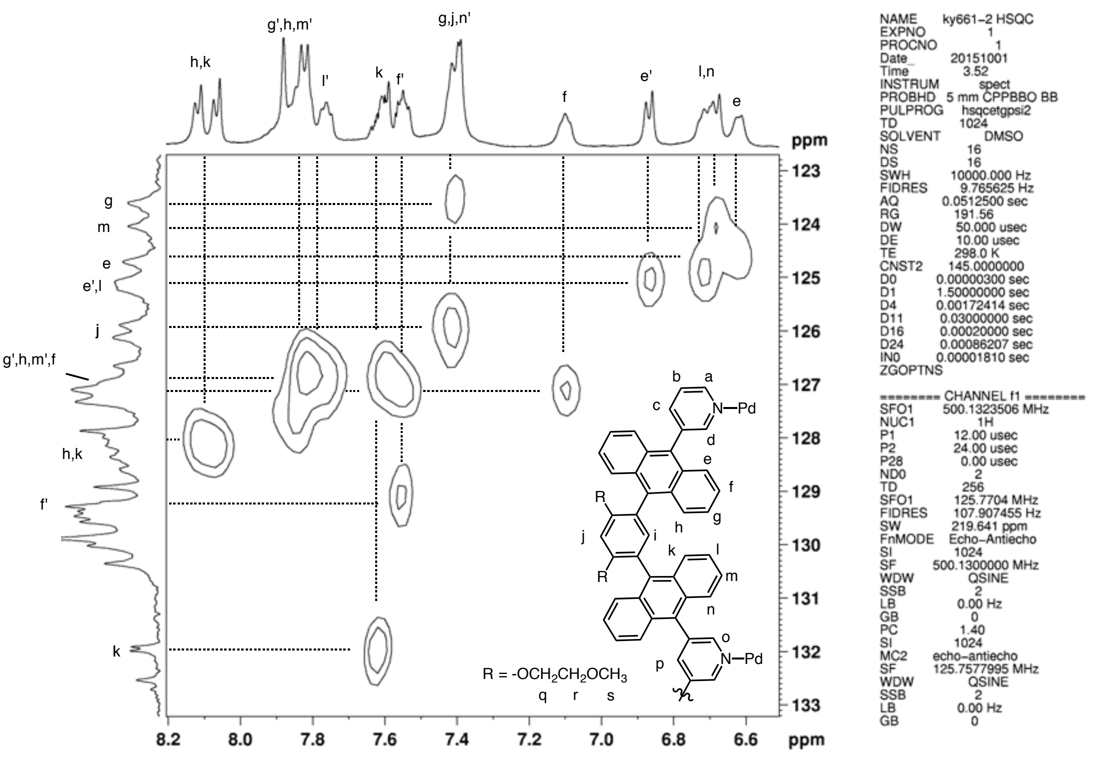

NAME ky661-2 HSQC
EXPNO 1
PROCNO 1
Date_ 20151001
Time 3.52
INSTRUM spect
PROBHD 5 mm CPPBBO BB
PULPROG hsqcetgpsi2
TD 1024
SOLVENT DMSO
NS 16
DS 16
SWH 10000.000 Hz
FIDRES 9.765625 Hz
AQ 0.0512500 sec
RG 191.56
DW 50.000 usec
DE 10.00 usec
TE 298.0 K
CNST2 145.0000000
D0 0.00000300 sec
D1 1.50000000 sec
D4 0.00172414 sec
D11 0.03000000 sec
D16 0.00020000 sec
D24 0.00086207 sec
IN0 0.00001810 sec
ZGOPTNS
======== CHANNEL f1 ========
SFO1 500.1323506 MHz
NUC1 1H
P1 12.00 usec
P2 24.00 usec
P28 0.00 usec
ND0 2
TD 256
SFO1 125.7704 MHz
FIDRES 107.907455 Hz
SW 219.641 ppm
FnMODE Echo-Antiecho
SI 1024
SF 500.1300000 MHz
WDW QSINE
SSB 2
LB 0.00 Hz
GB 0
PC 1.40
SI 1024
MC2 echo-antiecho
SF 125.7577995 MHz
WDW QSINE
SSB 2
LB 0.00 Hz
GB 0
R = -OCH2CH2OCH3
q r s
ppm

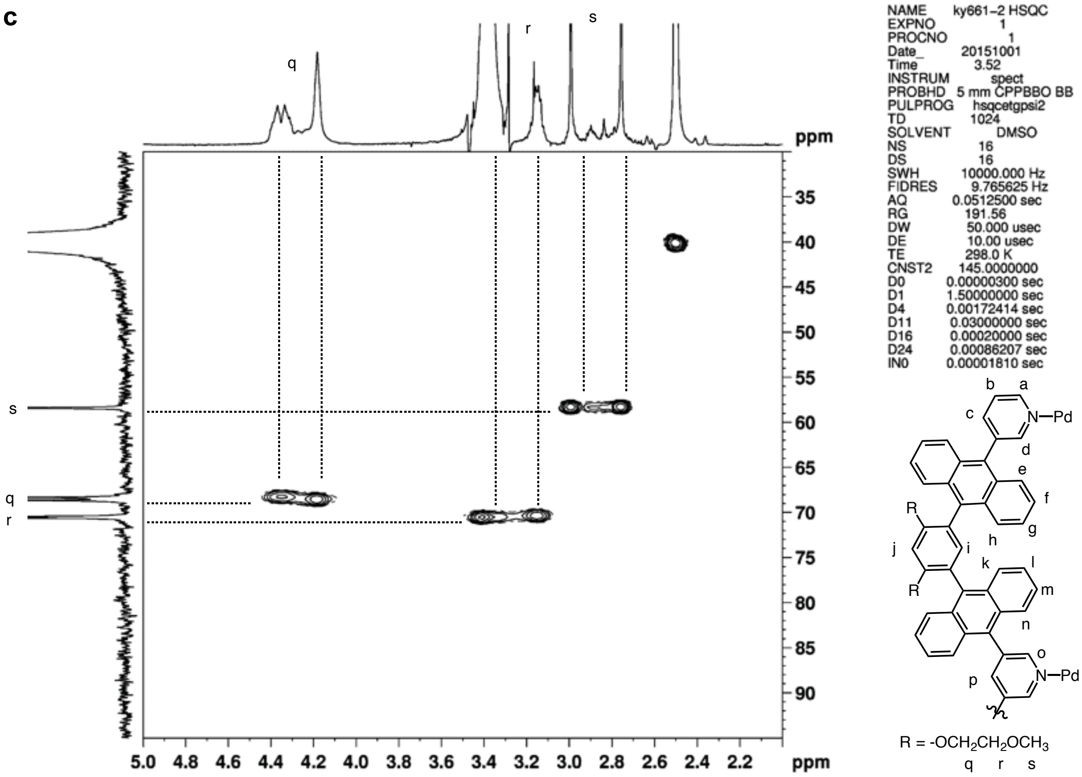

Supplementary Figure 16 | HSQC spectra (500 MHz, DMSO-d_6, room temperature) of **2a**. (a,b) The aromatic and (c) aliphatic regions.

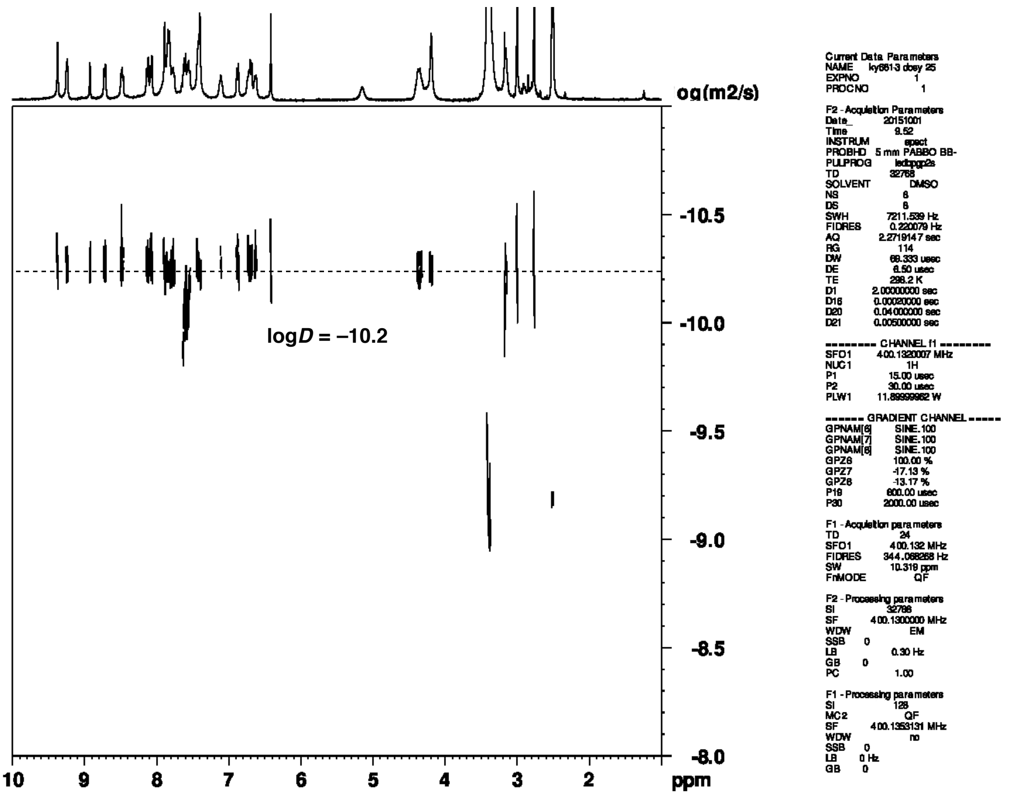

Supplementary Figure 17 | DOSY NMR (500 MHz, DMSO-d_6, 298 K) of **2a**.

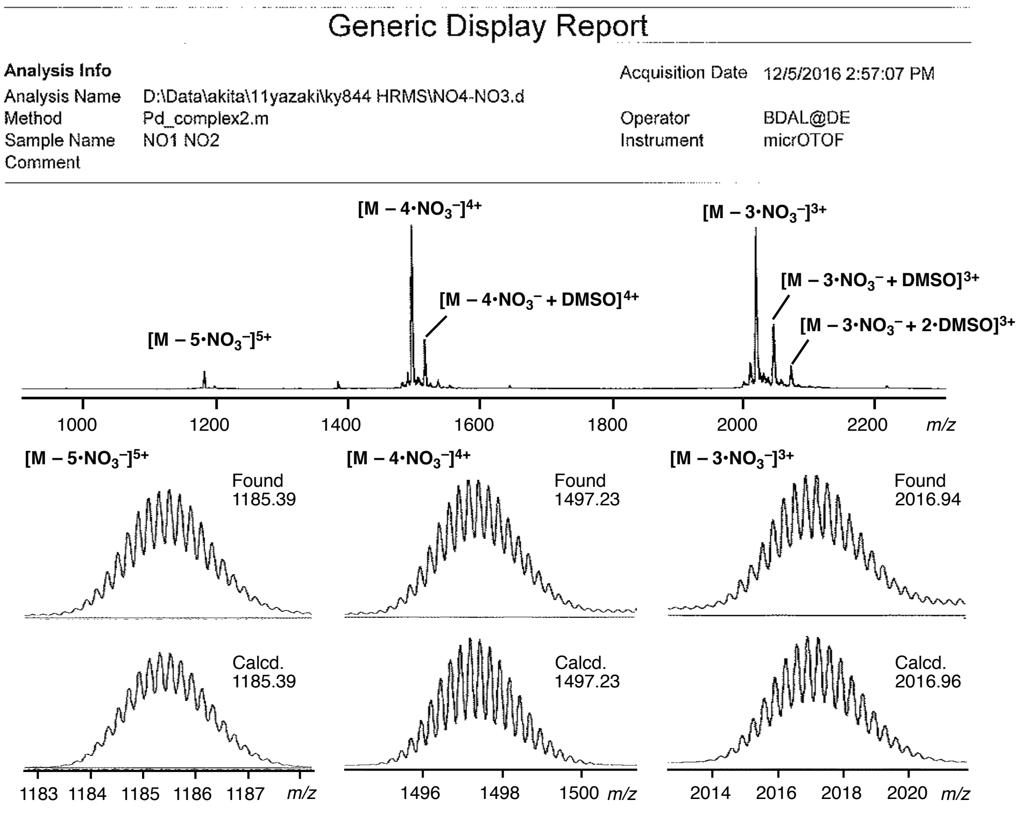

Supplementary Figure 18 | ESI-TOF MS spectrum (CH_3OH) of **2a**.

a

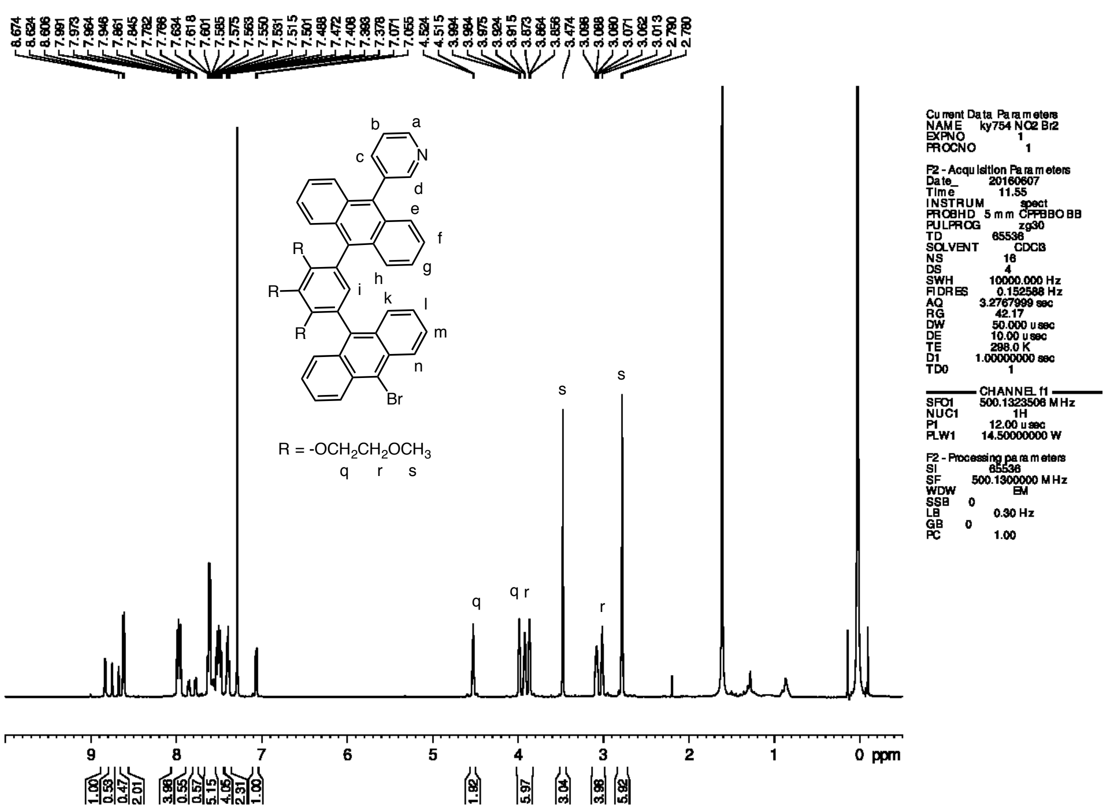

b

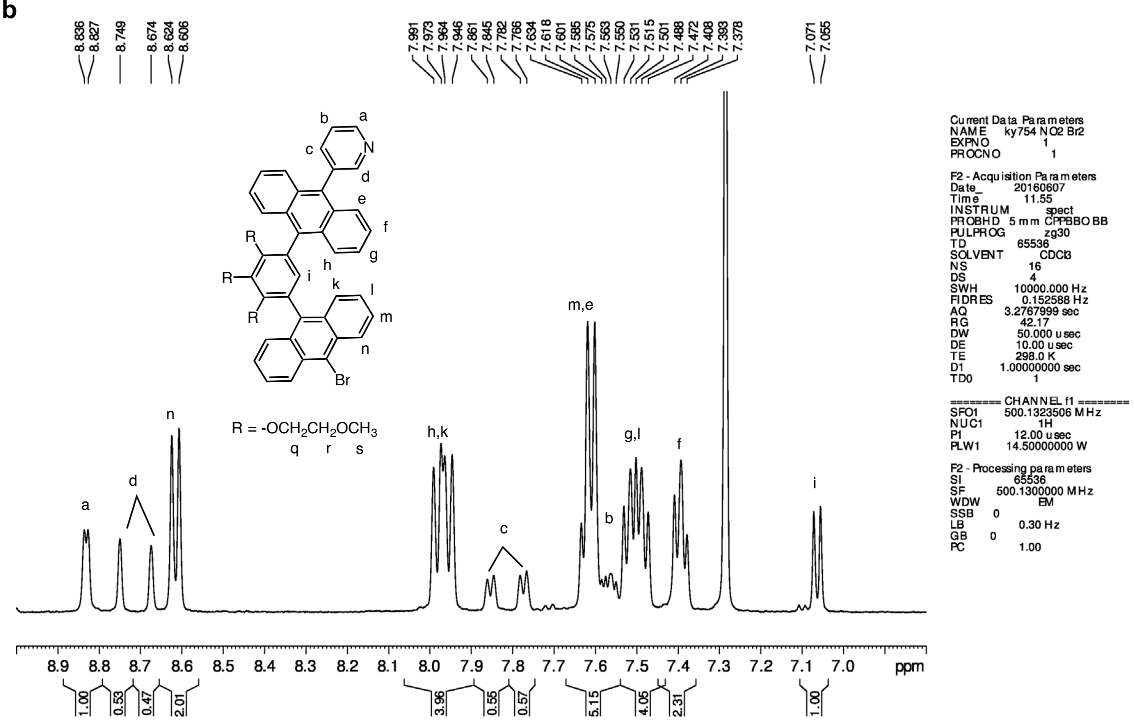

Supplementary Figure 19 | **^{1}H NMR spectra (500 MHz, $CDCl_3$, room temperature) of **6b. (a) The aromatic and aliphatic and (b) aromatic regions.

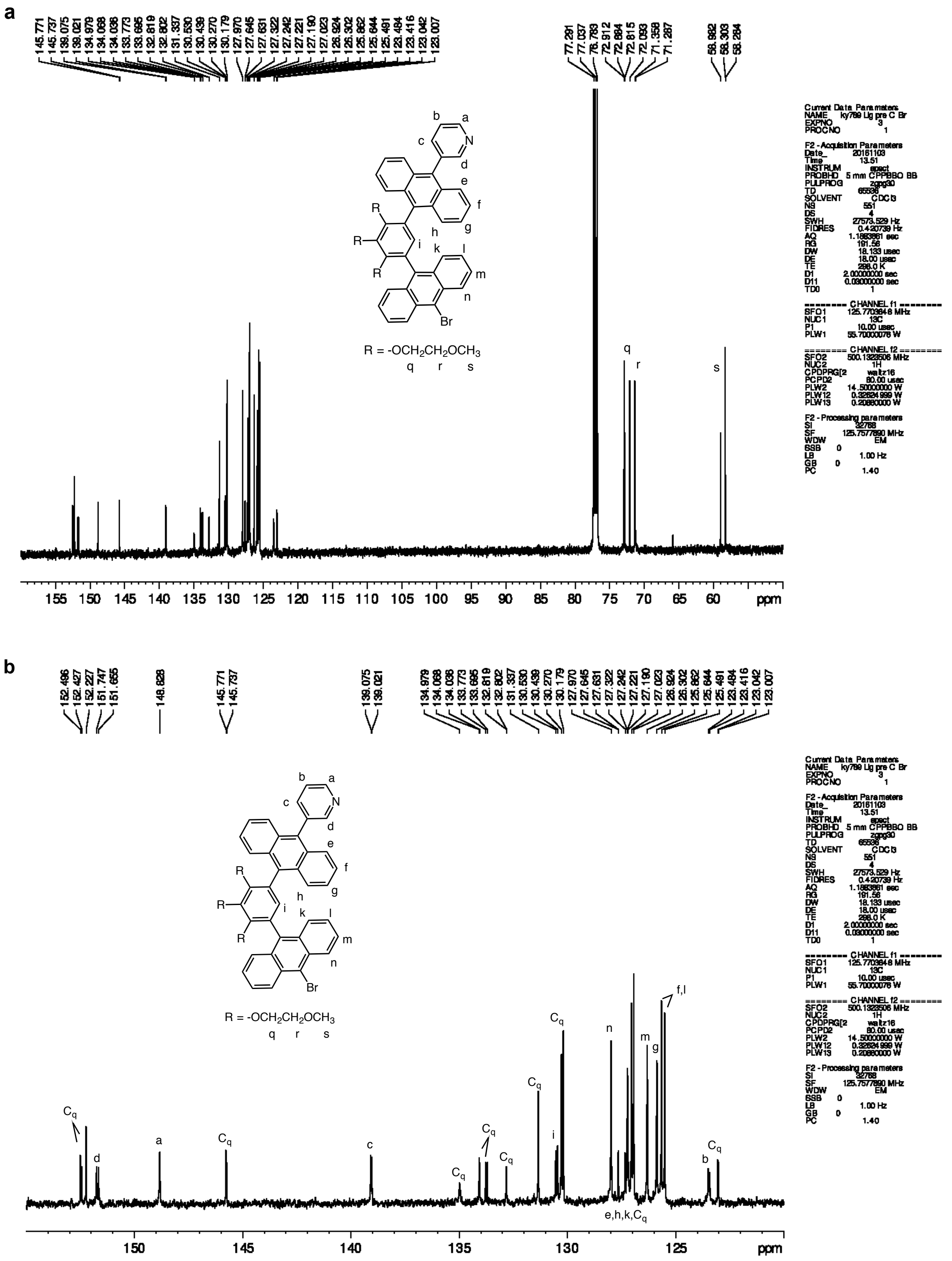

Supplementary Figure 20 | ^{13}C NMR spectra (125 MHz, $CDCl_3$, room temperature) of **6b**. (a) The aromatic and aliphatic and (b) aromatic regions.

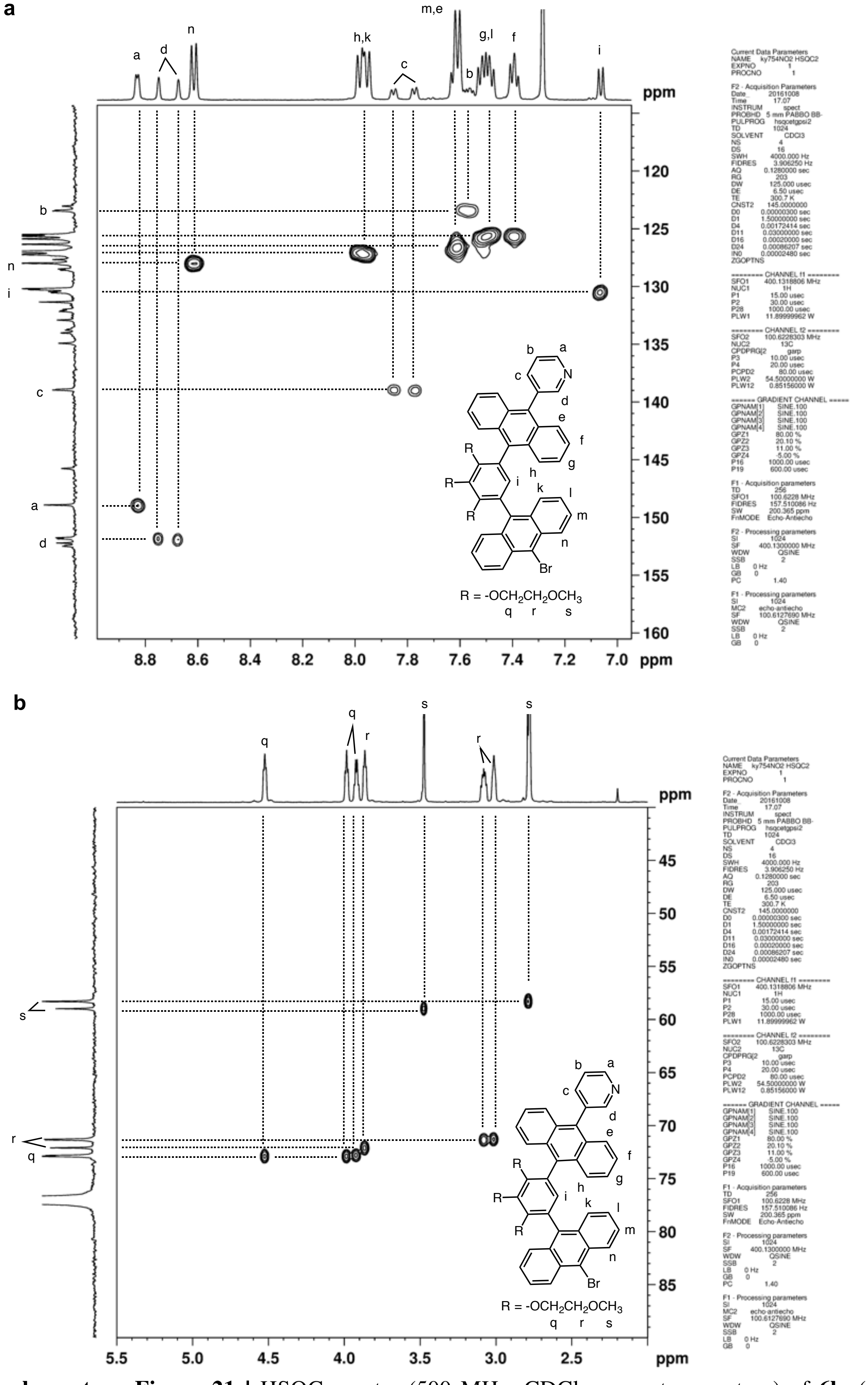

Supplementary Figure 21 | HSQC spectra (500 MHz, $CDCl_3$, room temperature) of **6b**. (a) The aromatic and (b) aliphatic regions.

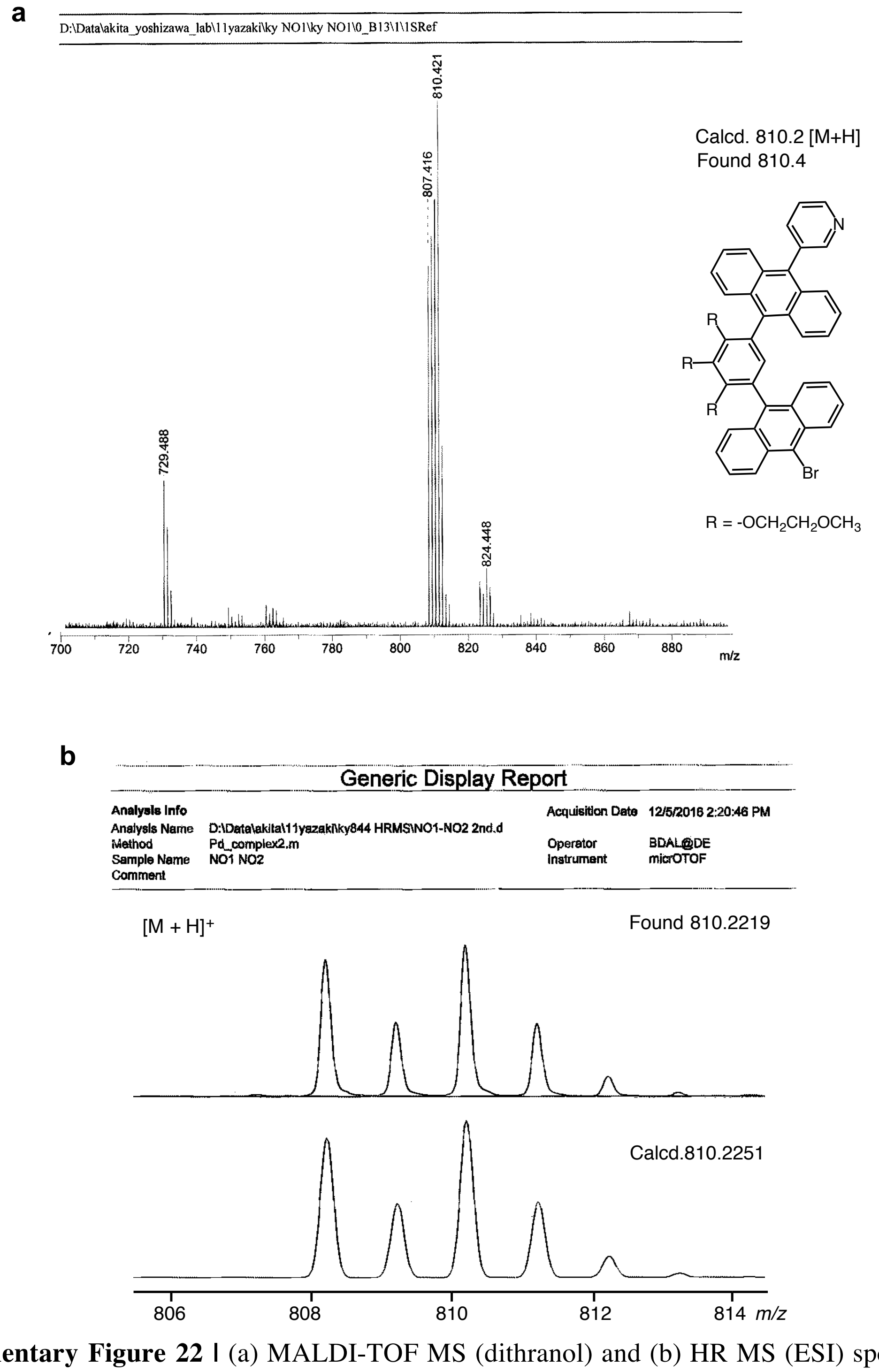

Supplementary Figure 22 | (a) MALDI-TOF MS (dithranol) and (b) HR MS (ESI) spectra of 6b.

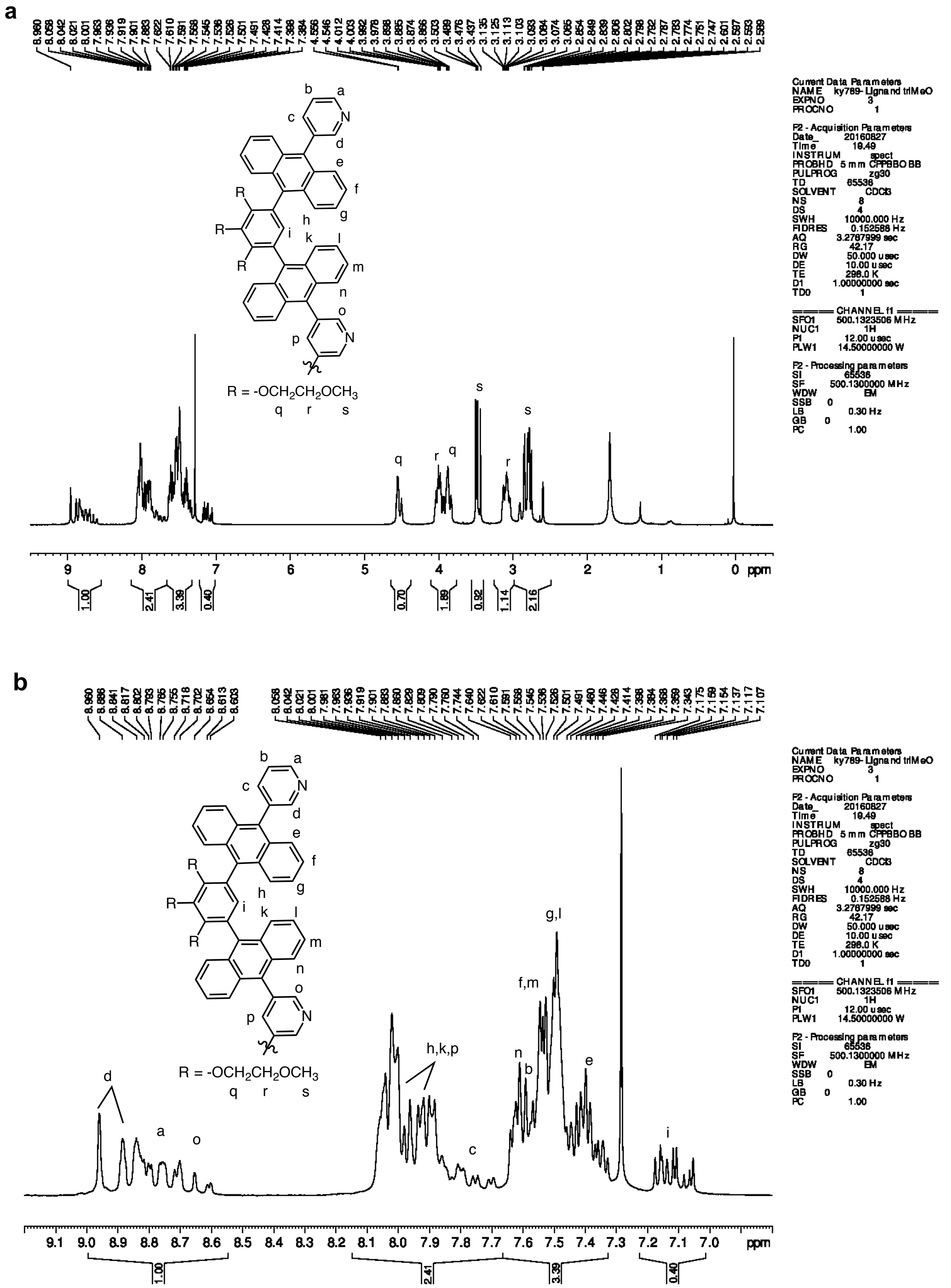

Supplementary Figure 23 | **^{1}H NMR spectra (500 MHz, $CDCl_3$, room temperature) of **1b. (a) The aromatic and aliphatic and (b) aromatic regions.

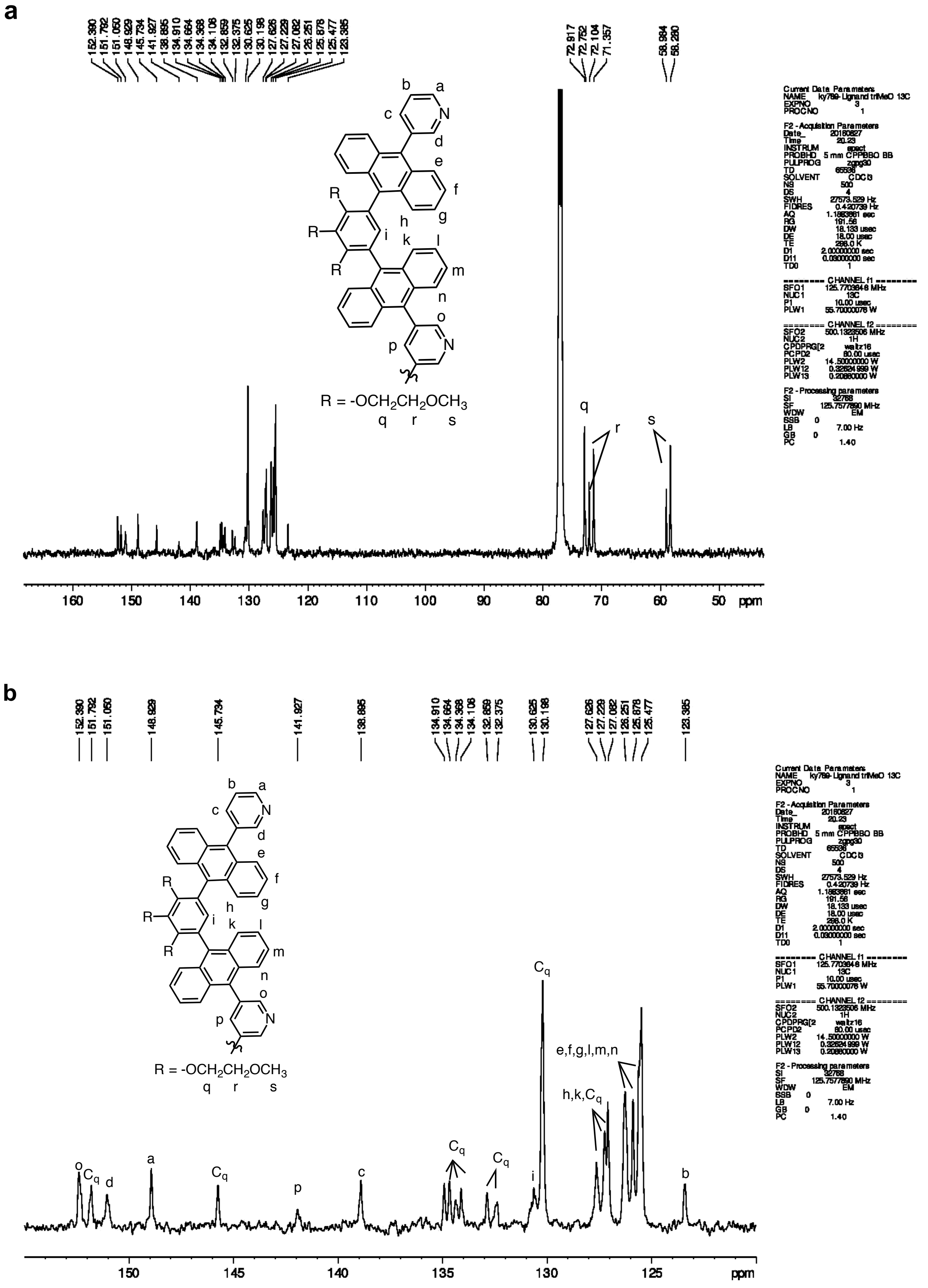

Supplementary Figure 24 | ^{13}C NMR spectra (125 MHz, $CDCl_3$, room temperature) of **1b**. (a) The aromatic and aliphatic and (b) aromatic regions.

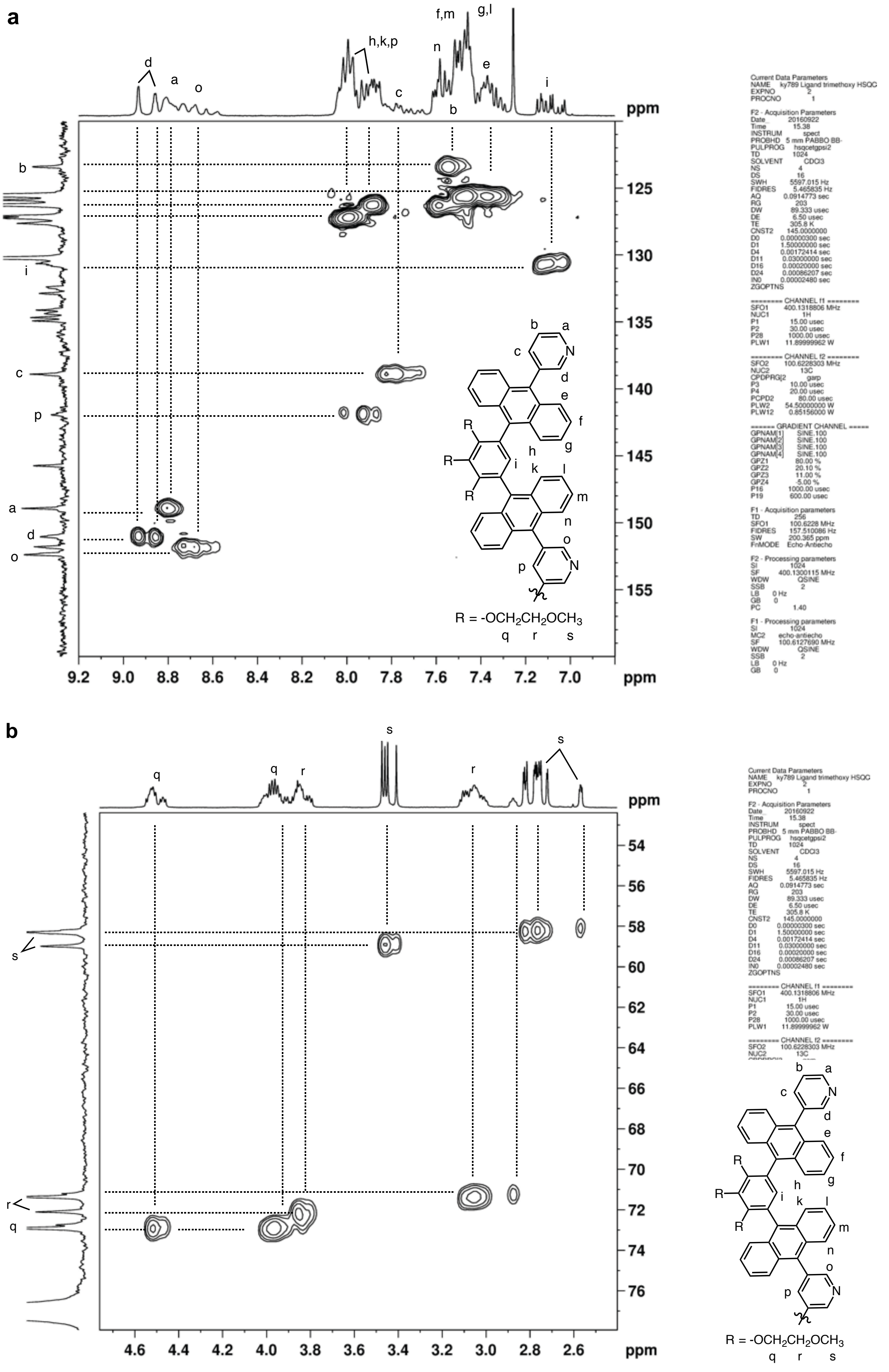

Supplementary Figure 25 | HSQC spectra (500 MHz, CDCl$_3$, room temperature) of 1b. (a) The aromatic and (b) aliphatic regions.

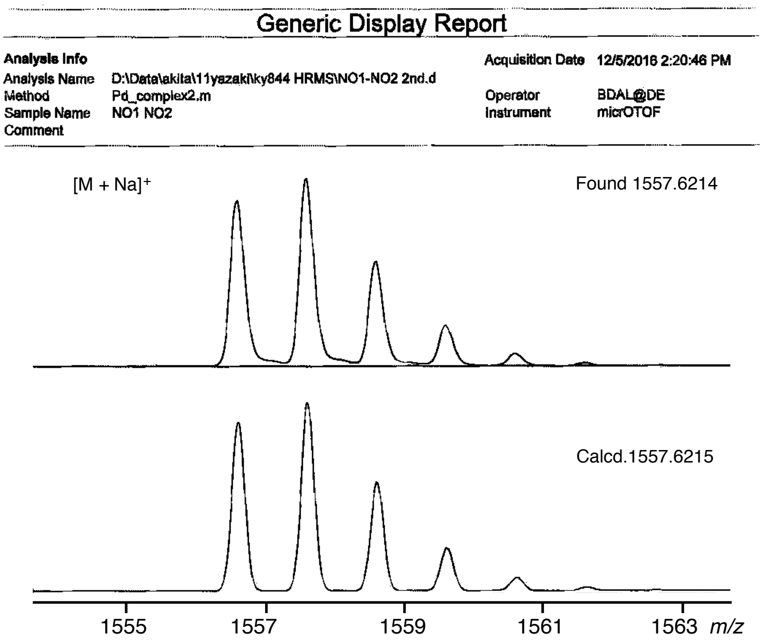

Supplementary Figure 26 | HR MS spectrum (ESI) of **1b**.

a

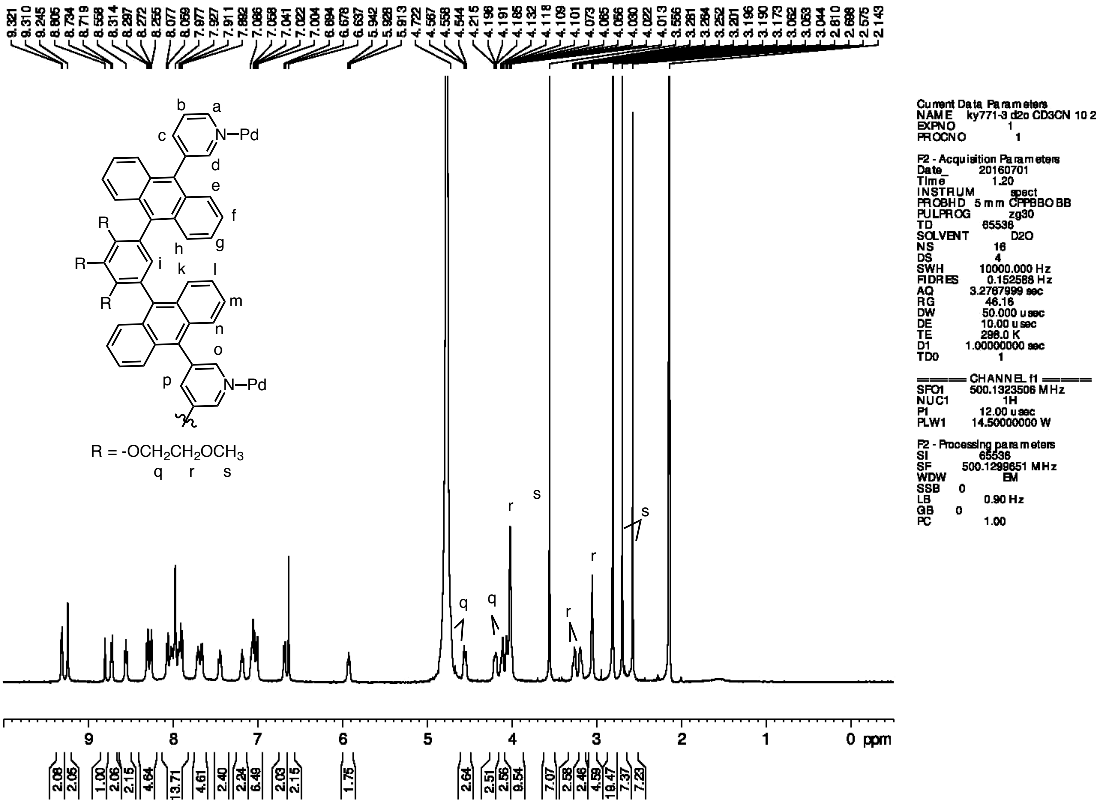

b
a
c
N–Pd
d
e
f
g
h
i
k
l
m
n
o
p
R
R
R
N–Pd
R = -OCH2CH2OCH3
q r s
q
q
r
s
r
r
s
9
8
7
6
5
4
3
2
1
0 ppm
Current Data Parameters
NAME ky771-3 d2o CD3CN 10 2
EXPNO 1
PROCNO 1
F2 - Acquisition Parameters
Date_ 20160701
Time 1.20
INSTRUM spect
PROBHD 5 mm CPPBBO BB
PULPROG zg30
TD 65536
SOLVENT D2O
NS 16
DS 4
SWH 10000.000 Hz
FIDRES 0.152588 Hz
AQ 3.2767999 sec
RG 46.16
DW 50.000 usec
DE 10.00 usec
TE 298.0 K
D1 1.00000000 sec
TD0 1
CHANNEL f1
SFO1 500.1323506 MHz
NUC1 1H
P1 12.00 usec
PLW1 14.50000000 W
F2 - Processing parameters
SI 65536
SF 500.1299651 MHz
WDW EM
SSB 0
LB 0.90 Hz
GB 0
PC 1.00

b

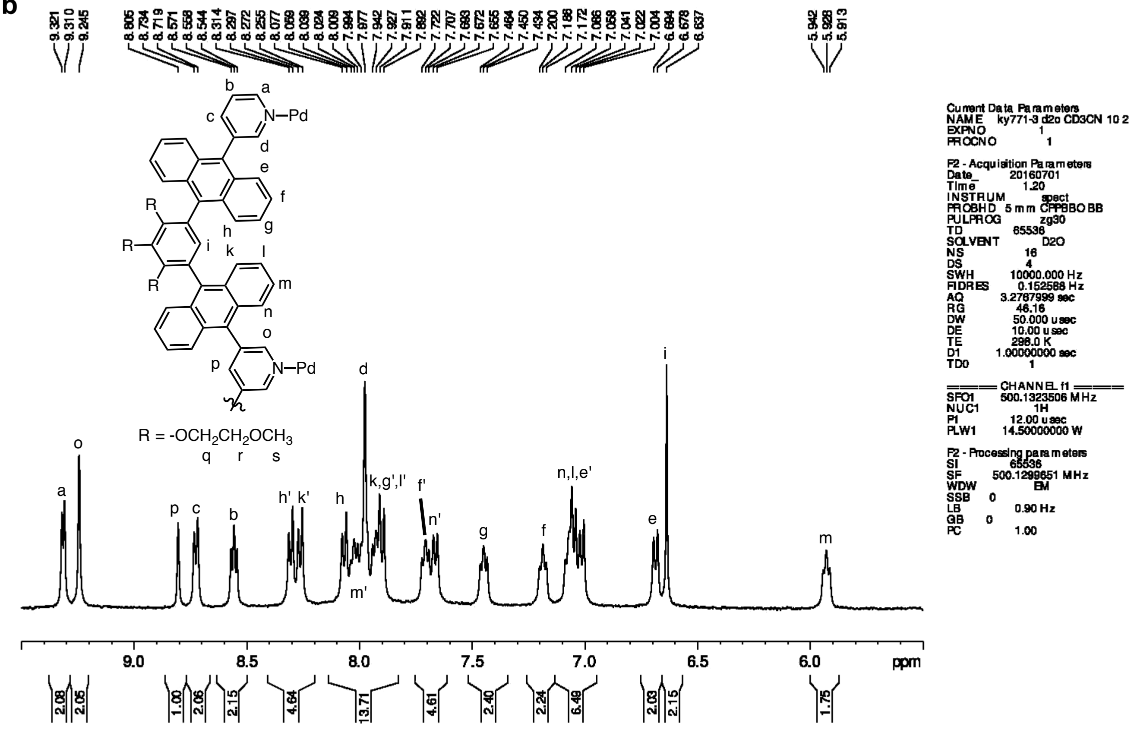

b
a
c
N–Pd
d
e
f
g
h
i
k
l
m
n
o
p
R
R
R
N–Pd
R = -OCH2CH2OCH3
q r s
a
o
p
c
b
h'
k'
h
d
k,g',l'
f'
n'
m'
g
f
n,l,e'
e
i
m
9.0
8.5
8.0
7.5
7.0
6.5
6.0
ppm
Current Data Parameters
NAME ky771-3 d2o CD3CN 10 2
EXPNO 1
PROCNO 1
F2 - Acquisition Parameters
Date_ 20160701
Time 1.20
INSTRUM spect
PROBHD 5 mm CPPBBO BB
PULPROG zg30
TD 65536
SOLVENT D2O
NS 16
DS 4
SWH 10000.000 Hz
FIDRES 0.152588 Hz
AQ 3.2767999 sec
RG 46.16
DW 50.000 usec
DE 10.00 usec
TE 298.0 K
D1 1.00000000 sec
TD0 1
CHANNEL f1
SFO1 500.1323506 MHz
NUC1 1H
P1 12.00 usec
PLW1 14.50000000 W
F2 - Processing parameters
SI 65536
SF 500.1299651 MHz
WDW EM
SSB 0
LB 0.90 Hz
GB 0
PC 1.00

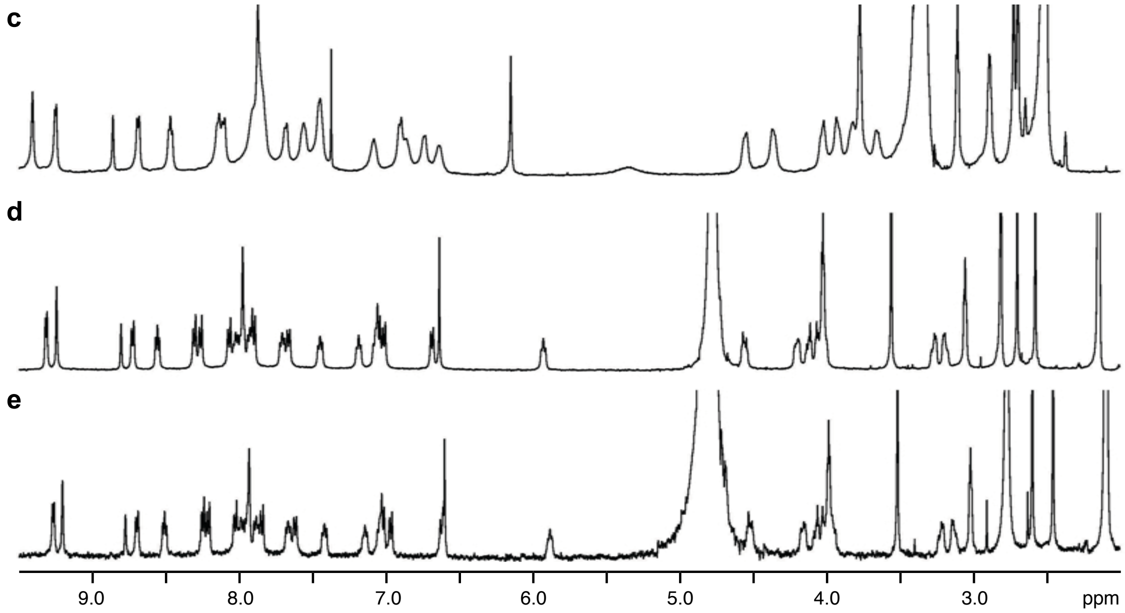

Supplementary Figure 27 | ^{1}H NMR spectra (500 MHz, D_2O:CD_3CN = 5:1, room temperature) of **2b**. (a) The aromatic and aliphatic and (b) aromatic regions. ^{1}H NMR spectra (500 MHz, room temperature) of **2b** in (c) DMSO-d_6, (d) D_2O:CD_3CN = 5:1, and (e) D_2O:CD_3CN = 100:1.

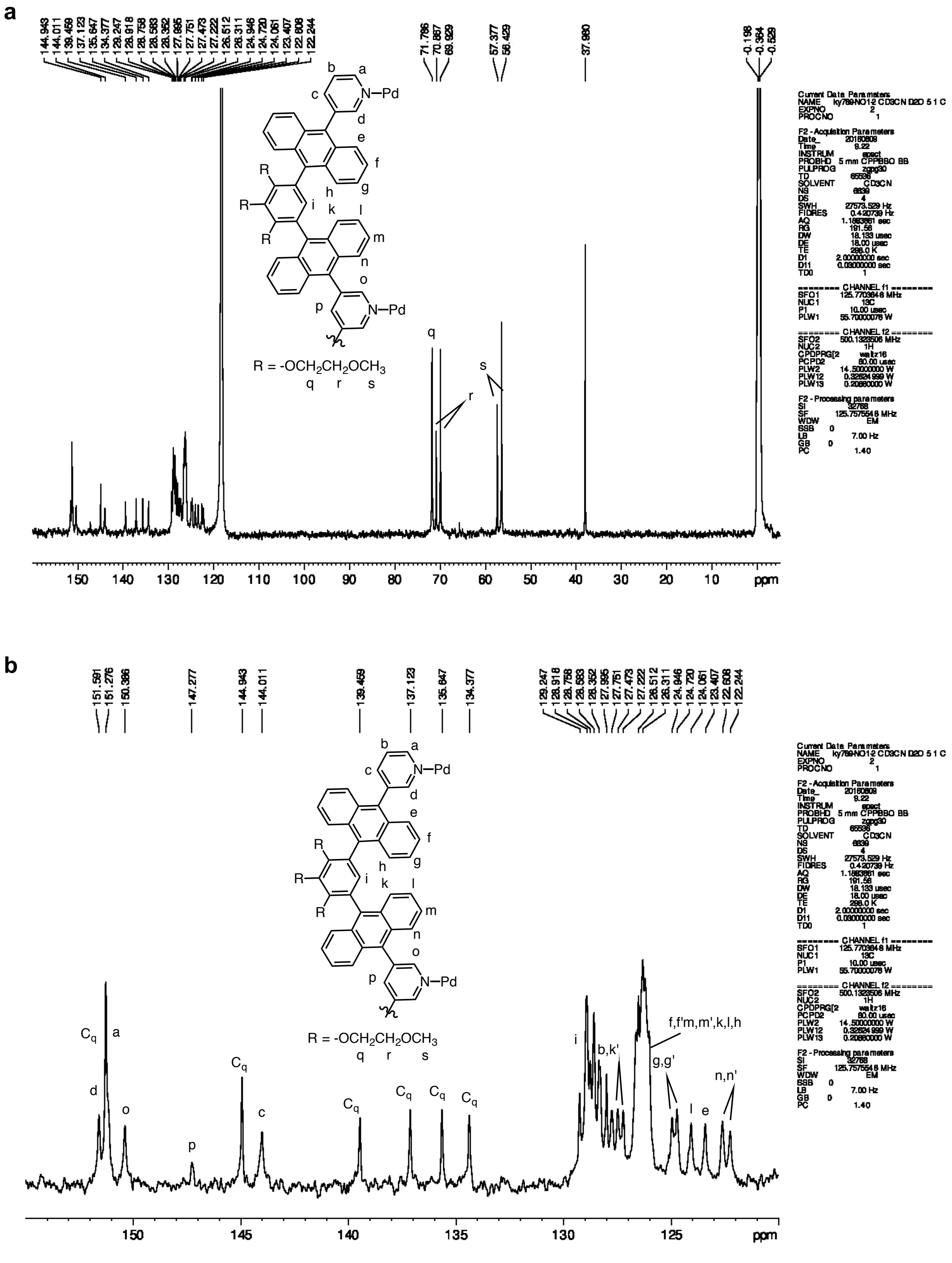

Supplementary Figure 28 | ^{13}C NMR spectra (125 MHz, D_2O:CD_3CN = 5:1, room temperature) of **2b**. (a) The aromatic and aliphatic and (b) aromatic regions.

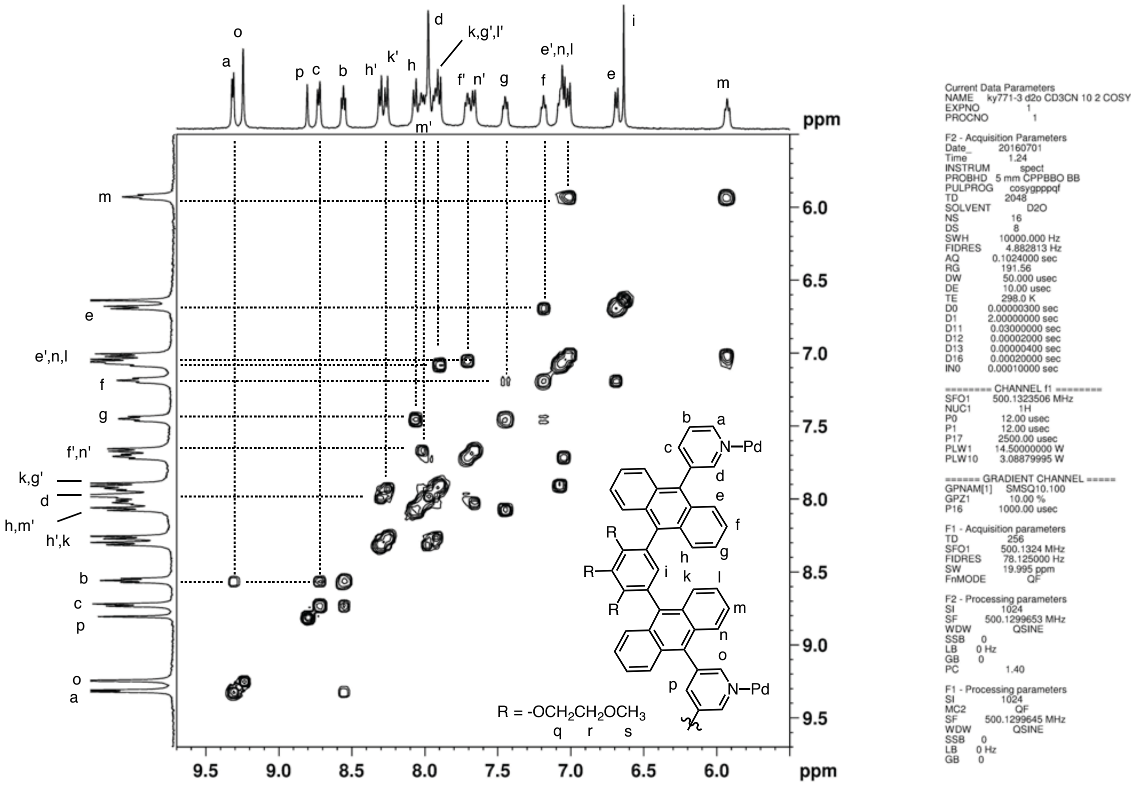

Supplementary Figure 29 | HH COSY spectrum (500 MHz, D_2O:CD_3CN = 5:1, room temperature) of 2b.

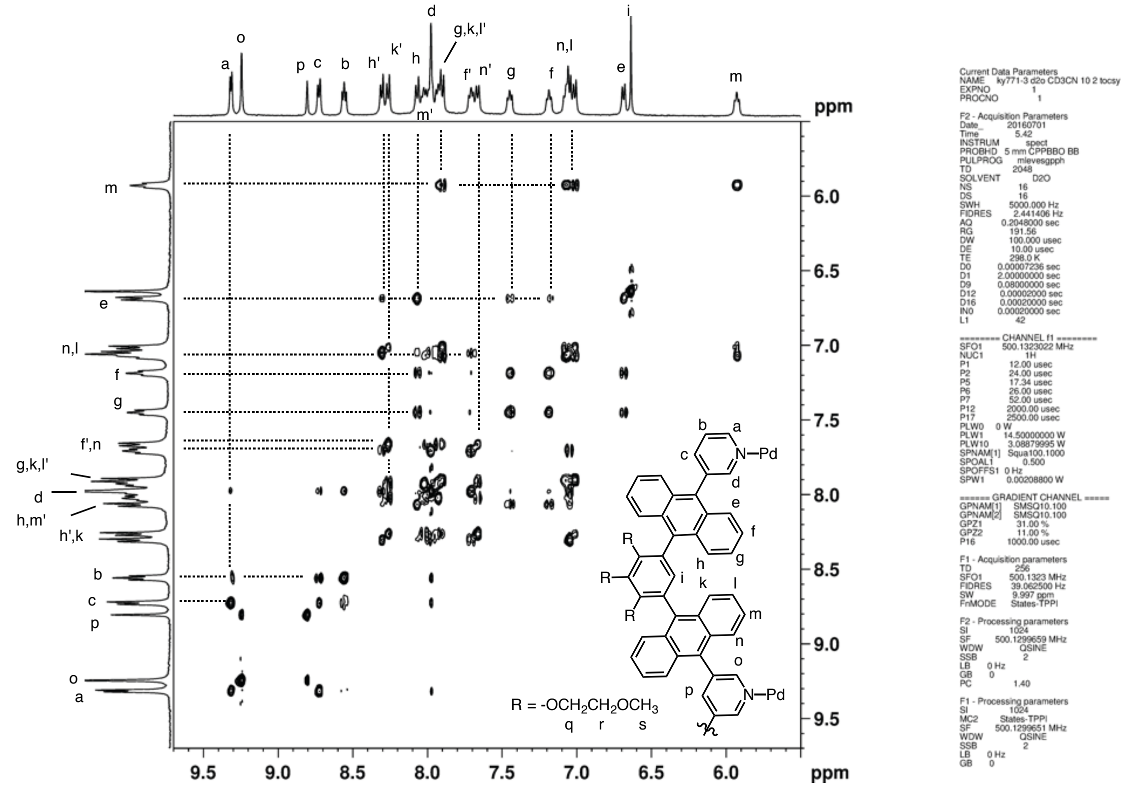

Supplementary Figure 30 | HH TOCSY spectrum (500 MHz, D_2O:CD_3CN = 5:1, room temperature) of 2b.

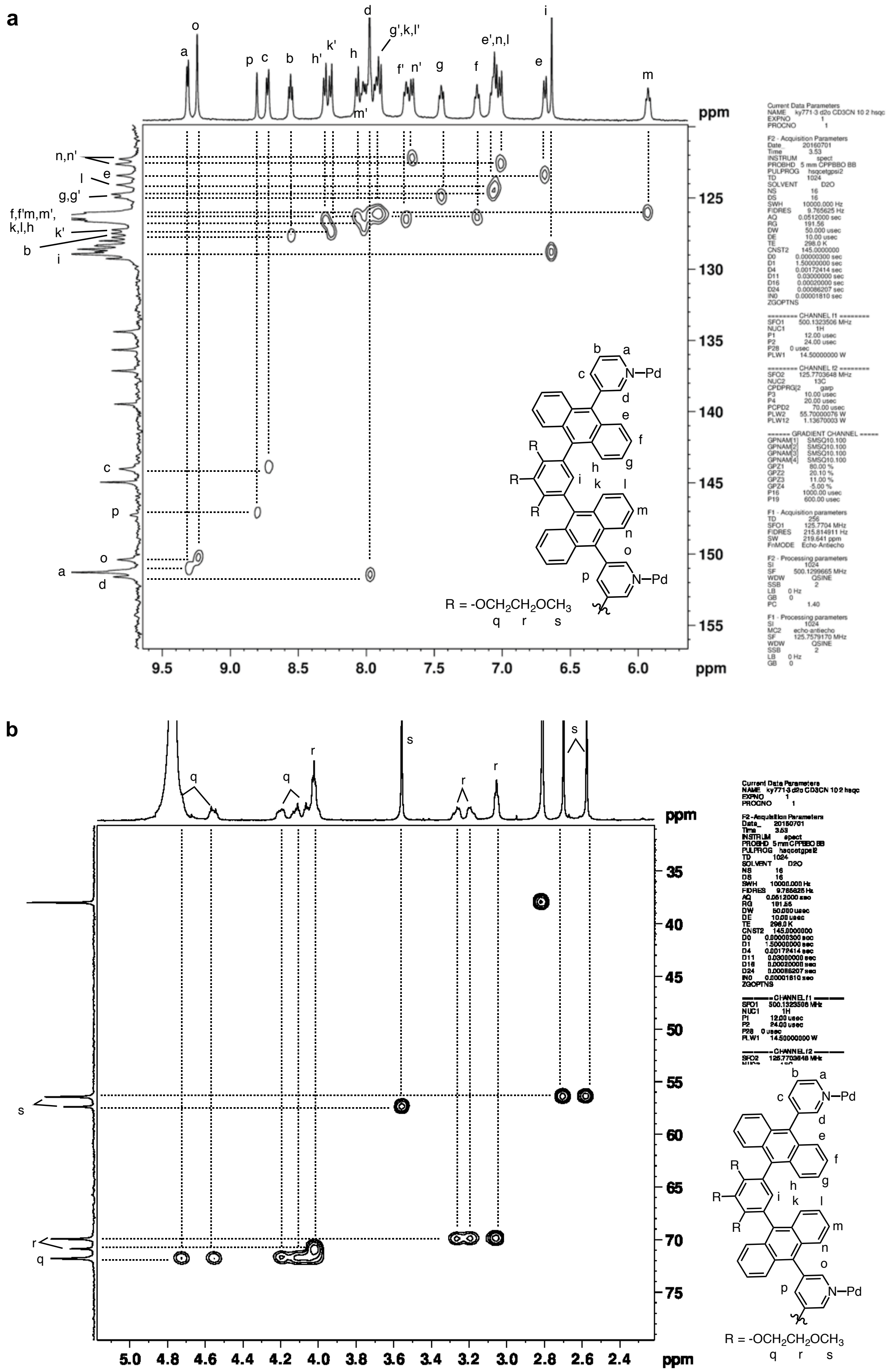

Supplementary Figure 31 | HSQC spectra (500 MHz, D_2O:CD_3CN = 5:1, room temperature) of **2b**. (a) The aromatic and (b) aliphatic regions.

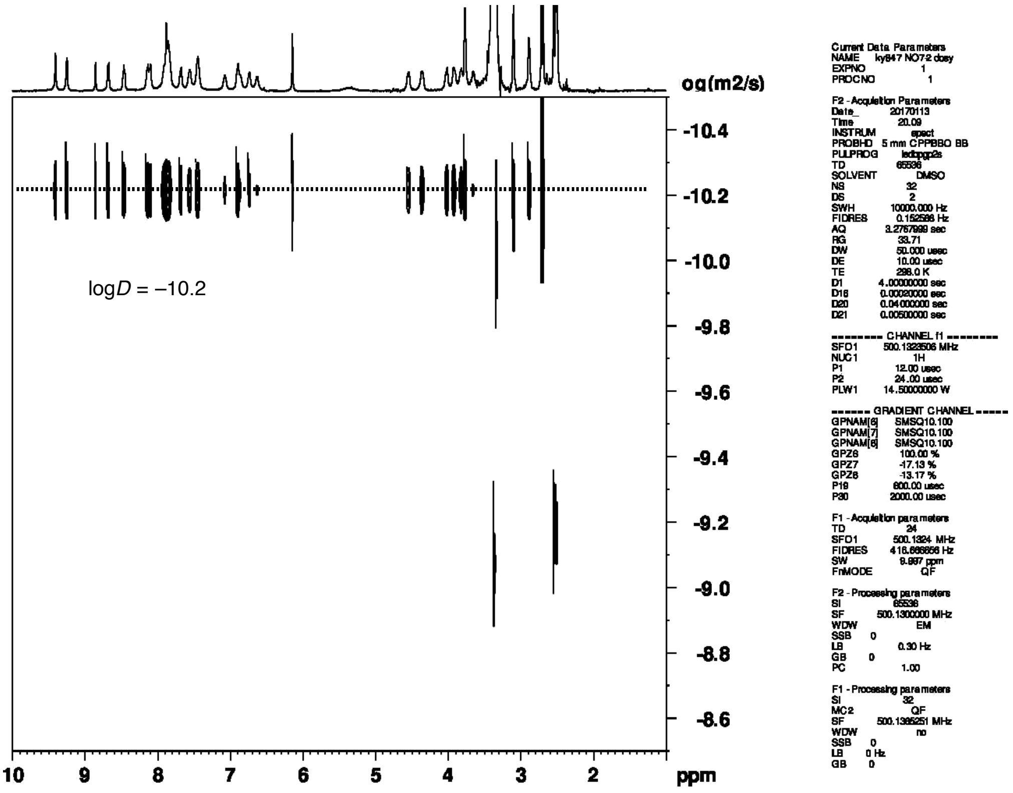

Supplementary Figure 32 | DOSY NMR spectrum (500 MHz, DMSO-d_6, 298 K) of **2b**.

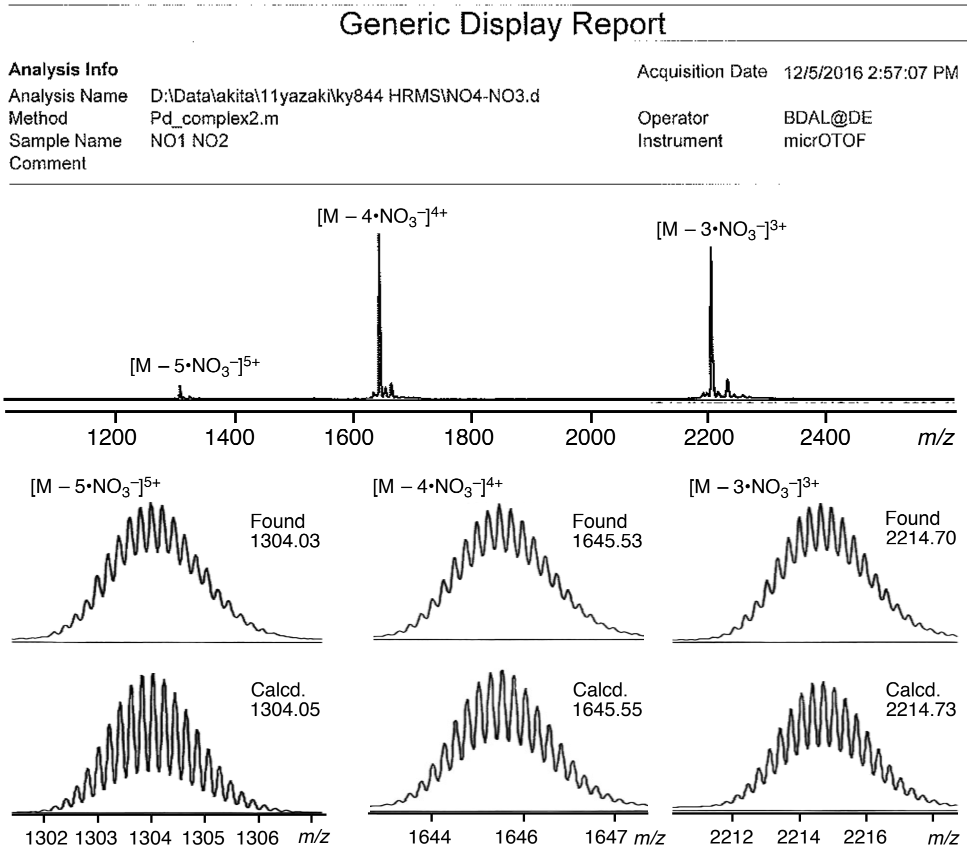

Supplementary Figure 33 | ESI-TOF MS spectrum (CH_3OH) of **2b**.

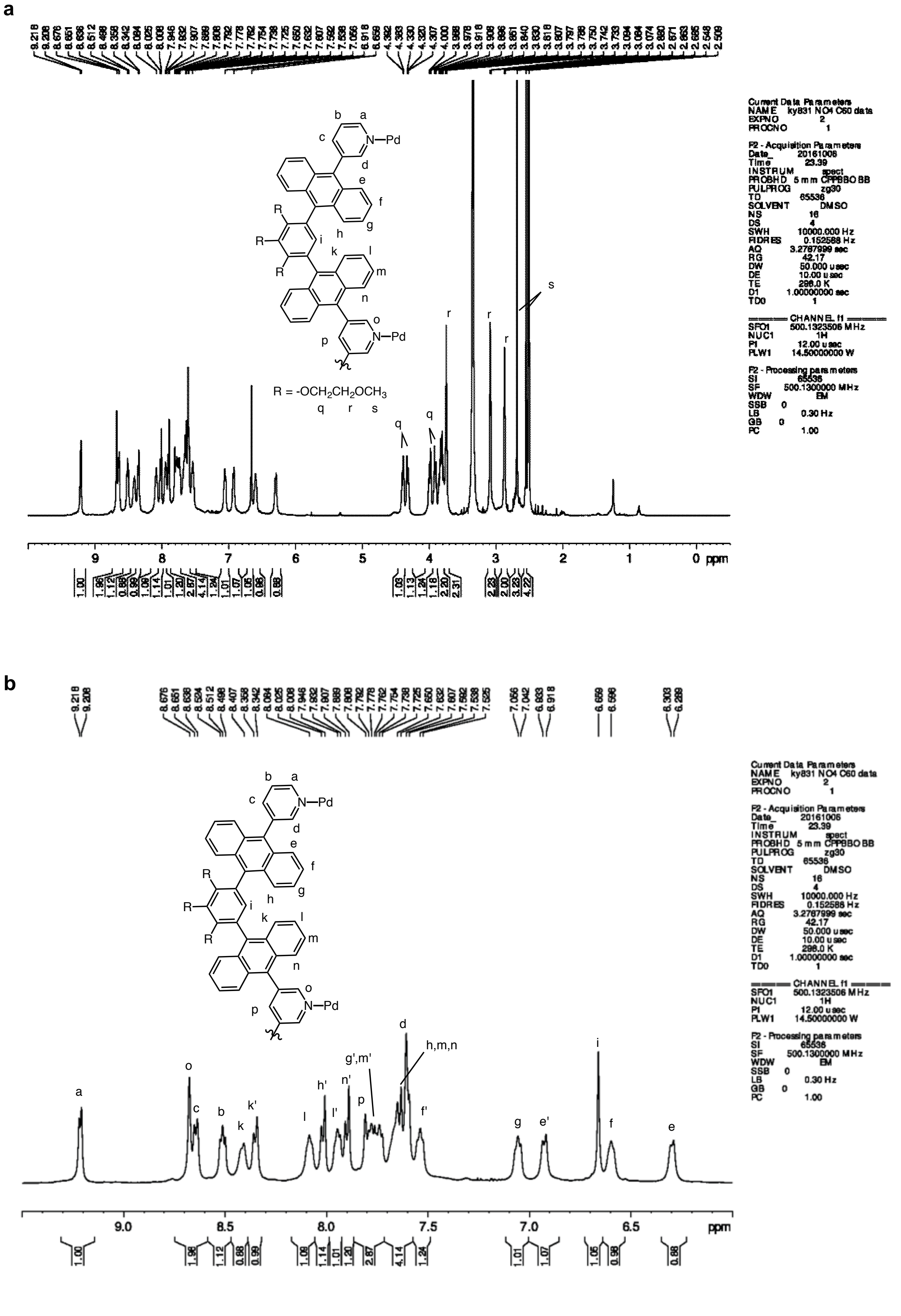

a
b
N–Pd
R = -OCH$_2$CH$_2$OCH$_3$
q r s
ppm
Current Data Parameters
NAME ky831 NO4 C60 data
EXPNO 2
PROCNO 1
F2 - Acquisition Parameters
Date_ 20161006
Time 23.39
INSTRUM spect
PROBHD 5 mm CPPBBO BB
PULPROG zg30
TD 65536
SOLVENT DMSO
NS 16
DS 4
SWH 10000.000 Hz
FIDRES 0.152588 Hz
AQ 3.2767999 sec
RG 42.17
DW 50.000 usec
DE 10.00 usec
TE 298.0 K
D1 1.00000000 sec
TD0 1
CHANNEL f1
SFO1 500.1323506 MHz
NUC1 1H
P1 12.00 usec
PLW1 14.50000000 W
F2 - Processing parameters
SI 65536
SF 500.1300000 MHz
WDW EM
SSB 0
LB 0.30 Hz
GB 0
PC 1.00

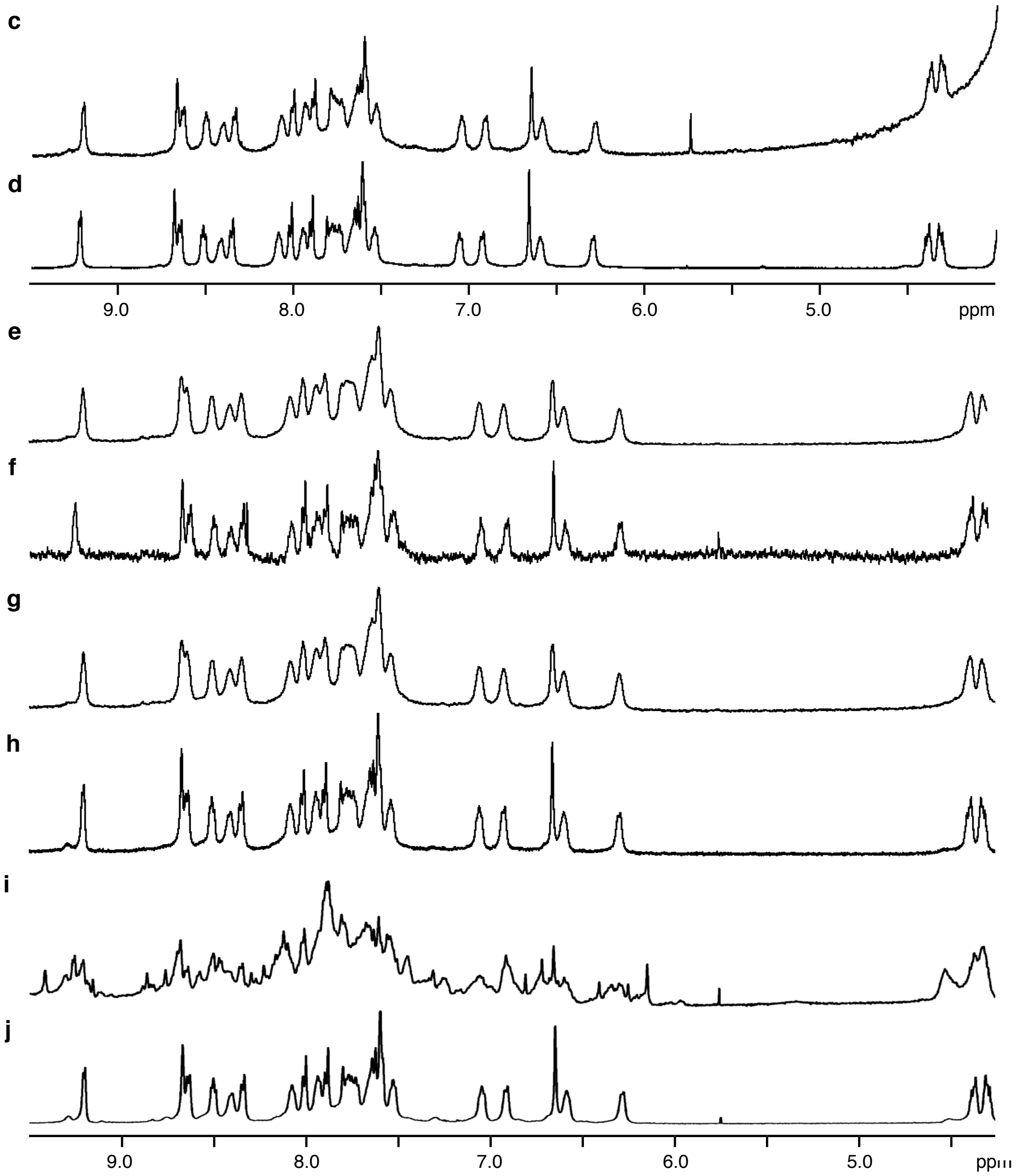

Supplementary Figure 34 | ^{1}H NMR spectra (500 MHz, DMSO-d_6, room temperature) of $(C_{60})_2$@**3b**. (a) The aromatic and aliphatic and (b) aromatic regions. The ^{1}H NMR spectra of $(C_{60})_2$@**3b** obtained by (c) the route 1 and (d) the route 2. Concentration-dependent ^{1}H NMR spectra of $(C_{60})_2$@**3b** in (e) 0.36 mM and (f) 5.0 μM and time-dependent ^{1}H NMR spectra of $(C_{60})_2$@**3b** after (g) 10 min and (h) 5 d at room temperature. ^{1}H NMR spectra of a mixture of **2b** and C_{60} after (i) 6 h at 70 ºC and (j) 3 h at 110 ºC.

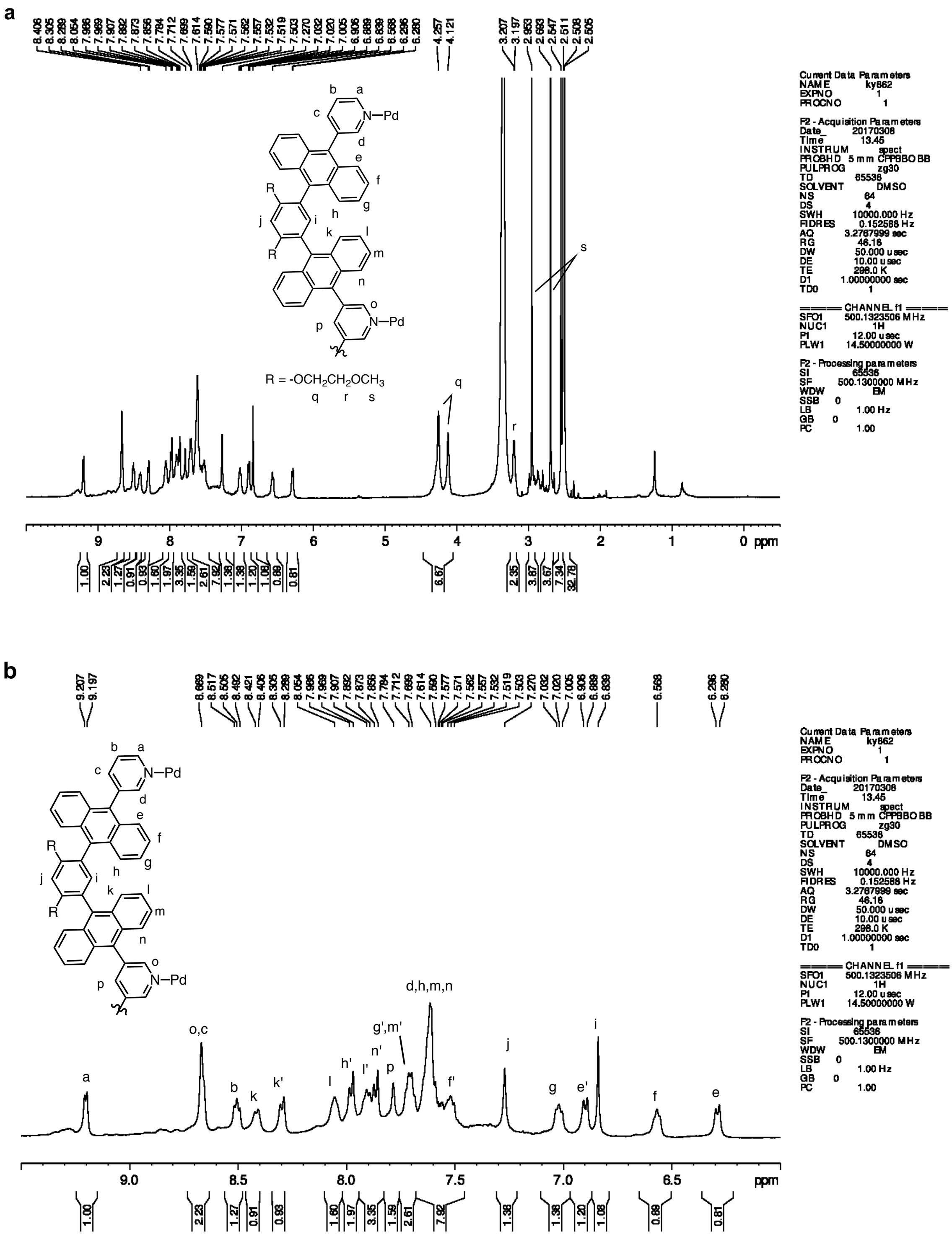

Supplementary Figure 35 | ^{1}H NMR spectra (500 MHz, DMSO-d_6, room temperature) of $(C_{60})_2$@**3a**. (a) The aromatic and aliphatic and (b) aromatic regions.

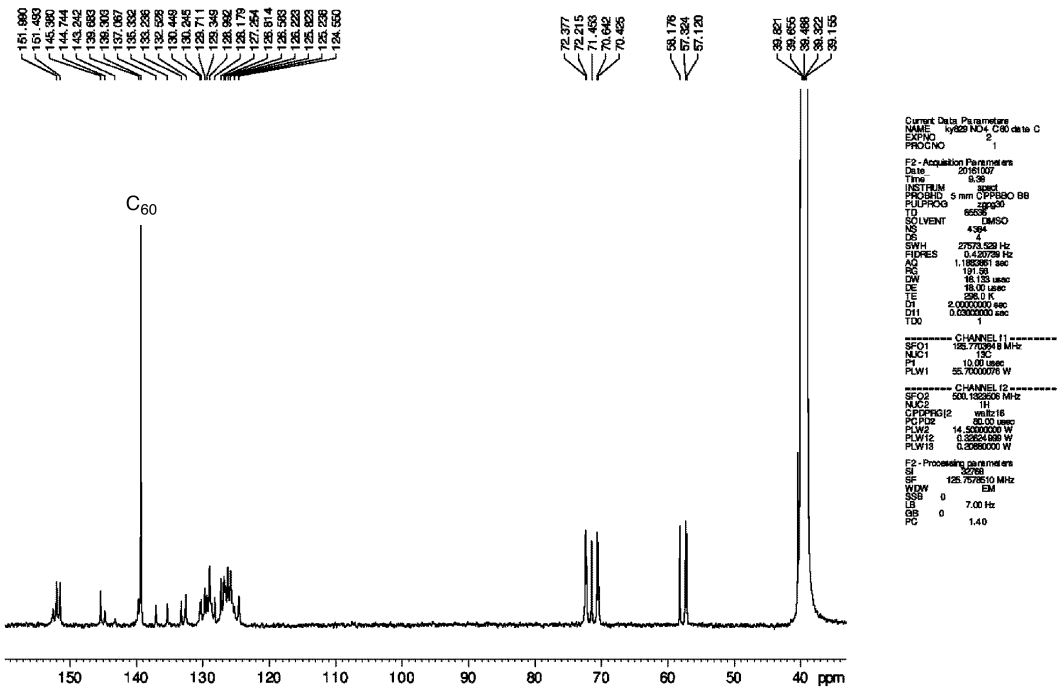

Supplementary Figure 36 | ** ^{13}C NMR spectrum (125 MHz, DMSO-d_6, room temperature) of $(C_{60})_2$@3b**.

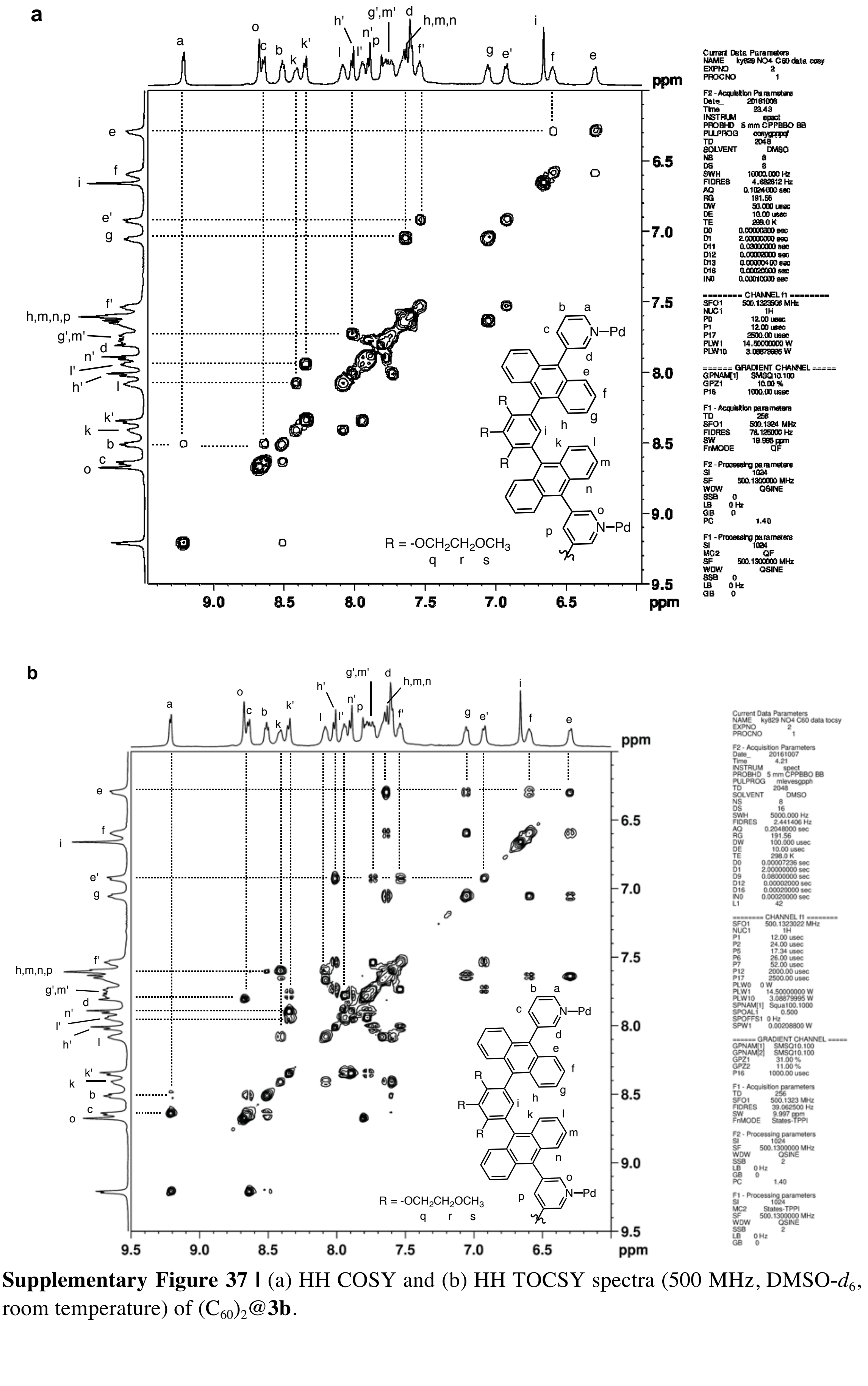

Supplementary Figure 37 | (a) HH COSY and (b) HH TOCSY spectra (500 MHz, DMSO-d_6, room temperature) of $(C_{60})_2$@**3b**.

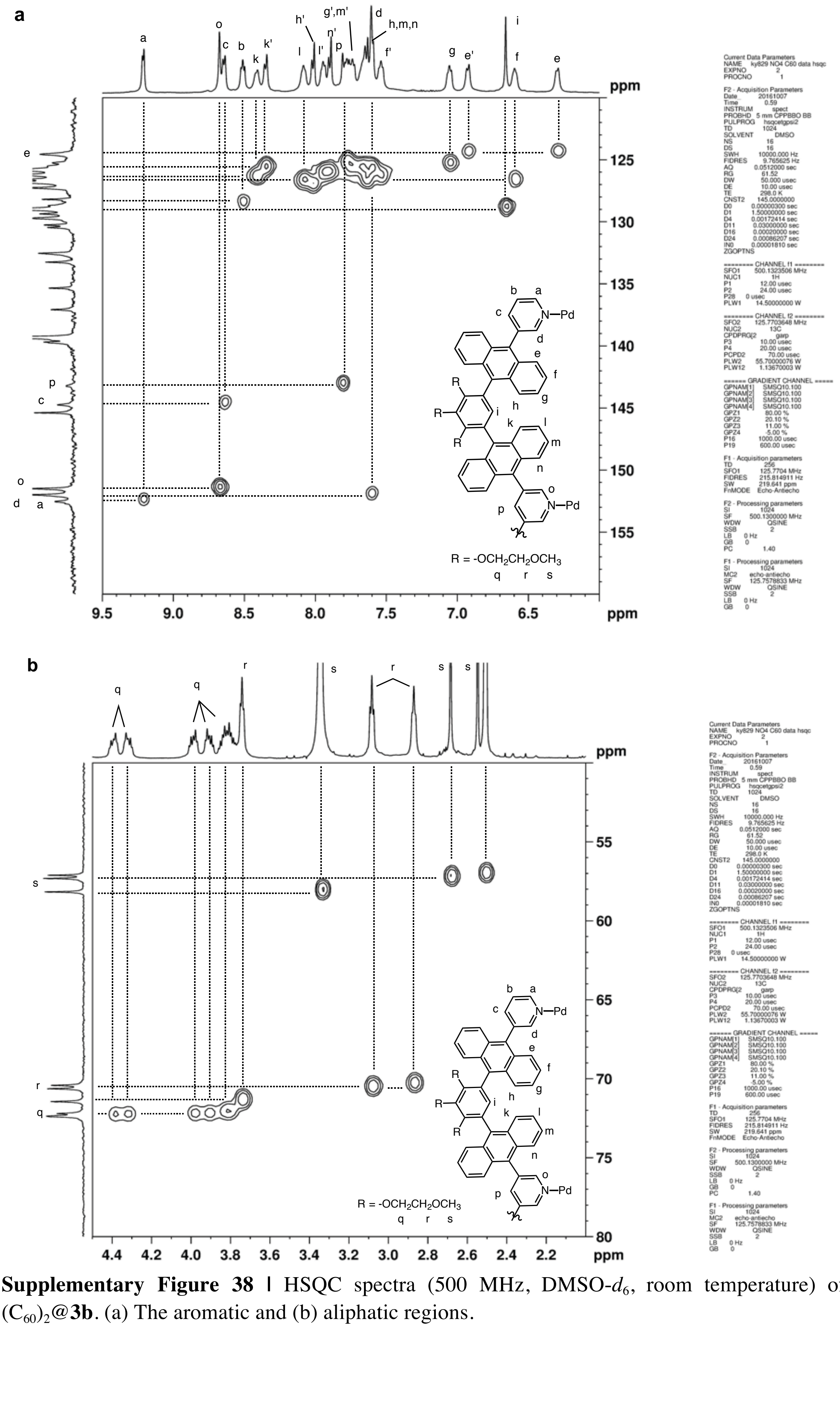

Supplementary Figure 38 | ** HSQC spectra (500 MHz, DMSO-d_6, room temperature) of $(C_{60})_2$@3b**. (a) The aromatic and (b) aliphatic regions.

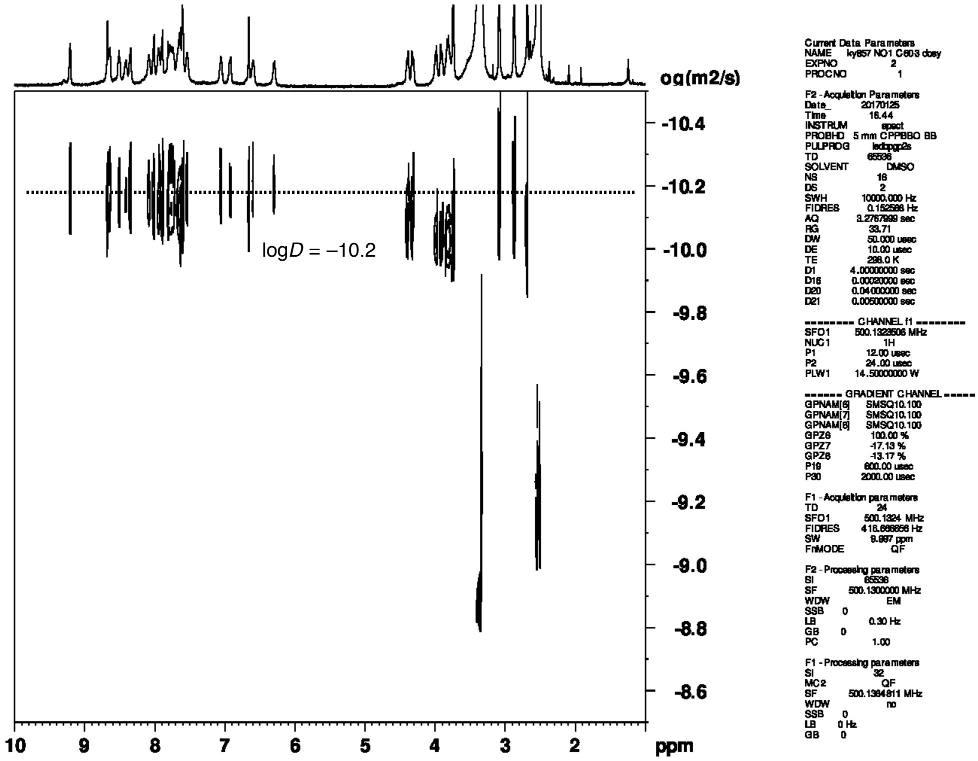

Supplementary Figure 39 | DOSY NMR spectrum (500 MHz, DMSO-d_6, 298 K) of $(C_{60})_2$@**3b**.

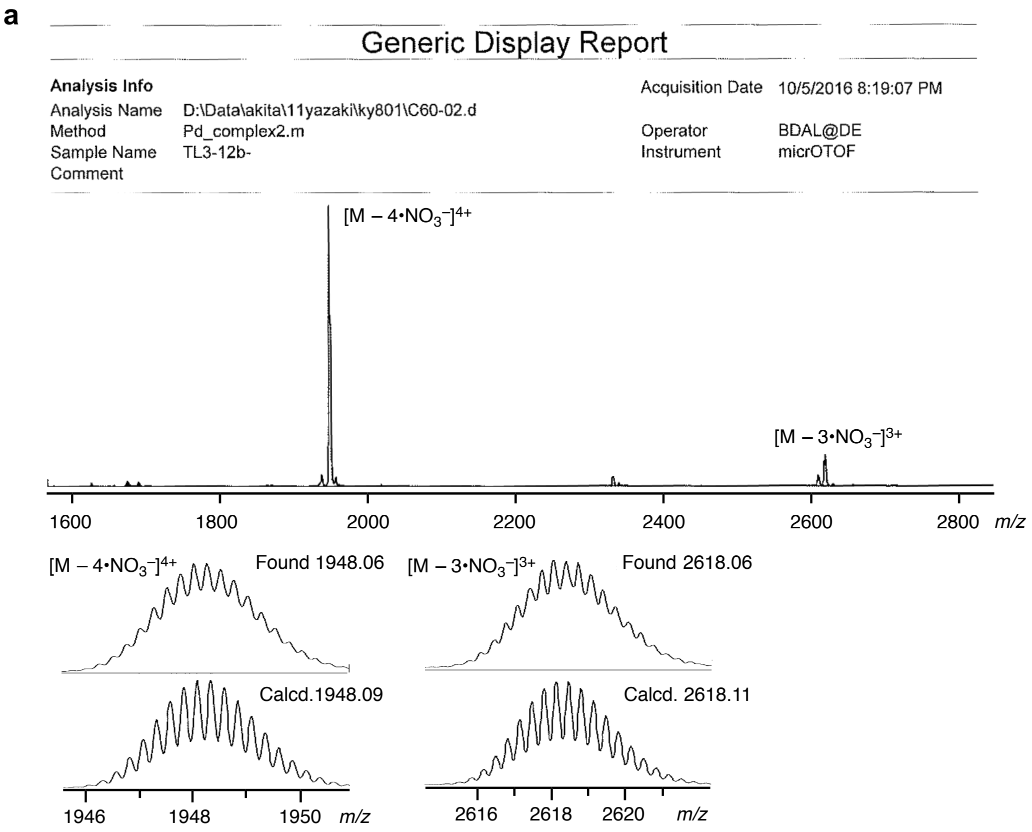

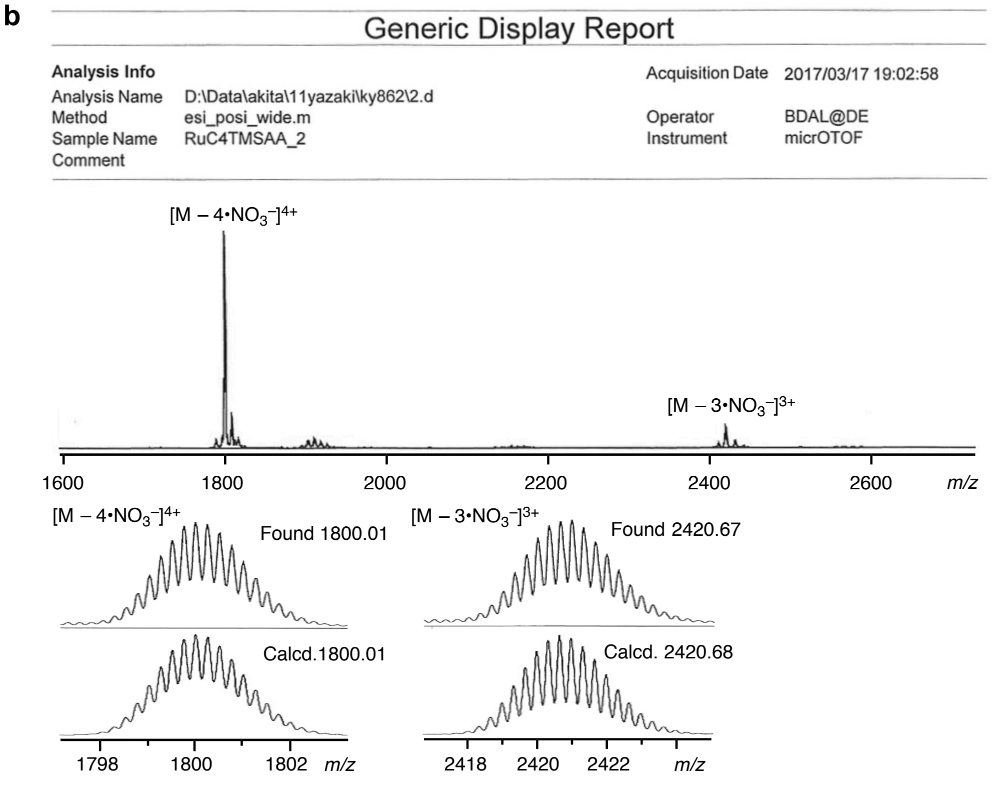

Supplementary Figure 40 | ESI-TOF MS spectra (CH_3CN) of (a) $(C_{60})_2$@**3b** and (b) $(C_{60})_2$@**3a**.

a

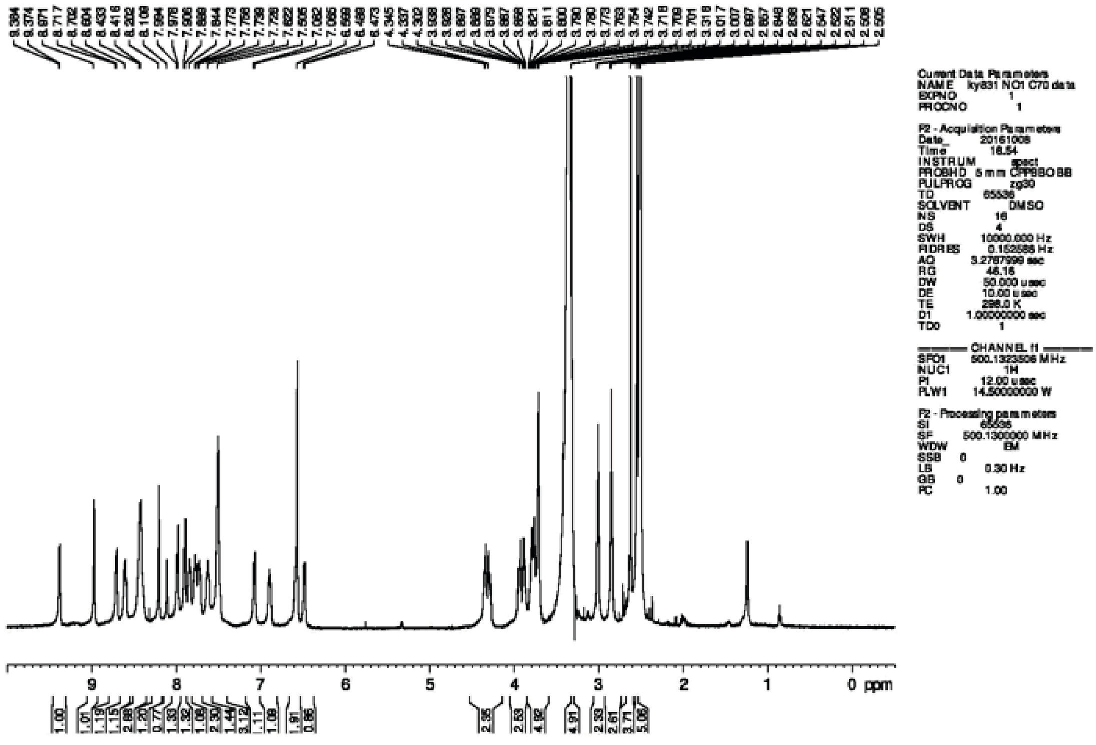

b

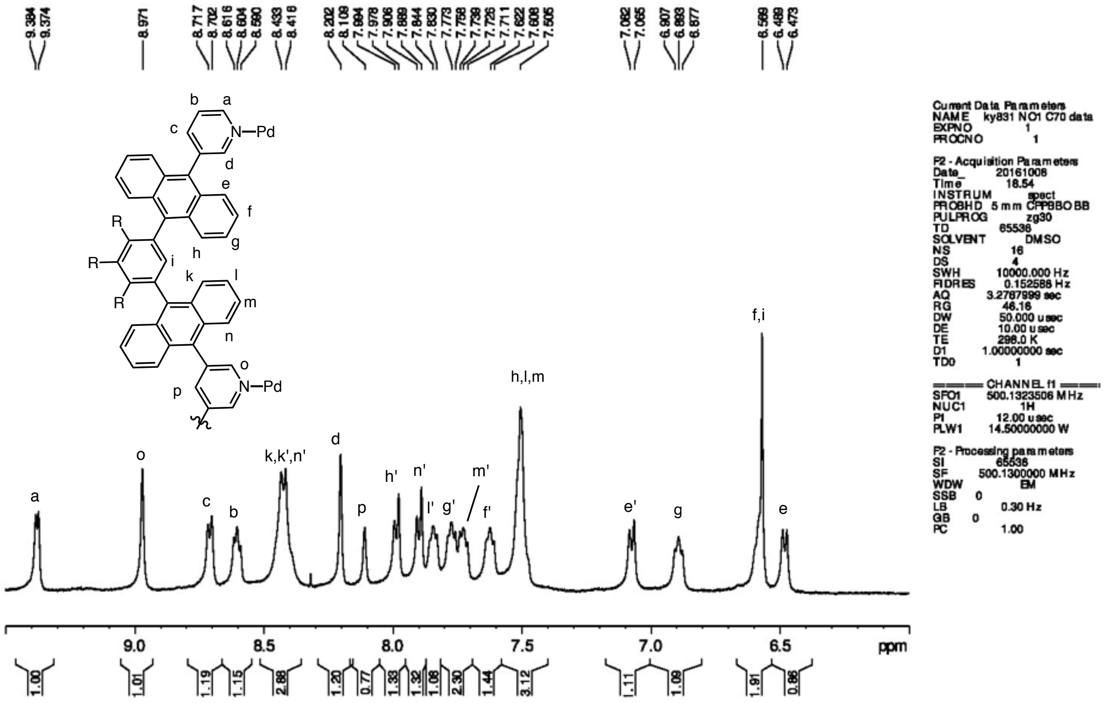

Supplementary Figure 41 | **^{1}H NMR spectra (500 MHz, DMSO-d_6, room temperature) of $(C_{70})_2$@3b**. (a) The aromatic and aliphatic and (b) aromatic regions.

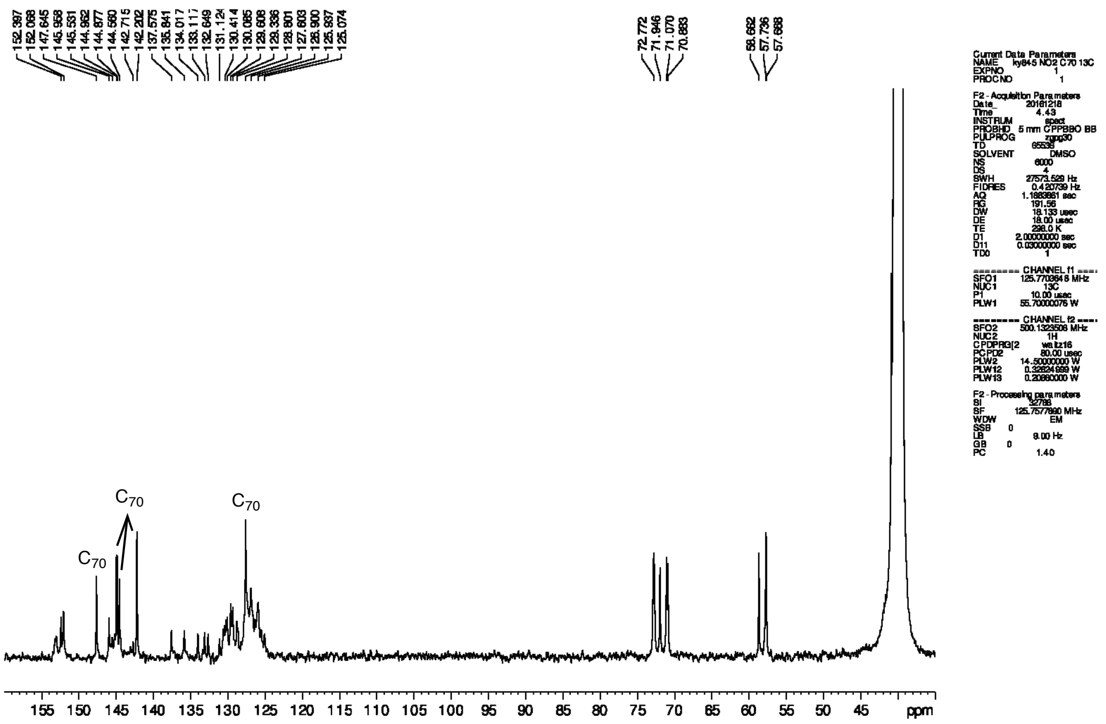

Supplementary Figure 42 | ^{13}C NMR spectrum (125 MHz, DMSO-d_6, room temperature) of $(C_{70})_2$@**3b**.

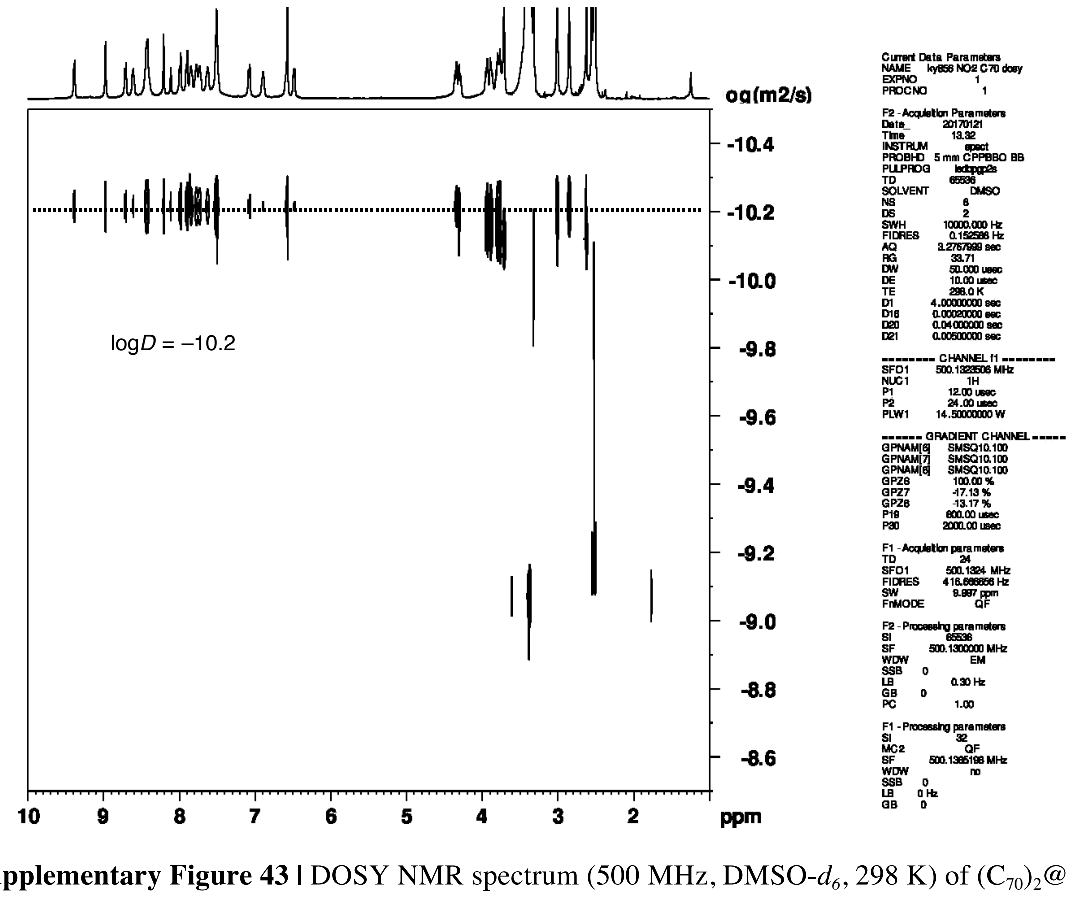

Supplementary Figure 43 | DOSY NMR spectrum (500 MHz, DMSO-d_6, 298 K) of $(C_{70})_2$@**3b**.

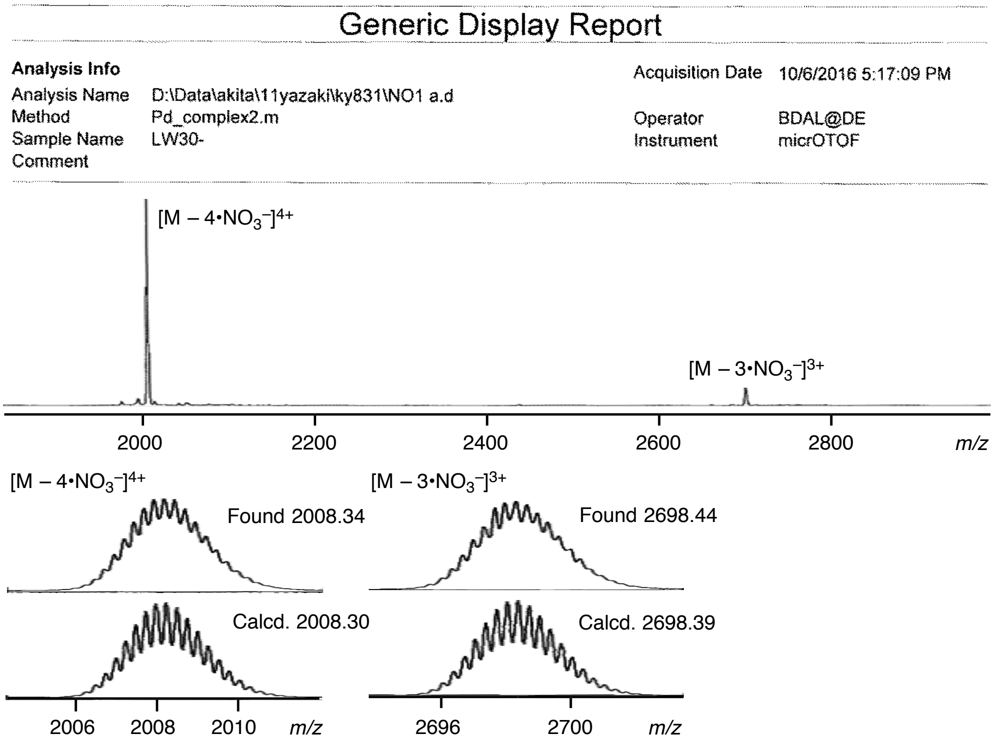

Supplementary Figure 44 | ESI-TOF MS spectrum (CH_3CN) of $(C_{70})_2$@**3b**.

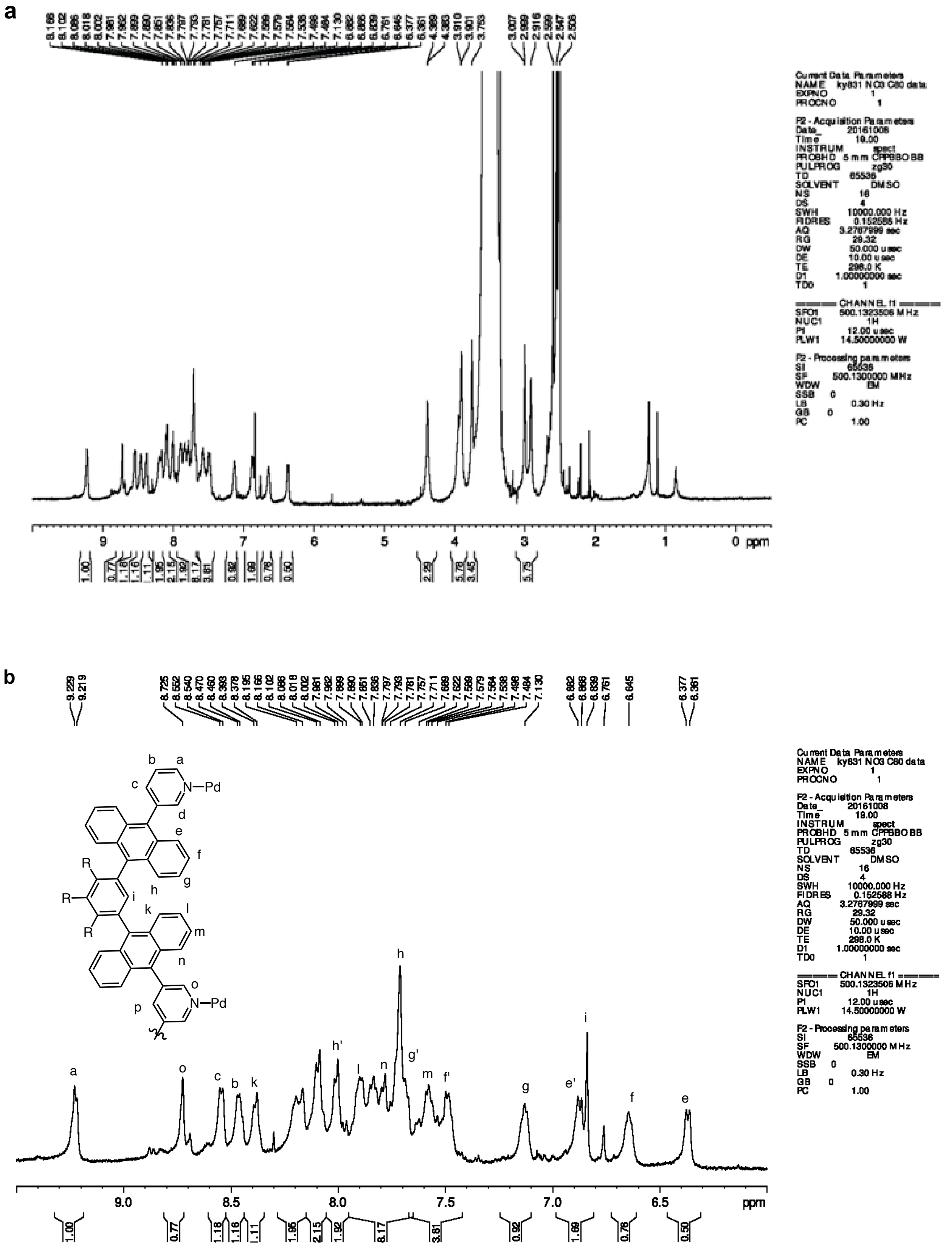

Supplementary Figure 45 | ^{1}H NMR spectra (500 MHz, DMSO-d_6, room temperature) of $(Sc_3N@C_{80})_2@$**3b**. (a) The aromatic and aliphatic and (b) aromatic regions.

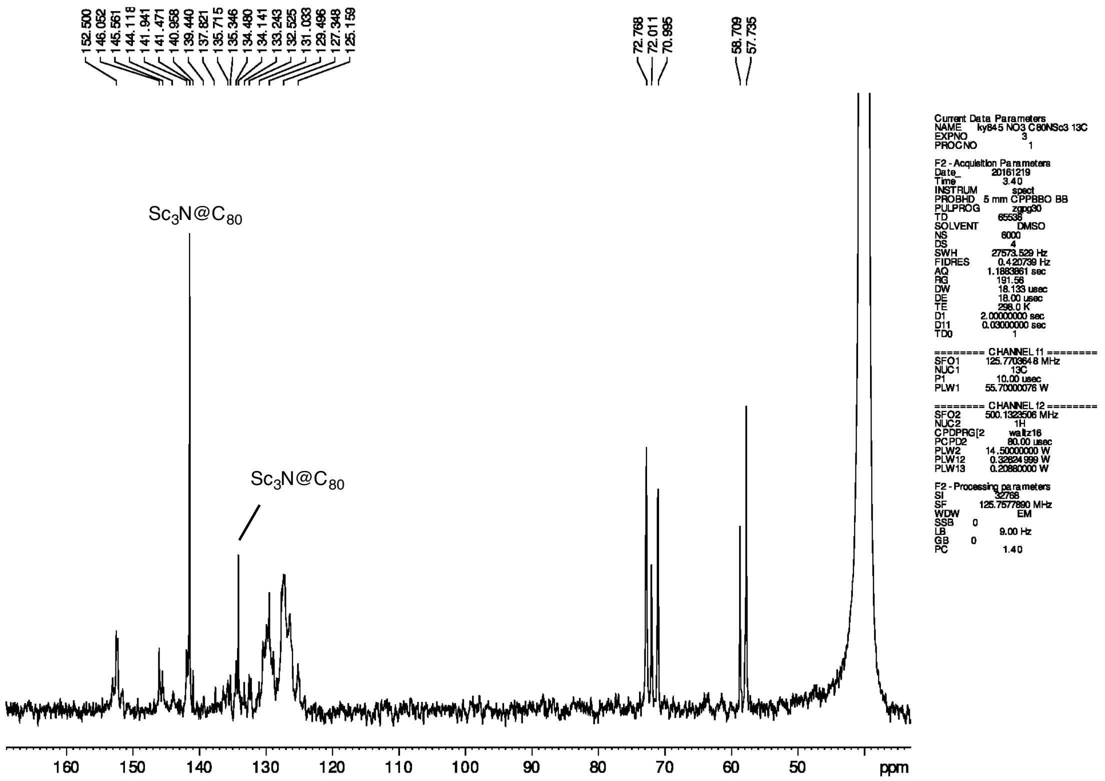

Supplementary Figure 46 | ^{13}C NMR spectrum (125 MHz, DMSO-d_6, room temperature) of $(Sc_3N@C_{80})_2$@**3b**.

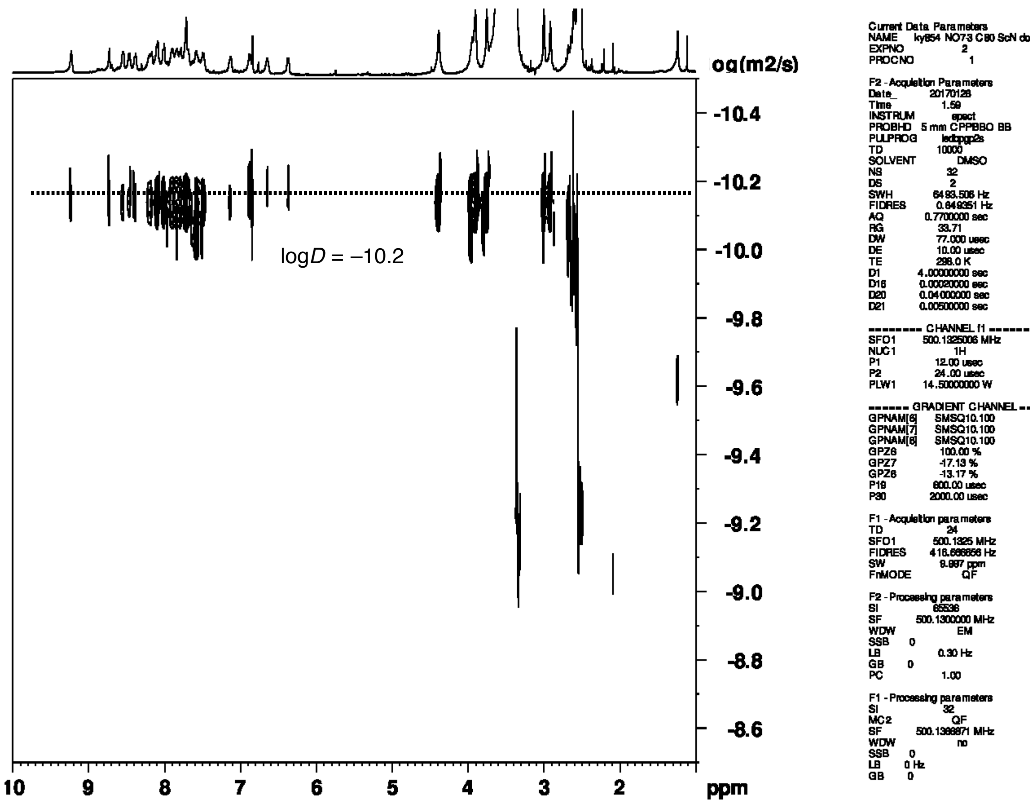

Supplementary Figure 47 | DOSY NMR spectrum (500 MHz, DMSO-d_6, 298 K) of $(Sc_3N@C_{80})_2$@**3b**.

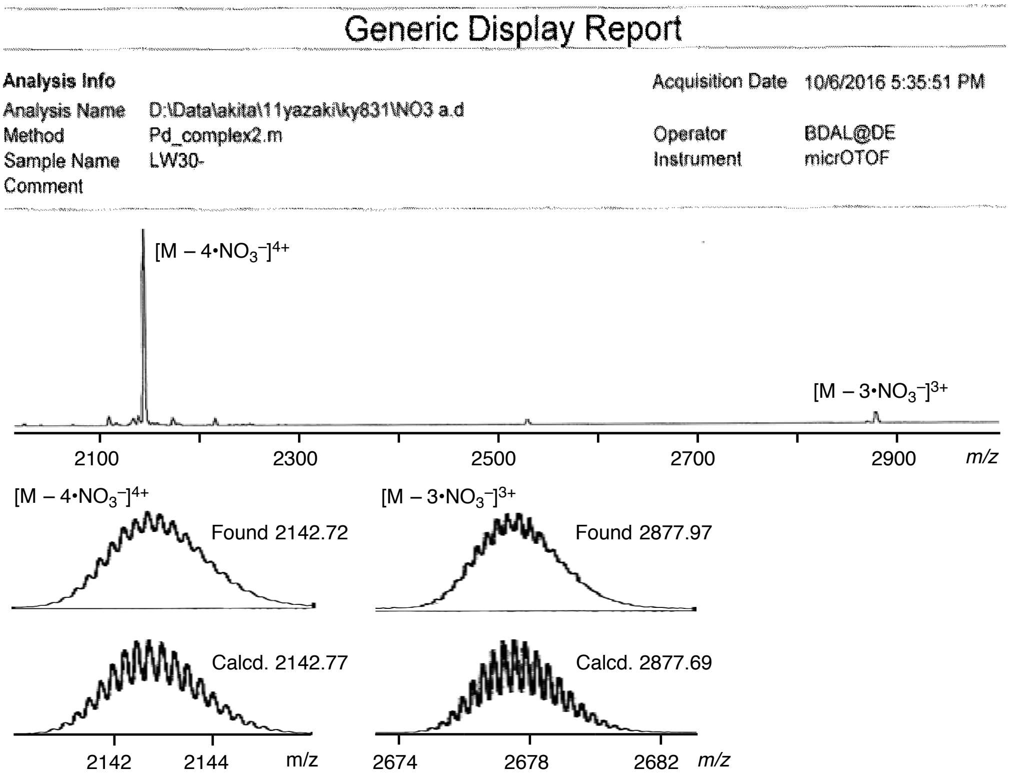

Supplementary Figure 48 | ESI-TOF MS spectrum (CH_3CN) of $(Sc_3N@C_{80})_2@$**3b**.

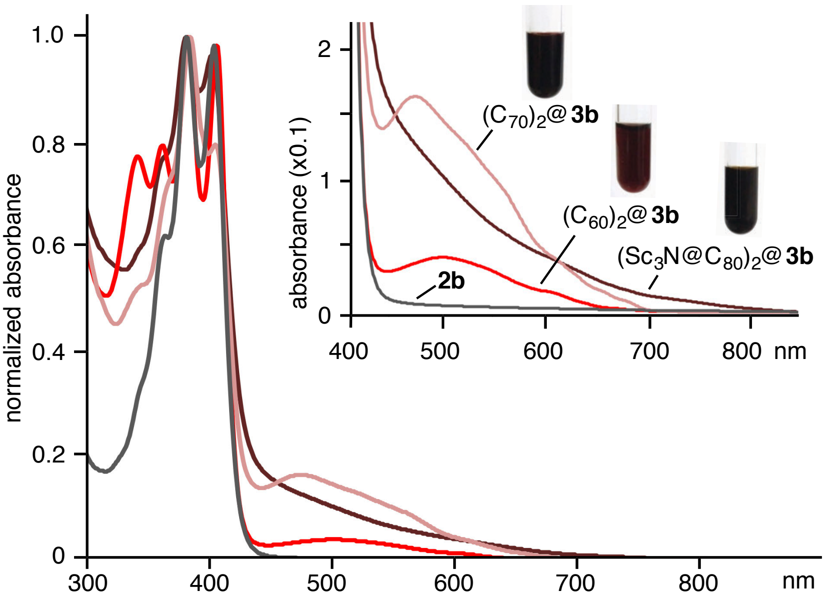

Supplementary Figure 49 | Normalized UV-visible spectra (~0.3 mM, DMSO, room temperature) and photographs of $(C_{60})_2@$**3b**, $(C_{70})_2@$**3b**, and $(Sc_3N@C_{80})_2@$**3b**.

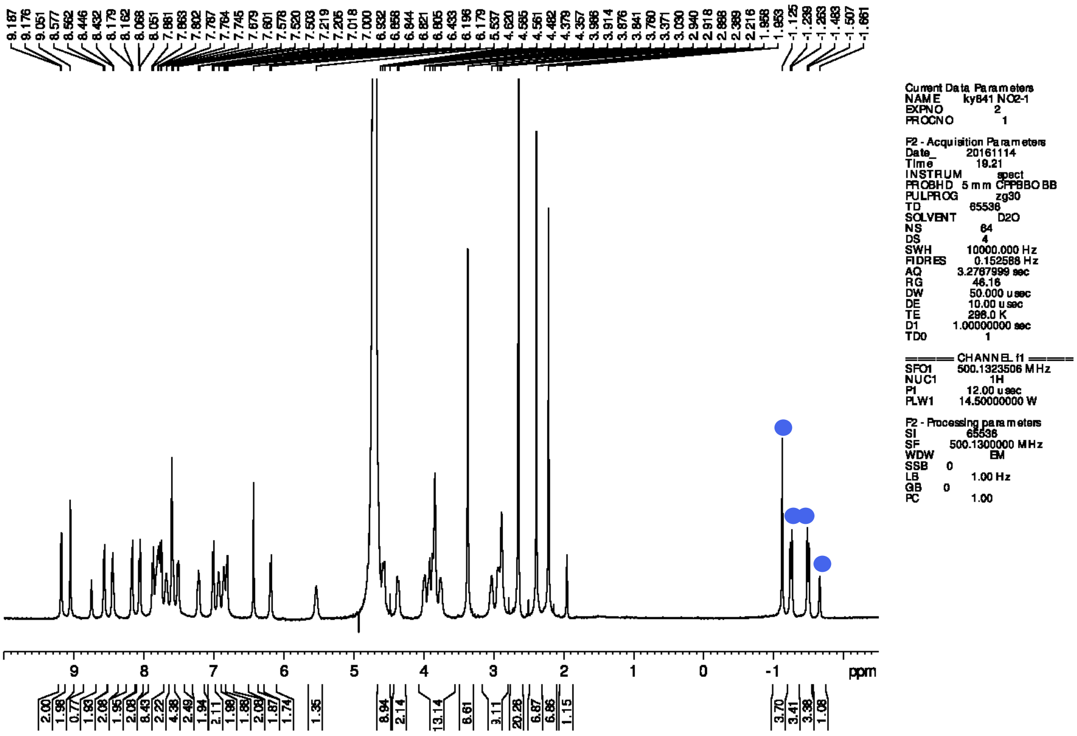

Supplementary Figure 50 | ^{1}H NMR spectrum (500 MHz, $D_2O:CD_3CN$ = 100:1, room temperature) of (**4a**/**4a**)@**2b**.

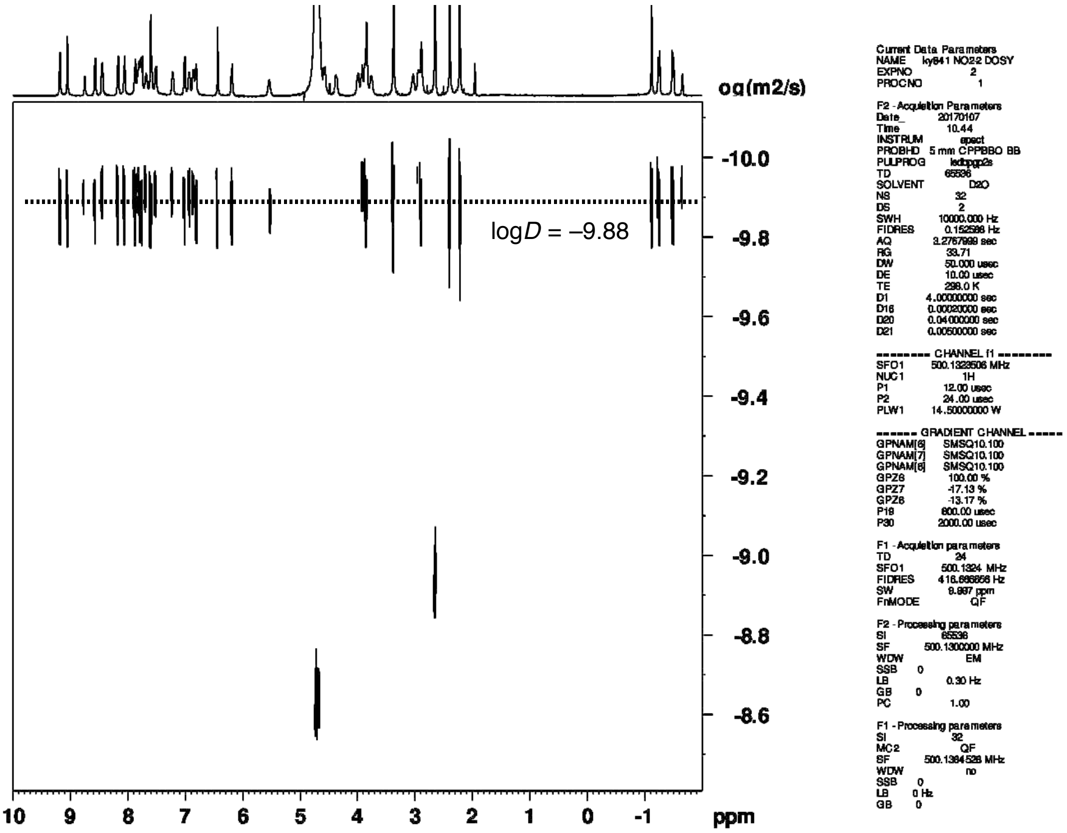

Supplementary Figure 51 | DOSY NMR spectrum (500 MHz, $D_2O:CD_3CN$ = 100:1, 298 K) of (**4a**/**4a**)@**2b**.

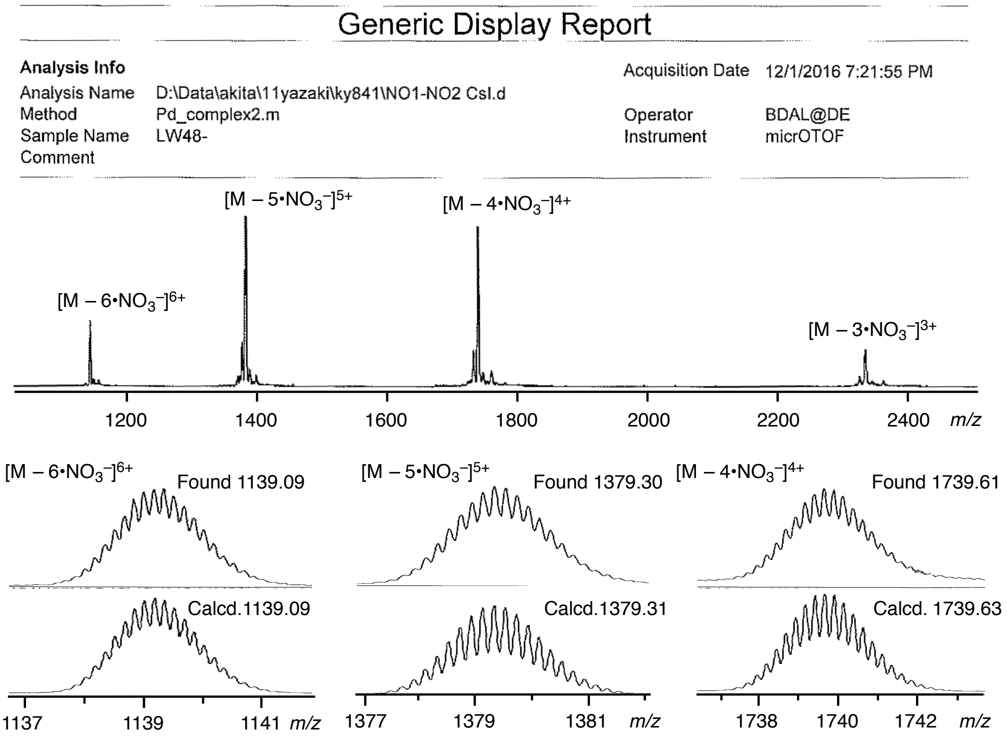

Supplementary Figure 52 | ESI-TOF MS spectrum (H_2O:CH_3CN = 100:1) of (**4a**/**4a**)@**2b**.

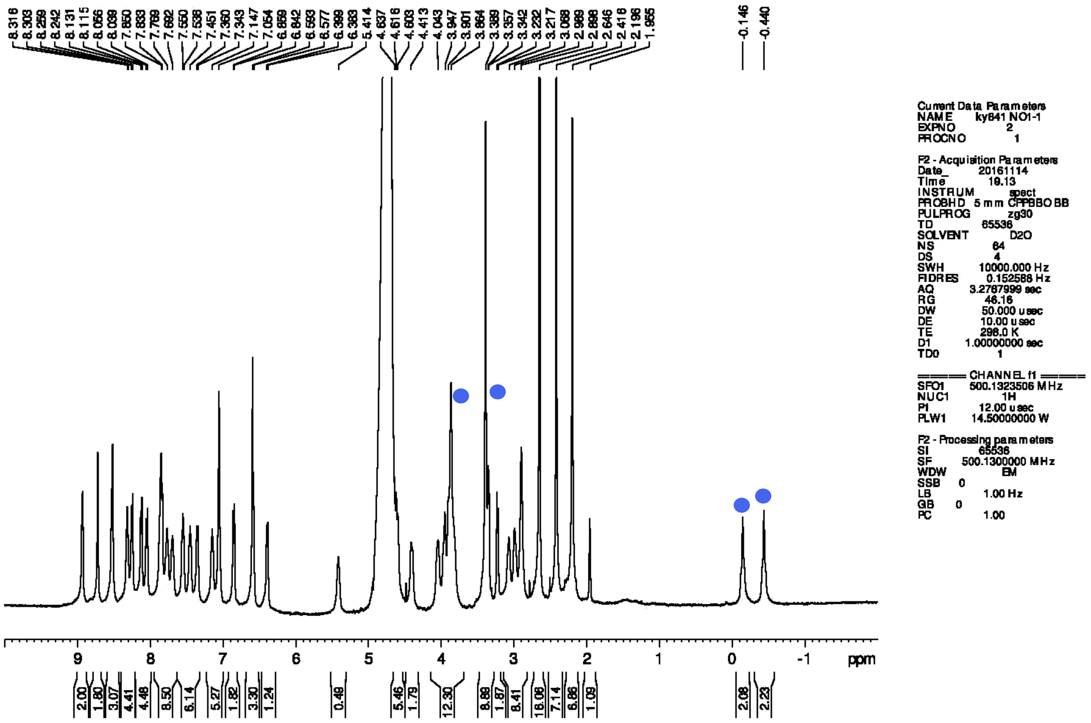

Supplementary Figure 53 | ^1H NMR spectrum (500 MHz, D_2O:CD_3CN = 100:1, room temperature) of (**4c**/**4c**)@**2b**.

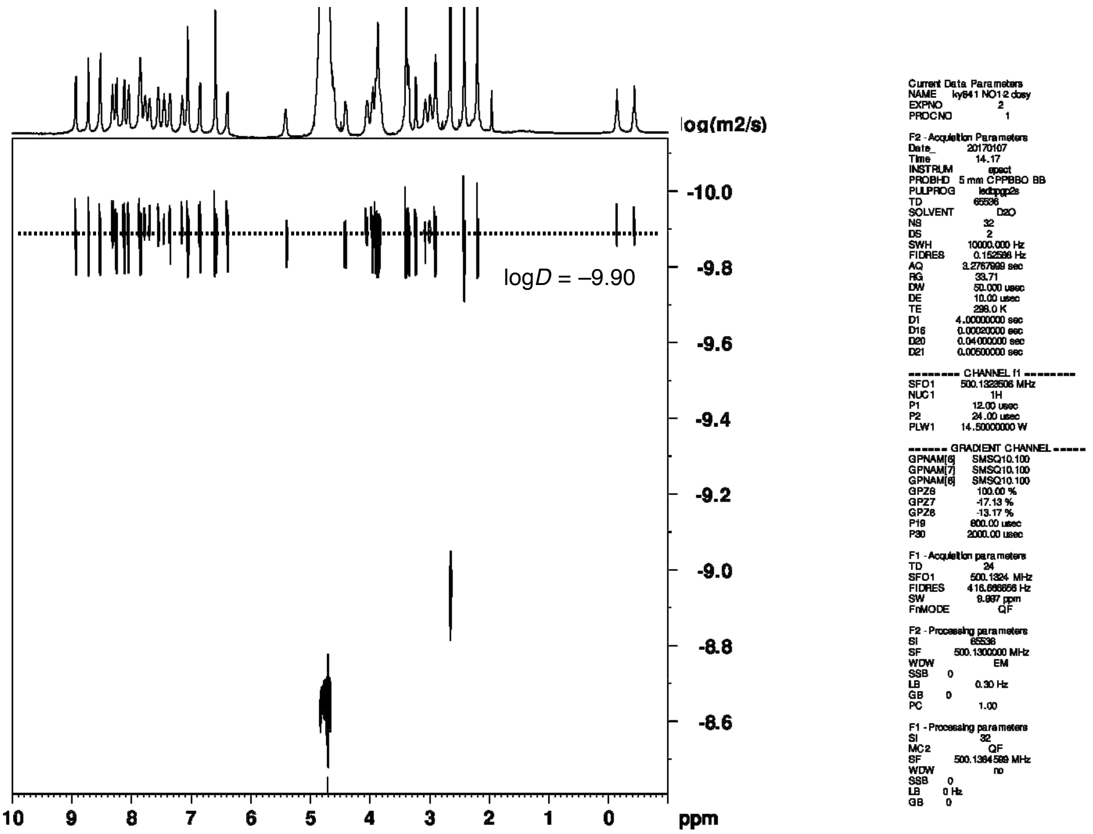

Supplementary Figure 54 | DOSY NMR spectrum (500 MHz, D_2O:CD_3CN = 100:1, 298 K) of **(4c/4c)@2b**.

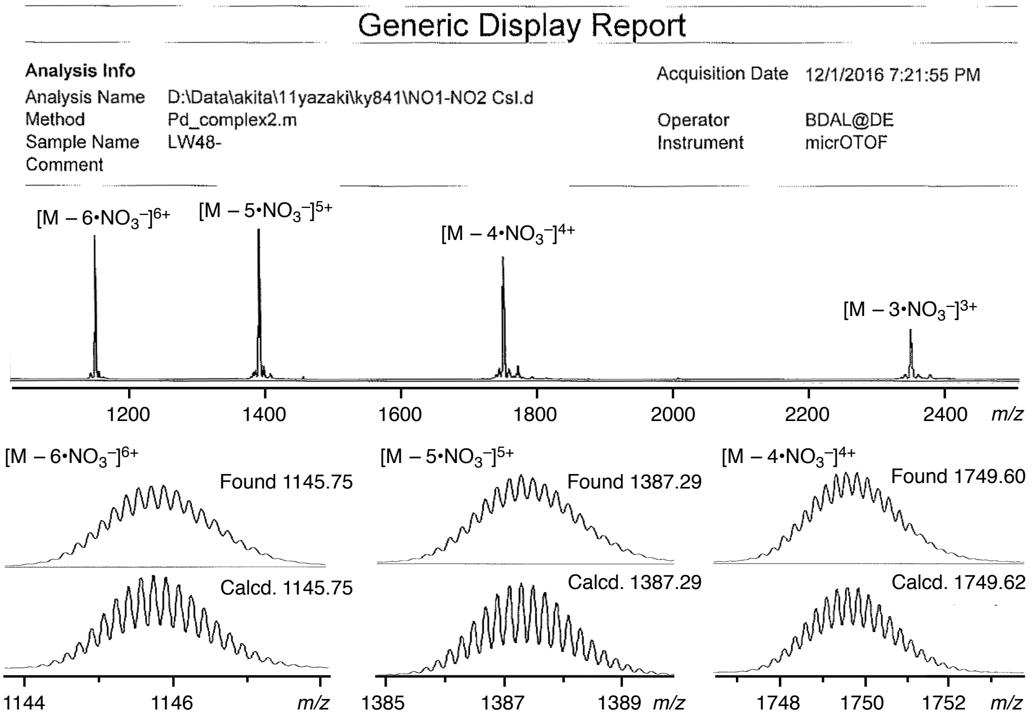

Supplementary Figure 55 | ESI-TOF MS spectrum (H_2O:CH_3CN = 100:1) of **(4c/4c)@2b**.

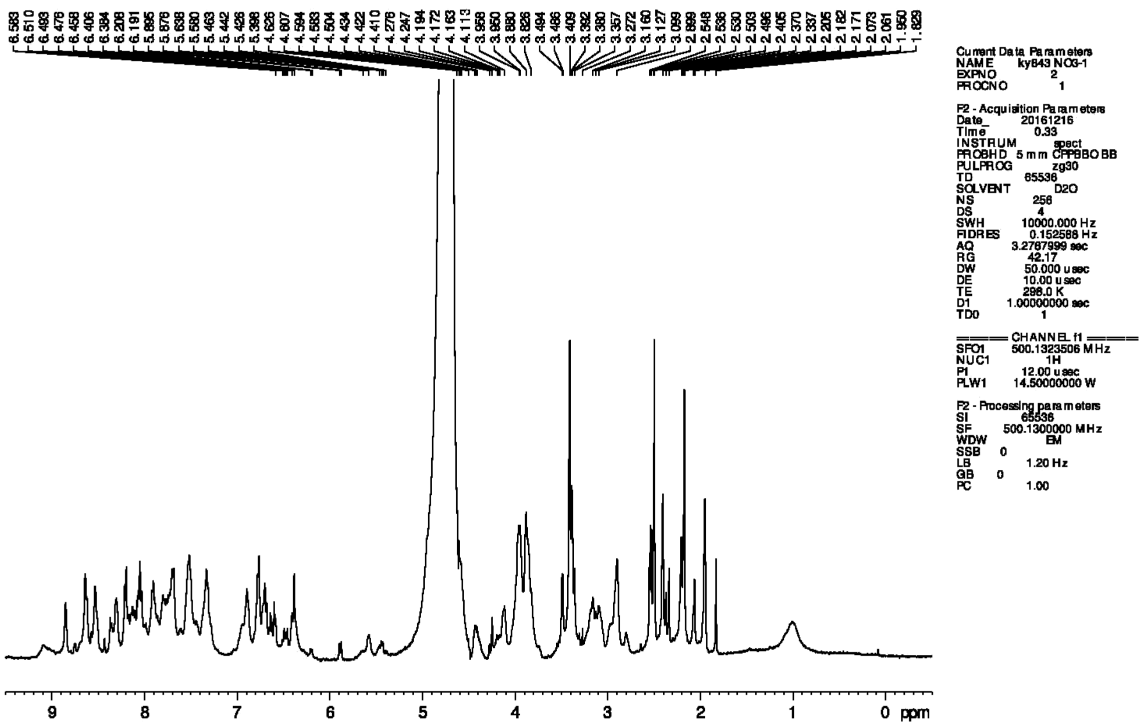

Supplementary Figure 56 | ^1H NMR spectrum (500 MHz, D_2O:CD_3CN = 100:1, room temperature) of (**4b**/(**4b**)$_2$)@**2b**.

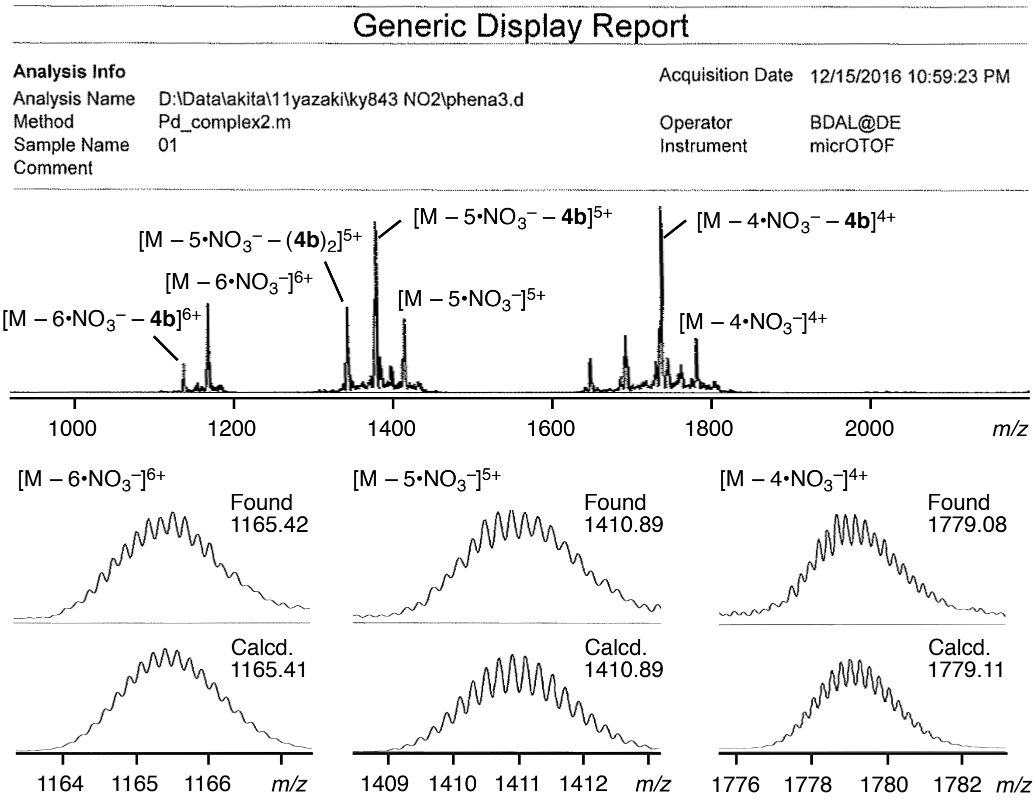

Supplementary Figure 57 | ESI-TOF MS spectrum (H_2O:CH_3CN = 100:1) of (**4b**/(**4b**)$_2$)@**2b**.

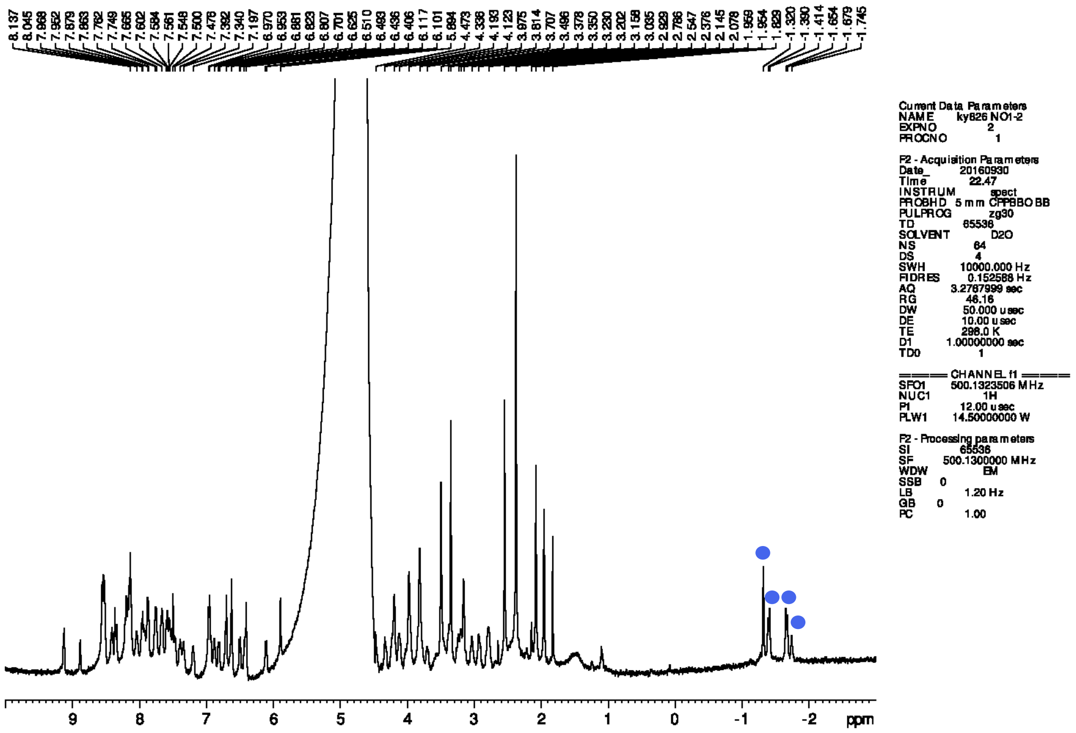

Supplementary Figure 58 | ^{1}H NMR spectrum (500 MHz, D_2O:CD_3CN = 100:1, room temperature) of (**4a**/(**4b**)$_2$)@**2b**.

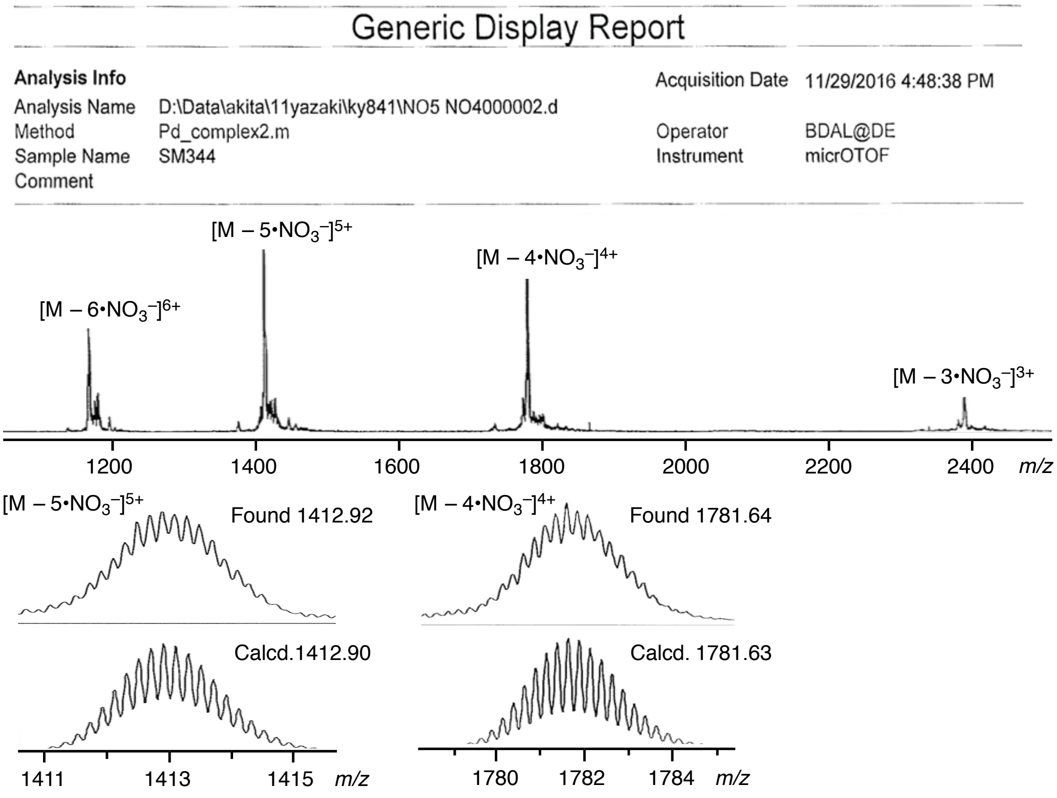

Supplementary Figure 59 | ESI-TOF MS spectrum (H_2O:CH_3CN = 100:1) of (**4a**/(**4b**)$_2$)@**2b**.

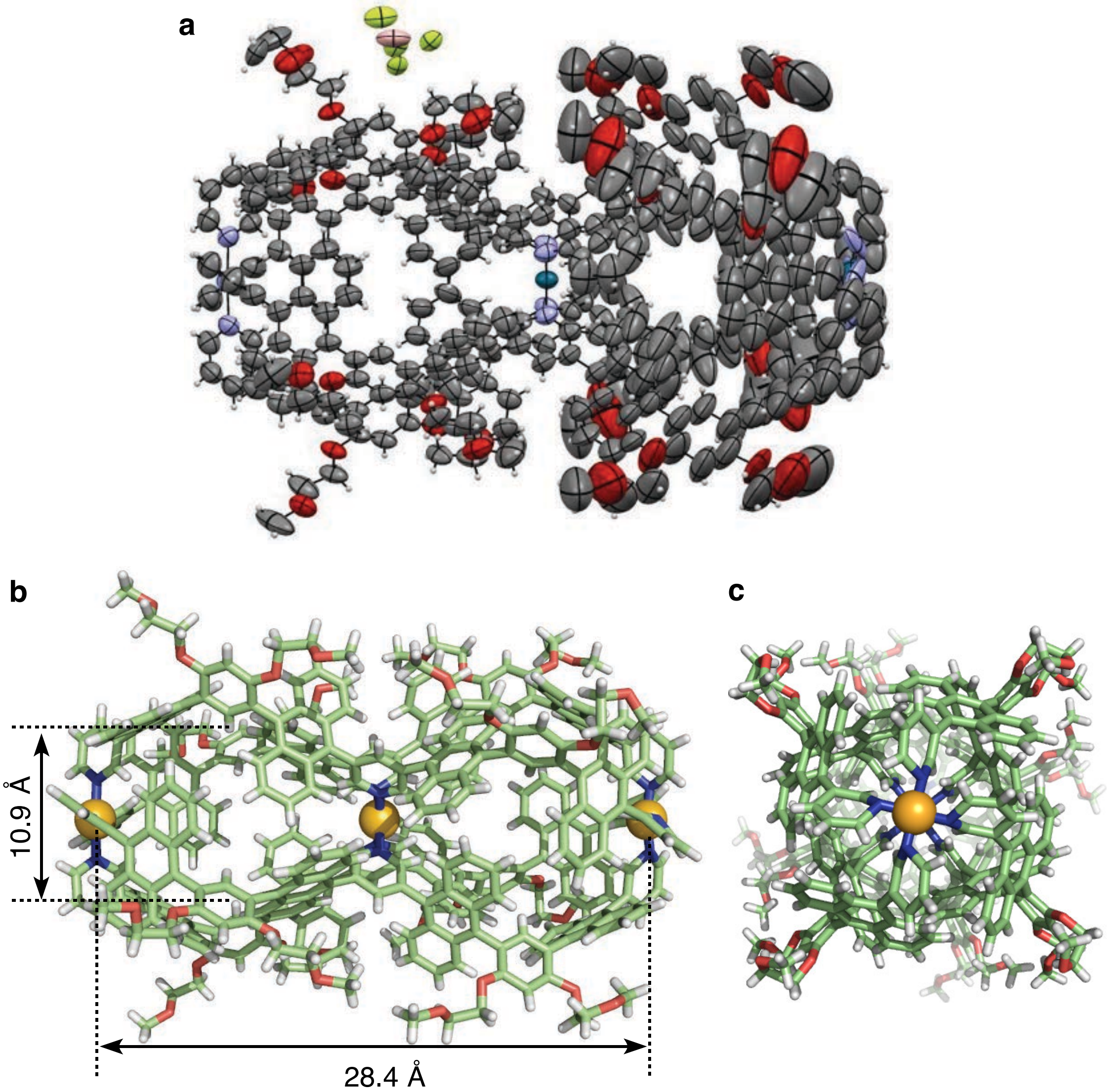

Supplementary Figure 60 | (a) ORTEP drawing of double capsule **2a'**. Ball-and-stick representation of (b) the side and (c) top views of **2a'**. The counterions are omitted for clarity.

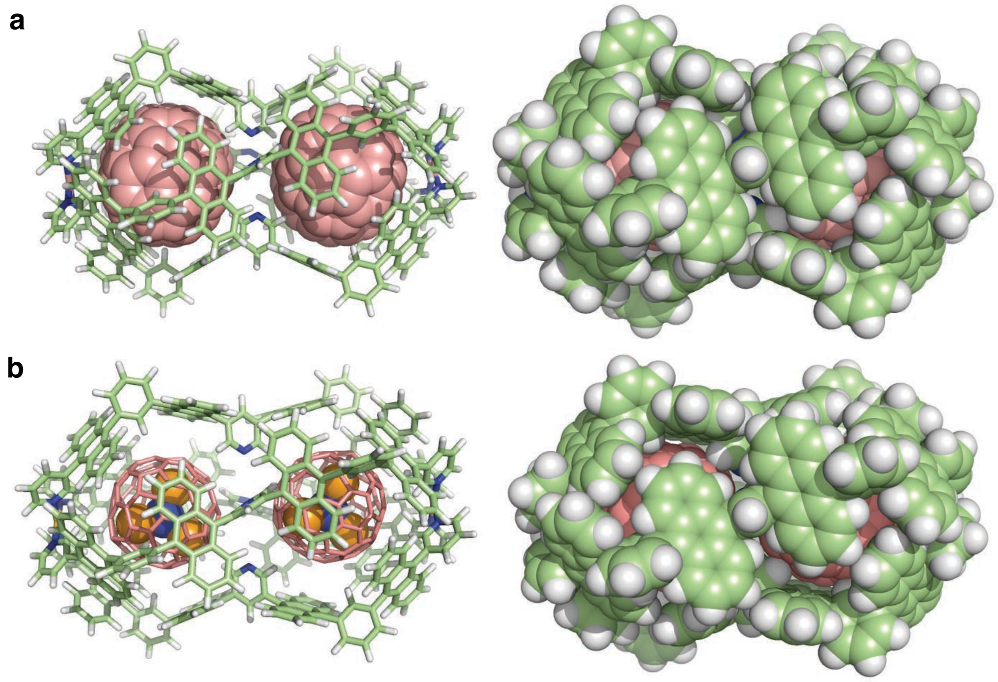

Supplementary Figure 61 | ** Optimized structures of (a) $(C_{70})_2$@3b** (R = -H) and (b) $(Sc_3N@C_{80})_2$@**3b** (R = -H) by molecular force-field calculation.

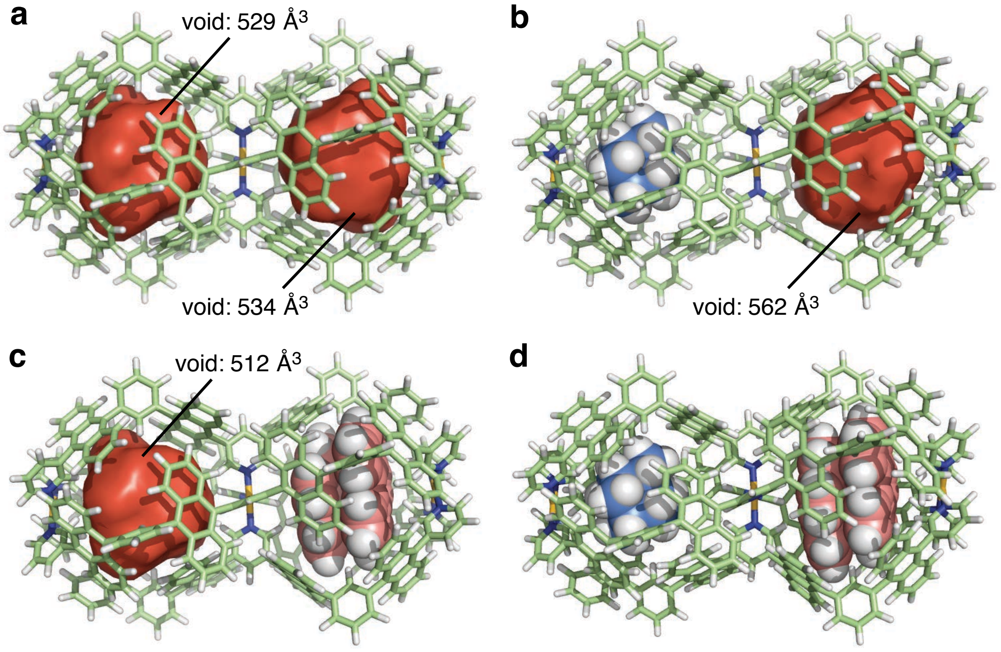

Supplementary Figure 62 | ** Optimized structures and calculated voids of (a) **2b (R = -H), (b) (**4a**/void)@**2b** (R = -H), (c) (void/(**4b**)$_2$)@**2b** (R = -H), and (d) ((**4a**/(**4b**)$_2$)@**2b** (R = -H) by molecular force-field calculation.

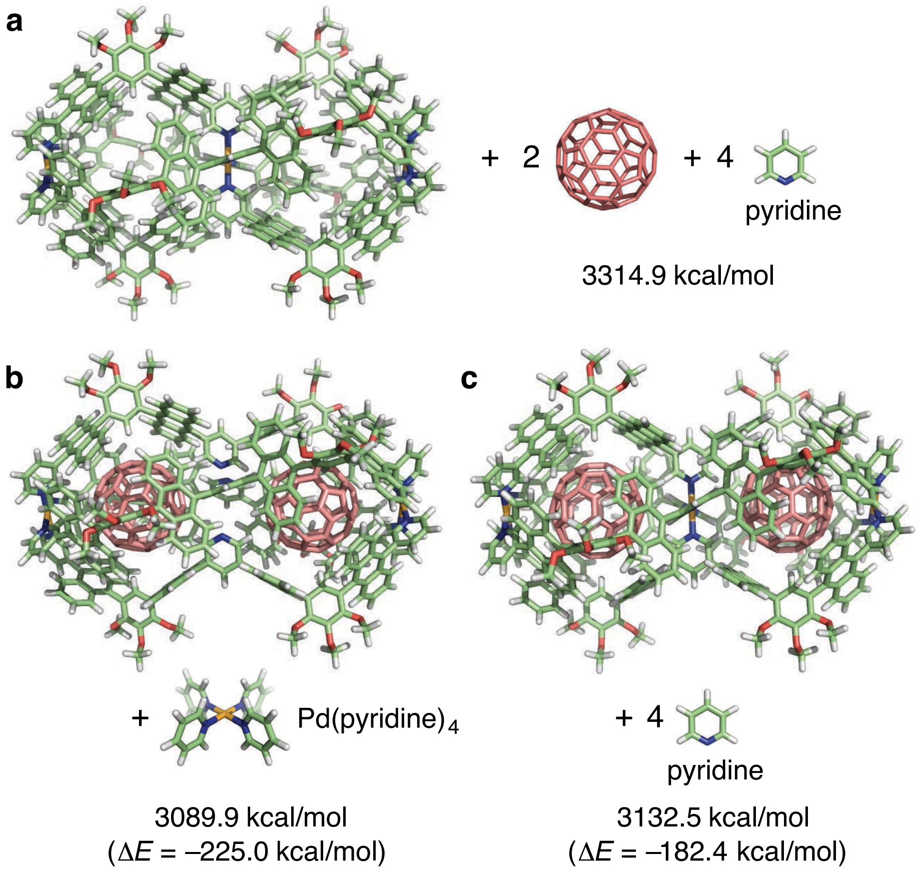

Supplementary Figure 63 | Calculated total energies of (a) **2b** (R = -OCH_3), two fullerenes, two DMSO, and four pyridines, and (b) $(C_{60})_2$@**3b** (R = -OCH_3) and Pd(pyridine)$_4$, and (c) $(C_{60})_2$@**2b** (R = -OCH_3) and four pyridines by molecular force-field calculation.

Supplementary Table

Supplementary Table 1 | Crystal data and structure refinement for **2a'**.

Identification code	KY781	
Empirical formula	C380 H300 B1 F4 N12 O32 Pd3	
Formula weight	5952.61	
Temperature	93 K	
Wavelength	1.54187 Å	
Crystal system	tetragonal	
Space group	P4/ncc	
Unit cell dimensions	a = 26.5390(8) Å	$\alpha = 90°$
	b = 26.5390(8) Å	$\beta = 90°$
	c = 71.806(7) Å	$\gamma = 90°$
Volume	50574(5) $Å^3$	
Z	4	
Density (calculated)	0.782 Mg/m^3	
Absorption coefficient	1.216 mm^{-1}	
F(000)	12396.0	
Crystal size	0.189 × 0.184 × 0.047 mm^3	
Theta range for data collection	7.55 to 149.276°.	
Index ranges	-33 <=h<=31, -29<=k<=32, -73<=l<=89	
Reflections collected	152900	
Independent reflections	25558 [R(int) = 0.0690]	
Completeness to theta = 67.687°	98.5 %	
Absorption correction	multi-scan	
Max. and min. transmission	0.944 and 0.678	
Refinement method	Full-matrix least-squares on F^2	
Data / restraints / parameters	25558 / 370 / 1024	
Goodness-of-fit on F^2	1.204	
Final R indices [I>2sigma(I)]	R_1 = 0.1060, wR_2 = 0.3412	
R indices (all data)	R_1 = 0.1548, wR_2 = 0.3855	
Largest diff. peak and hole	0.79 and -1.67 e.$Å^{-3}$	

The supplementary crystallographic data of **2a'** (CCDC 529139) can be obtained free of charge from the Cambridge Crystallographic Data Centre via www.ccdc.cam. ac.uk/data_request/cif. Disordered solvent molecules and counterions were removed by SQUEEZE program and the result was attached to the CIF file[1-6].

Supplementary Methods

Synthesis of compound 6a

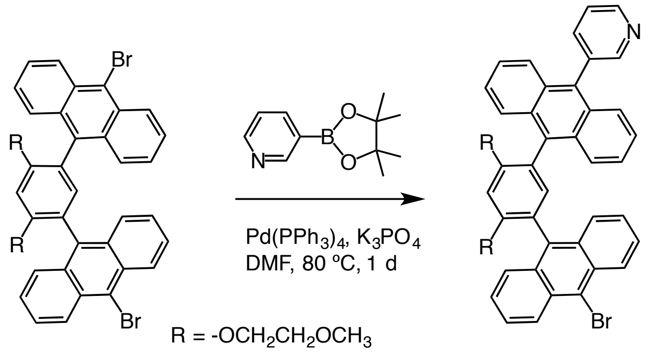

Compound **5a** (2.084 g, 2.829 mmol), 3-pyridineboronic acid pinacol ester (0.614 g, 2.96 mmol), K_3PO_4 (2.673 g, 12.59 mmol), and $Pd(PPh_3)_4$ (124 mg, 0.108 mmol) were added to a 300 mL glass flask filled with N_2. Dry DMF (50 mL) was added to the flask and then the mixture was stirred at 80 °C for 1 d. The resultant solution was concentrated under reduced pressure. After addition of water, the crude product was extracted with $CHCl_3$. The obtained organic layer was dried over $MgSO_4$, filtrated, and concentrated under reduced pressure. The crude product was purified by column chromatography (silica gel, ethyl acetate : hexane : $CHCl_3$ = 1 : 1 : 1) to afford compound **6a** as a yellow powder (0.819 g, 1.12 mmol, 39%) (see Supplementary Figs 2-6).

^{1}H NMR (500 MHz, $CDCl_3$, room temperature): δ 8.80 (d, J = 5.0 Hz, 1H), 8.74 (s, 0.5H), 8.65 (s, 0.5H), 8.58 (d, J = 9.0 Hz, 2H), 7.99 (d, J = 8.5 Hz, 2H), 7.96 (d, J = 9.0 Hz, 2H), 7.86 (d, J = 7.5 Hz, 0.5H), 7.77 (d, J = 7.0 Hz, 0.5H), 7.62-7.57 (m, 5H), 7.48-7.42 (m, 4H), 7.35 (dd, J = 9.5, 9.5 Hz, 2H), 7.24 (s, 1H), 7.12 (s, 1H), 4.14 (t, J = 5.0 Hz, 2H), 4.17-4.09 (m, 2H), 3.38 (t, J = 5.0 Hz, 2H), 3.32 (t, J = 5.0 Hz, 2H), 2.94 (s, 6H), 2.91 (s, 6H). ^{13}C NMR (125 MHz, $CDCl_3$, room temperature): δ 158.2 (C_q), 151.7 (CH), 148.7 (CH), 139.1 (CH), 136.8 (CH), 135.2 (C_q), 134.4 (C_q), 134.1 (C_q), 132.4 (C_q), 131.5 (C_q), 130.4 (2 × C_q), 127.9 (CH), 127.4 (CH), 127.2 (CH), 126.8 (CH), 126.2 (CH), 125.5 (2 × CH), 125.1 (2 × C_q), 123.5 (CH), 122.6 (C_q), 120.3 (C_q), 120.0 (C_q), 100.0 (CH), 70.7 (CH_2), 69.1 (CH_2), 59.0 (CH_3). FT-IR (KBr, cm^{-1}): 2925, 2879, 2817, 1604, 1506, 1451, 1439, 1376, 1360, 1335, 1303, 1265, 1192, 1162, 1128, 1105, 1058, 1025, 769, 758, 718, 686, 608. HR MS (ESI, CH_3OH): m/z Calcd. for $C_{45}H_{36}NO_4Br$: 736.1883, Found 736.1889 $[M + H]^+$.

Synthesis of compound 6b

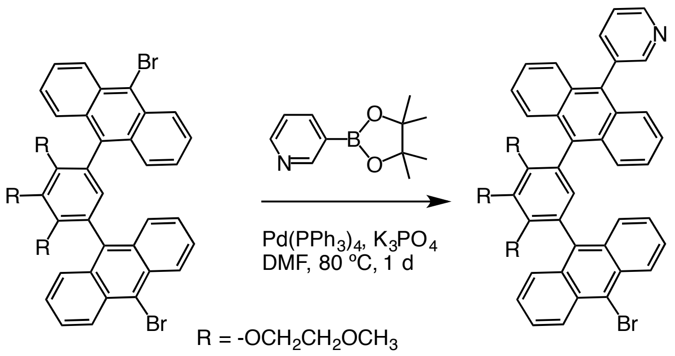

Compound **5b** (2.847 g, 2.945 mmol), 3-pyridineboronic acid pinacol ester (0.5957 g, 2.904 mmol), K_3PO_4 (2.415 g, 11.38 mmol), and $Pd(PPh_3)_4$ (100.2 mg, 0.8675 mmol) were added to a 300 mL glass flask filled with N_2. Dry DMF (50 mL) was added to the flask and then the mixture was stirred at 80 °C for 1 d. The resultant solution was concentrated under reduced pressure and then washed by water. The crude product was purified by column chromatography (silica gel, ethyl acetate : hexane = 1 : 1) to give compound **6b** and its debrominated compound. A mixture of the resultant powder and DBH was stirred in CH_2Cl_2 (20 mL) for 1 night. The resultant solution was washed with water to give **6b** as yellow powder (0.7843 g, 0.979 mmol, 33%) (see Supplementary Figs 19-22).

^{1}H NMR (500 MHz, $CDCl_3$, room temperature): δ 8.83 (d, J = 4.5 Hz, 1H), 8.75 (s, 0.5H), 8.67 (s, 0.5H), 8.62 (d, J = 9.0 Hz, 2H), 7.97 (m, 4H), 7.85 (d, J = 8.0 Hz, 0.5H), 7.77 (d, J = 8.0 Hz, 0.5H), 7.63-7.55 (m, 5.0H), 7.53-7.49 (m, 4.0H), 7.39 (dd, J = 7.5, 7.5 Hz, 2H), 7.07 (s, 0.5H), 7.06 (s, 0.5H), 4.52-4.51 (m, 2H), 3.98 (t, J = 4.5 Hz, 2H), 3.92 (br, 2H), 3.86 (t, J = 4.0 Hz, 2H), 3.47 (s, 3H), 3.09-3.06 (m, 2H), 3.01 (br, 2H), 2.79-2.78 (m, 6H). ^{13}C NMR (125 MHz, $CDCl_3$, room temperature): δ 152.5 (C_q), 152.4 (C_q), 152.2 (C_q), 151.7 (CH), 151.7 (CH), 148.8 (CH), 145.8 (C_q), 145.7 (C_q), 139.1 (CH), 139.0 (CH), 135.0 (C_q), 134.1 (C_q), 134.0 (C_q), 133.8 (C_q), 133.7 (C_q), 132.8 (C_q), 131.3 (C_q), 130.5 (CH), 130.4 (CH), 130.3 (C_q), 130.2 (C_q), 128.0 (CH), 127.6 (C_q), 127.3 (CH), 127.2 (CH), 127.0 (CH), 126.9 (CH), 126.3 (CH), 125.9 (CH), 125.6 (CH), 125.5 (CH), 123.5 (CH), 123.4 (CH), 123.0 (C_q), 72.9 (CH_2), 72.8 (CH_2), 72.1 (CH_2), 71.4 (CH_2), 71.3 (CH_2), 59.0 (CH_2), 58.3 (CH_3). FT-IR (KBr, cm^{-1}): 2979, 2925, 2881, 2818, 1464, 1438, 1416, 1389, 1348, 1256, 1130, 1106, 1070, 1026, 926, 909, 759. MALDI-TOF MS (dithranol): m/z Calcd. for $C_{48}H_{42}NO_6$: 810.23, Found 810.42 $[M + H]^+$. HR MS (ESI): m/z Calcd. for $C_{48}H_{42}NO_6$: 810.2251, Found 810.2219 $[M + H]^+$.

Synthesis of W-shaped ligand 1b

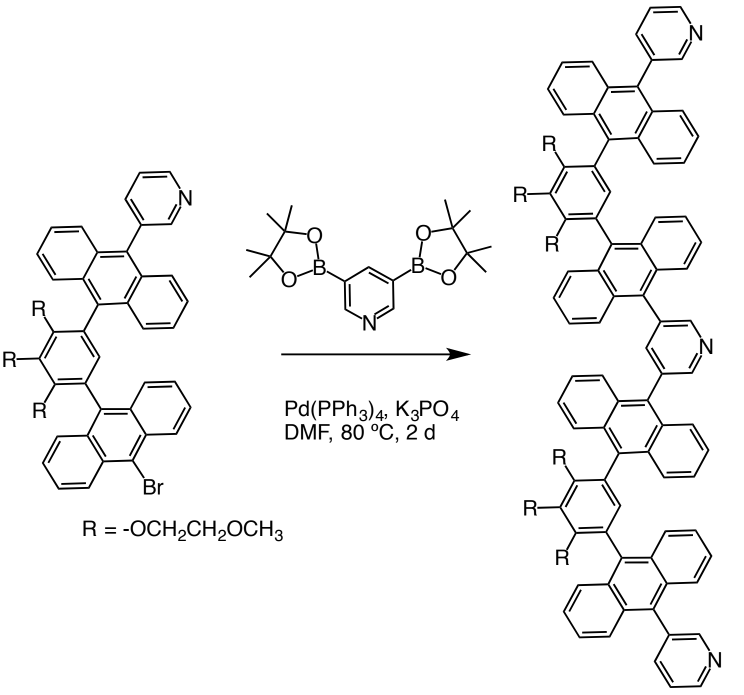

Compound **6b** (1.3568 g, 1.6775 mmol), 3,5-pyridinediboronic acid bis(pinacol) ester (194.6 mg, 0.58 mmol), K_3PO_4 (1.04 g, 4.9 mmol), and $Pd(PPh_3)_4$ (90.5 mg, 78.0 μmol) were added to a 300 mL glass flask filled with N_2. Dry DMF (100 mL) was added to the flask and then the mixture was stirred at 80 °C for 2 d. After addition of water (200 mL), the precipitation was collected by the filtration. The crude product was purified by gel permeation chromatography to afford ligand **1b** as a yellow powder (345.1 mg, 0.2248 mmol, 39%) (see Supplementary Figs 23-26).

^{1}H NMR (500 MHz, $CDCl_3$, room temperature): δ 8.96-8.60 (m, 6H), 8.06-7.34 (m, 37H), 7.16-7.08 (m, 2H), 4.56-4.54 (m, 4H), 4.01-3.87 (m, 12H), 3.50-3.44 (m, 6H), 3.14-3.07 (m, 8H), 2.86-2.56 (m, 12H). ^{13}C NMR (100 MHz, $CDCl_3$, room temperature): δ 152.4 (CH), 151.8 (C_q), 151.1 (CH), 148.9 (CH), 145.7 (C_q), 141.9 (CH), 138.9 (CH), 134.9 (C_q), 134.7 (C_q), 134.4(C_q), 134.1 (C_q), 132.8 (C_q), 132.4 (C_q), 130.6 (CH), 130.20 (C_q), 127.6 (CH), 127.2 (CH), 127.1 (CH), 126.3 (CH), 125.9 (CH), 125.5 (CH), 123.4 (CH, C_q), 72.9 (CH_2), 72.7 (CH_2), 72.1 (CH_2), 71.4 (CH_2), 59.0 (CH_3), 58.2 (CH_3). FT-IR (KBr, cm^{-1}): 2926, 2877, 2814, 1440, 1415, 1394, 1375, 1356, 1245, 1196, 1129,1104, 1068, 1025, 927, 767, 718, 650. HR MS (ESI, CH_2Cl_2:CH_3OH): *m/z* Calcd. 1557.6215, Found 1557.6214 $[M + Na]^+$.

Formation of double capsule 2b

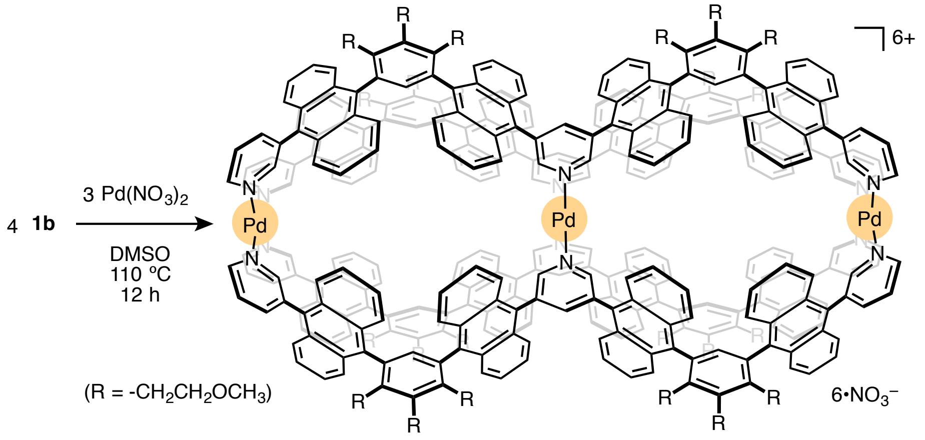

W-shaped ligand **1b** (91.8 mg, 59.8 μmol), a DMSO-d_6 solution (50 mM) of $Pd(NO_3)_2$ (0.93 mL, 46.4 μmol), which prepared *in situ* from $PdCl_2(DMSO)_2$ and $AgNO_3$, and DMSO-d_6 (4 mL) were added to a glass test tube and then the mixture was stirred at 110 °C for 12 h. The quantitative formation of double capsule **2b** was confirmed by NMR and MS analyses (see Supplementary Figs 27-33).

^{1}H NMR (500 MHz, D_2O:CD_3CN = 5:1, room temperature): δ 9.32 (d, J = 5.5 Hz, 2H), 9.25 (s, 2H), 8.81 (s, 2H), 8.73 (d, J = 5.5 Hz, 2H), 8.56 (dd, J = 5.5, 5.5 Hz, 2H), 8.31 (d, J = 6.8 Hz, 2H), 8.26 (d, J = 8.5 Hz, 2H), 8.07 (d, J = 9.0 Hz, 2H), 8.04-7.89 (m, 10H), 7.70 (dd, J = 7.5, 7.0 Hz, 2H), 7.66 (d, J = 8.5 Hz, 2H), 7.45 (dd, J = 8.0, 7.0 Hz, 2H), 7.19 (dd, J = 7.0, 7.0 Hz, 2H), 7.09-7.00 (m, 6H), 6.69 (d, J = 8.0 Hz, 2H), 6.64 (s, 2H), 5.92 (dd, J = 8.5, 7.5 Hz, 2H), 4.72 (br, 2H), 4.57-4.54 (m, 2H), 4.21-4.19 (m, 2H), 4.13-4.01 (m, 10H), 3.55 (s, 6H), 3.28-3.25 (m, 2H), 3.20-3.17 (m, 2H), 3.06-3.04 (m, 4H), 2.70 (s, 6H), 2.57 (s, 6H). ^{13}C NMR (120 MHz, D_2O:CD_3CN = 5:1, room temperature): δ 151.6 (CH), 151.3 (CH, C_q), 150.4 (CH), 147.3 (CH), 144.9 (C_q), 144.0 (CH), 139.5 (C_q), 137.1 (C_q), 135.6 (C_q), 134.4 (C_q), 129.2 (CH), 128.9 (C_q), 128.8 (C_q), 128.6 (C_q), 128.4 (C_q), 128.0 (C_q), 127.7 (C_q), 127.4 (CH), 127.2 (CH), 126.5 (CH), 126.3 (CH), 124.9 (CH), 124.7 (CH), 124.0 (CH), 123.4 (CH), 122.6 (CH), 122.2 (CH), 71.8 (CH_2), 70.9 (CH_2), 69.9 (CH_2), 57.4 (CH_3), 56.4 (CH_3). DOSY NMR (500 MHz, DMSO-d_6, 298 K): $D = 6.31 \times 10^{-11} m^2 s^{-1}$. FT-IR (KBr, cm^{-1}): 3060, 2931, 2884, 2816, 1442, 1417, 1378, 1228, 1195, 1105, 1065, 1030, 944, 925, 886, 848, 829, 772, 707, 671, 643, 616. ESI-TOF MS (CH_3CN): m/z 1304.0 [**2b** – 5•NO_3^-]$^{5+}$, 1645.5 [**2b** – 4•NO_3^-]$^{4+}$, 2214.7 [**2b** – 3•NO_3^-]$^{3+}$.

Formation of $(C_{60})_2$@3a, $(C_{70})_2$@3b, and $(Sc_3N@C_{80})_2$@3b

$(C_{60})_2$@3a. ^{1}H NMR (500 MHz, DMSO, room temperature): δ 9.18 (d, J = 5.0 Hz, 2H), 8.67 (br, 4H), 8.51 (dd, J = 9.0, 5.0 Hz, 2H), 8.42 (d, J = 9.0 Hz, 2H), 8.30 (d, J = 8.0 Hz, 2H), 8.05 (br, 2H), 7.98 (d, J = 8.5 Hz, 2H), 7.91-7.86 (m, 4H), 7.78 (s, 1H), 7.71-7.69 (m, 4H), 7.61-7.50 (m, 10H), 7.20 (s, 2H), 7.02 (dd, J = 7.5, 6.0 Hz, 2H), 6.90 (d, J = 8.5 Hz, 2H), 6.84 (s, 2H), 6.57 (br, 2H), 6.29 (d, J = 8.0 Hz, 2H), 4.26 (br, 4H), 4.12 (br, 4H), 3.35 (br, 4H), 3.20 (br, 4H), 2.95 (s, 6H), 2.69 (s, 6H). ESI-TOF MS (CH_3CN) of $(C_{60})_2$@**3a**: *m/z* 1800.0 [$(C_{60})_2$@**3a** – 4•NO_3^-]$^{4+}$, 2420.7 [$(C_{60})_2$@**3a** – 3•NO_3^-]$^{3+}$.

$(C_{70})_2$@3b. ^{1}H NMR (500 MHz, DMSO, room temperature): δ 9.38 (d, J = 5.0 Hz, 2H), 8.97 (s, 2H), 8.71 (d, J = 7.5 Hz, 2H), 8.60 (dd, J = 7.5, 5.0 Hz, 2H), 8.43-8.41 (m, 2H), 8.20 (s, 2H), 8.12 (s, 2H), 7.99 (d, J = 8.0 Hz, 2H), 7.90 (d, J = 8.5 Hz, 2H), 7.84 (br, 2H), 7.77 (br, 2H), 7.73 (dd, J = 8.5, 6.5 Hz, 2H), 7.62 (br, 2H), 7.51 (br, 6H), 7.07 (d, J = 8.0 Hz, 2H), 6.89 (dd, J = 8.0, 7.0 Hz, 2H), 6.57 (br, 4H), 6.46 (d, J = 8.0 Hz, 2H), 4.36-4.28 (m, 4H), 3.96-3.86 (m, 4H), 3.82-3.74 (m, 4H), 3.71 (t, J = 4.0 Hz, 4H), 3.32 (s, 6H), 3.01 (t, J = 5.0 Hz, 4H), 2.85 (t, J = 4.5 Hz, 4H), 2.62 (s, 6H), 2.55 (s, 6H). ESI-TOF MS (CH_3CN) of $(C_{70})_2$@**3b**: *m/z* 2008.3 [$(C_{70})_2$@**3b** – 4•NO_3^-]$^{4+}$, 2698.4 [$(C_{70})_2$@**3b** – 3•NO_3^-]$^{3+}$.

$(Sc_3N@C_{80})_2$@3b. ^{1}H NMR (500 MHz, DMSO, room temperature): δ 9.22 (d, J = 5.0 Hz, 2H), 8.73 (s, 2H), 8.55 (d, J = 6.0 Hz, 2H), 8.46 (br, 2H), 8.39 (d, J = 6.0 Hz, 2H), 8.20 (br, 2H), 8.16 (br, 2H), 8.10 (d, J = 7.0 Hz, 2H), 8.01 (d, J = 8.0 Hz, 2H), 7.89 (br, 3H), 7.84 (br, 2H), 7.79(br, 2H), 7.76 (br, 4H), 7.58 (br, 4H), 7.49 (d, J = 6.0 Hz, 2H), 7.13 (br, 2H), 6.88 (d, J = 7.0 Hz, 2H), 6.76 (s, 2H), 6.65 (br, 2H), 6.37 (d, J = 8.0 Hz, 2H), 4.39 (br, 4H), 3.95-3.90 (m, 8H), 3.75 (br, 4H), 3.35 (s, 6H), 3.00 (br, 4H), 2.92 (br, 4H), 2.60 (s, 6H), 2.55 (s, 6H). ESI-TOF MS (CH_3CN) of $(Sc_3N@C_{80})_2$@**3b**: *m/z* 2142.8 [$(Sc_3N@C_{80})_2$@**3b** – 4•NO_3^-]$^{4+}$, 2877.7 [$(Sc_3N@C_{80})_2$@**3b** – 3•NO_3^-]$^{3+}$.

Encapsulation of diamantane (4a) by 2b

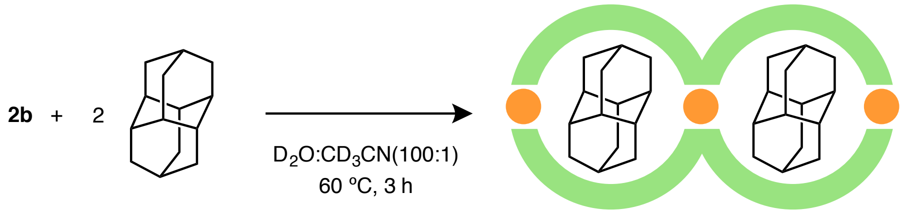

Diamantane (**4a**; 0.8 mg, 4.0 μmol) was added to a 100:1 $D_2O:CD_3CN$ solution (0.6 mL) of double capsule **2b** (1.5 mg, 0.22 μmol) and the mixture was stirred at 60 °C for 3 h. The quantitative formation of a (**4a**/**4a**)@**2b** complex was confirmed by NMR and ESI-TOF MS analyses (see Supplementary Figs 50-52). Similarly, two molecules of *p*-cyclophane (**4c**) were quantitatively encapsulated within **2b** to give a (**4c**/**4c**)@**2b** complex (see Supplementary Figs 53-55).

(4a/4a)@2b. DOSY NMR (500 MHz, $D_2O:CD_3CN$ = 100:1, 298 K): $D = 1.32 \times 10^{-10}$ m^2 s^{-1}. ESI-TOF MS ($H_2O:CH_3CN$ = 100:1): *m/z* 1139.1 [(**4a**/**4a**)@**2b** – 6•NO_3^-]$^{6+}$, 1379.3 [(**4a**/**4a**)@**2b** – 5•NO_3^-]$^{5+}$, 1739.6 [(**4a**/**4a**)@**2b** – 4•NO_3^-]$^{4+}$, 2340.1 [(**4a**/**4a**)@**2b** – 3•NO_3^-]$^{3+}$.

(4c/4c)@2b. DOSY NMR (500 MHz, $D_2O:CD_3CN$ = 100:1, 298 K): $D = 1.26 \times 10^{-10}$ m^2 s^{-1}. ESI-TOF MS ($H_2O:CH_3CN$ = 100:1): *m/z* 1145.8 [(**4c**/**4c**)@**2b** – 6•NO_3^-]$^{6+}$, 1387.3 [(**4c**/**4c**)@**2b** – 5•NO_3^-]$^{5+}$, 1749.6 [(**4c**/**4c**)@**2b** – 4•NO_3^-]$^{4+}$, 2353.5 [(**4c**/**4c**)@**2b** – 3•NO_3^-]$^{3+}$.

Encapsulation of phenanthrene (4b) by 2b

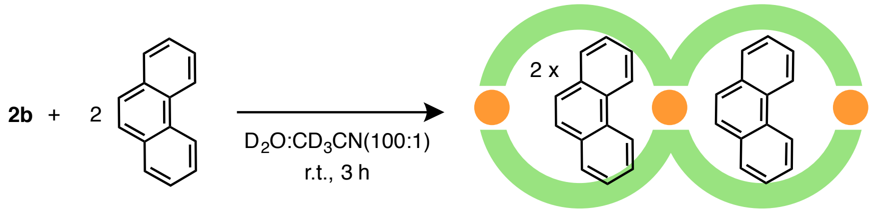

Phenanthrene (**4b**; 0.8 mg, 4.4 μmol) was added to a 100:1 $D_2O:CD_3CN$ solution (0.6 mL) of double capsule **2b** (1.5 mg, 0.22 μmol) and the mixture was stirred at room temperature for 3 h. The formation of a (**4b**/(**4b**)$_2$)@**2b** complex was confirmed by NMR and ESI-TOF MS analyses (see Supplementary Figs 56-57).

ESI-TOF MS ($H_2O:CH_3CN$ = 100:1): *m/z* 1165.4 [(**4b**/(**4b**)$_2$)@**2b** – 6•NO_3^-]$^{6+}$, 1410.9 [(**4b**/(**4b**)$_2$)@**2b** – 5•NO_3^-]$^{5+}$, 1779.1 [(**4b**/(**4b**)$_2$)@**2b** – 4•NO_3^-]$^{4+}$.

Supplementary References

1. Rigaku CrystalStructure, Version 4.2 (2015).
2. Rigaku REQAB (1998).
3. Sheldrick, G. M. A short history of *SHELX*. *Acta Cryst. A* **64**, 112-122 (2008).
4. Sheldrick, G. M. SHELXT: Integrating space group determination and structure solution. *Acta Cryst. A* **70**, C1437 (2014).
5. Vandersluis, P. & Spek, L. A. BYPASS: an effective method for the refinement of crystal structures containing disordered solvent regions. *Acta Cryst. A* **46**, 194–201 (1990).
6. Spek, A. L. Structure validation in chemical crystallography. *Acta Cryst. D* **65**, 148–155 (2009).
